# Therapies to limit myocardial injury in animal models of myocarditis: a systematic review and meta-analysis

**DOI:** 10.1007/s00395-019-0754-x

**Published:** 2019-10-31

**Authors:** Joshua A. Silverblatt, Oliver J. Ziff, Luke Dancy, Allen Daniel, Ben Carter, Paul Scott, Daniel M. Sado, Ajay Shah, Daniel I. Bromage

**Affiliations:** 1 School of Cardiovascular Medicine and Sciences, James Black Centre, King’s College London BHF Centre of Excellence, 125 Coldharbour Lane, London, SE5 9NU UK; 2 Institute of Cardiovascular Science, University College London, London, UK; 3 Department of Biostatistics and Health Informatics, Institute of Psychiatry, Psychology and Neuroscience, King’s College London, London, UK; 4 King’s College Hospital NHS Foundation Trust, London, UK

**Keywords:** Myocarditis, Remodelling, Drug treatment, Necrosis, Fibrosis, Calcification, Systematic review, Meta-analysis

## Abstract

Current myocarditis guidelines do not advocate treatment to prevent myocardial injury and scar deposition in patients with myocarditis and normal left ventricular ejection fraction. We aimed to ascertain the utility of beta blockers, calcium channel blockers and antagonists of the renin–angiotensin system in ameliorating myocardial injury, scar formation and calcification in animal in vivo models of myocarditis. The project was prospectively registered with the PROSPERO database of systematic reviews (CRD42018089336). Primary outcomes (necrosis, fibrosis and calcification) were meta-analysed with random-effects modelling. 52 studies were systematically reviewed. Meta-analysis was performed compared with untreated controls. In each study, we identified all independent comparisons of treatment versus control groups. The pooled weighted mean difference (WMD) indicated treatment reduced necrosis by 16.9% (71 controlled analyses, 95% CI 13.2–20.7%; *P* < 0.001), however there was less evidence of an effect after accounting for publication bias. Treatment led to a 12.8% reduction in fibrosis (73 controlled analyses, 95% CI 7.6–18.0%; *P* < 0.001). After accounting for publication bias this was attenuated to 7.8% but remained significant. Treatment reduced calcification by 4.1% (28 controlled analyses, 95% CI 0.2–8.0%; *P* < 0.0395). We observed significant heterogeneity in effect size in all primary endpoints, which was predominantly driven by differences between drug categories. Beta blockers and angiotensin-converting enzyme (ACE) inhibitors were the only agents that were effective for both necrosis and fibrosis, while only ACE inhibitors had a significant effect on calcification. This study provides evidence for a role for ACE inhibitors and beta blockers to prevent myocardial injury and scar deposition in in vivo models of myocarditis. There is a need for further well-designed studies to assess the translational application of these treatments.

**Electronic supplementary material:**

The online version of this article (10.1007/s00395-019-0754-x) contains supplementary material, which is available to authorized users.

## Introduction

Myocarditis is broadly defined as inflammation of the myocardium, diagnosed using histological and immunohistochemical criteria [1]. Although its aetiology and clinical presentation are heterogenous, viral infections are the most important cause of myocarditis in North America and Europe, and dilated cardiomyopathy can ensue [2]. For example, biopsy-proven myocarditis has been reported in 9–16% of adult patients with unexplained dilated cardiomyopathy, and is associated with poor prognosis [2–4].

Like the response to acute myocardial infarction, the pathogenesis of myocarditis relates to a robust inflammatory response. Initially, the noxious insult (typically infectious or autoimmune) initiates cellular necrosis. This stimulates the recruitment of circulating immune cells, which initiate the deposition of extracellular matrix and myocardial fibrosis. This, in turn, can result in left ventricular (LV) remodelling, progressive functional deterioration and, consequently, poor outcomes [3–5].

The presence of late gadolinium enhancement on cardiovascular magnetic resonance (CMR) imaging correlates with replacement fibrosis on histology in dilated cardiomyopathy [6, 7]. Late gadolinium enhancement in patients with myocarditis has also been associated with increased risk of major adverse cardiac events (MACE), even after correction for LV systolic function [8–10]. Myocardial fibrosis after myocarditis therefore appears to be an important therapeutic target in humans.

Animal in vivo myocarditis is typically induced by virus inoculation or immunization with cardiac myosin. Several pre-clinical studies have reported a beneficial effect of drug treatment on necrosis and fibrosis, including with beta blockers, calcium channel blockers (CCB) and antagonists of the renin–angiotensin system [RAS, including angiotensin-converting-enzyme (ACE) inhibitors, angiotensin receptor blockers (ARB), direct renin inhibitors and aldosterone antagonists (MRA)]. Furthermore, myocardial calcification is a common consequence of in vivo myocarditis, albeit infrequent in humans, which is commonly prevented by drug treatment in these studies.

Despite this evidence, there is a paucity of clinical trials and it is not known if treatment with these drug classes can prevent myocardial injury and scar formation in patients with myocarditis in the presence of normal LV ejection fraction. Consequently, current treatment recommendations in humans focus on supportive therapies, immunomodulation and immunosuppression [2, 11]. We performed a systematic review and meta-analysis to delineate the association between beta blockers, calcium channel blockers and RAS antagonists and necrosis, fibrosis and calcification in in vivo myocarditis. Furthermore, we aimed to examine determinants of efficacy of drug treatment in pre-clinical experiments to facilitate translation to clinical trials.

## Methods

The project was prospectively registered with the PROSPERO database of systematic reviews (CRD42018089336) and performed in accordance with Preferred Reporting Items for Systematic reviews and Meta-Analyses (PRISMA) guidelines [12].

### Search strategy and eligibility criteria

A systematic review of Medline (1946–26th February 2019) and Embase (1974–26th February 2019) was performed. Literature searches were conducted independently by JS and DB. The search strategy included keywords and MeSH terms relating to experimental myocarditis and treatment with beta blockers, calcium channel blockers and RAS antagonists (Supplementary material). This was developed by DB and JS using published guidelines [13–15], and peer reviewed by members of the King’s College London NHS Trust Heart Failure Unit. Our search was limited to reports available in English due to limited time and financial resources for translation. Review articles, abstract articles, unpublished material and ongoing studies were excluded. Duplicates were removed using Endnote (Thomas Reuters, US) and all remaining results subjected to eligibility screening.

Study eligibility criteria were defined using the PICOS approach [16]. In vivo animal studies were included if they investigated the effect of any of beta blockers, CCB and RAS antagonists (including ACE inhibitors, ARB, MRA and direct renin inhibitors) vs. control (sham treatment) on histological parameters of myocardial injury and scar formation, including necrosis, calcification or fibrosis, in any mammalian species with experimental myocarditis, regardless of study design.

Studies were excluded if they did not report a histological endpoint relating to scar formation (necrosis, calcification and fibrosis) [17–30]. Furthermore, only those using standard scoring criteria (see Araki et al. [31]) were included. Myocarditis induced by Chagas (*Trypanosoma cruzi*) was excluded, so were animals with co-morbidities or co-intervention other than the induction of myocarditis. Groups were excluded where an eligible medication was administered in combination with another medication (including other eligible medications) or procedure likely to affect the outcome. Finally, studies investigating neonatal animals (according to the study report) were excluded.

### Data collection, synthesis and study quality

**Fig. 1:**
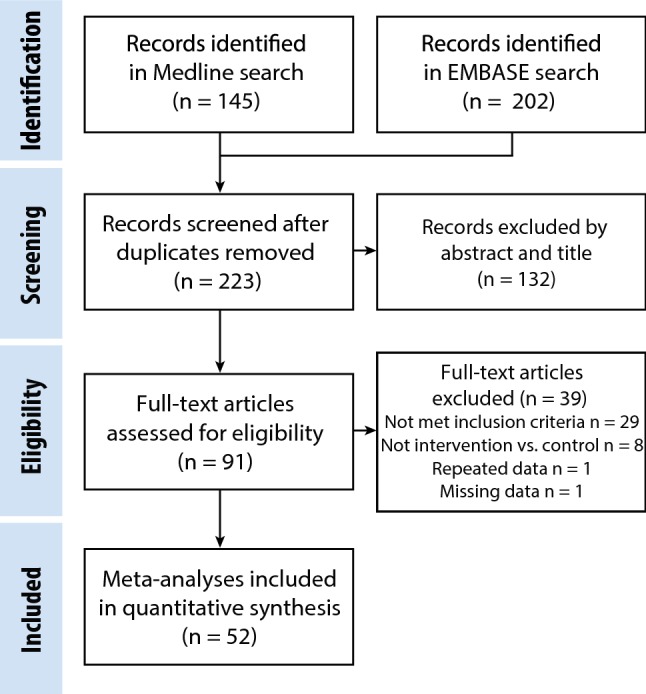
Flow chart of the study selection process. A systematic review yielded 347 reports. After removal of duplicates and the application of inclusion and exclusion criteria, 52 studies were included in the meta-analysis

Retrieved records were screened for eligibility using the title and abstract, followed by the full text (Fig. 1). Eligibility assessment was performed in an un-blinded, standardized manner (using predefined data fields) by JS and DB independently. All disagreements were resolved by examining the full text of the article and consensus between reviewers.

Variables for which data were sought were those that were considered likely to affect the efficacy of experimental myocarditis treatment. Data was independently extracted by two authors (JS and LD) using predefined data fields. We attempted to acquire missing information by contacting report authors [32–36]. Only experiments that were designed with homogeneity in population, intervention, control and outcome were considered for pooling [16]. All data items and assumptions are listed in Supplementary material.

Study level quality was assessed using SYRCLE’s risk of bias tool and an adapted CAMARADES checklist, both of which address key bias related to selection, performance, detection, attrition and reporting [37, 38]. Study quality was assessed independently from data extraction and between assessors in an un-blinded, standardized manner by OZ and AD. All disagreements were resolved by consensus. We examined available study protocols, study methods and results sections for selective reporting.

### Primary and secondary outcomes

The predefined primary outcomes were the following parameters of scar formation: necrosis, fibrosis and calcification. Secondary outcomes included survival, heart weight as a proportion of body weight (surrogate for LV mass), and inflammation.

### Statistical methods

#### Measuring treatment effect

Continuous outcome data was analysed as percentage of myocardium involved, enabling use of weighted mean difference (WMD, i.e., control group mean minus experimental group mean) between intervention and control groups, rather than standardised mean difference. In each study, we identified all independent comparisons of treatment versus control groups. To avoid statistical over-estimation, where multiple comparisons were made to the same control group, the size of the control group was corrected for the number of comparisons made (*n*/number of comparisons) [16, 39]. For studies describing more than one outcome, each outcome was analysed independently, and only one assessment of outcome was included per comparison.

#### Data synthesis

To account for anticipated heterogeneity, we pooled effect sizes using random-effects meta-analysis, which considers within-study and between-study variability and weights each study accordingly. Pooled effect size data for intervention and control groups were compared using the WMD and the corresponding standard deviation (SD), using the method of DerSimonian and Laird [40]. For the binary outcome of survival, pooled event data were compared using a relative risk (RR) with associated 95% confidence interval (CI).

#### Subgroup analysis, identification and explanation of heterogeneity

Heterogeneity was quantified using the Chi squared test, *T*^2^ and *I*^2^ statistics, and was considered significant if *I*^2^ > 75% [39, 41]. To look for sources of heterogeneity, outcomes were assessed according to predefined experimental factors and quality indicators. Subgroup analyses defined a priori were according to drug class, species, sex, method of myocarditis induction, and histology method.

#### Meta-regression and explanation of variability

Meta-regression was performed to assess the impact of the variables: timing of therapy, length of treatment and study quality, on the WMD. Primary meta-regression assessment used residual maximum likelihood with random-effects weighting and Knapp and Hartung *t*-distribution. Publication bias was assessed using Begg’s test and Egger’s test to identify small-study effects according to each of the outcomes assessed. If publication bias was indicated, we further evaluated the number of missing studies in a meta-analysis by the application of the trim-and-fill method and recalculated the pooled effect estimate with the addition of those missing studies [42]. A two-tailed *P* value of 0.01 was considered statistically significant. All analyses were performed with STATA (version 13.1) and R (version 3.5.1).

## Results

### Study selection

The search strategy identified 52 studies for systematic review (Fig. 1, Table 1). Overall, 24, 34 and 9 studies investigated necrosis, fibrosis and calcification, respectively. For secondary outcomes this was 31, 38 and 24 investigating survival, heart weight:body weight ratio(HW/BW) and inflammation, respectively.

**Table 1:** Summary of studies grouped according to outcome

Outcomes	Studies (*n*)	Analyses (*n*)	Treated animals	Control animals
Primary				
Necrosis	24	71	682	450
Fibrosis	34	73	842	502
Calcification	9	28	334	238
Secondary				
Survival	31	99	1918	1161
HW/BW	38	95	975	582
Inflammation	24	69	706	495

### Study characteristics

The main characteristics of included studies, and their references, are given in Supplementary material. All studies used hematoxylin and eosin (HE) staining for inflammation, calcification and necrosis, except for the study by Liu et al. that used Masson’s trichrome to measure necrosis [43]. Studies used one of three stains for fibrosis: Masson’s trichrome, Azan-Mallory or HE. Experimental animals were predominantly male, although studies of calcification used a mix of sexes. Studies typically induced myocarditis with a virus, except for survival and HW/BW (used a mix of viral and autoimmune induction) and fibrosis (more commonly autoimmune). Studies used either a mix of mice and rats (fibrosis, survival, HW/BW) or predominantly mice (necrosis, calcification, inflammation). With respect to drug class, most outcomes were assessed using a good range of drug classes. Notable exceptions are fibrosis, which included a small number of studies using CCB, MRA and direct renin inhibitors, and calcification, which was only tested with ACE inhibitors, ARB and beta blockers. Furthermore, MRA treatment was only used in a small number of studies investigating survival, fibrosis and HW/BW and, overall, few studies used direct renin inhibitor and CCB treatment. All analyses included a mix of manual and automatic measurement, except for necrosis, calcification and inflammation, which were mostly manual.

### Study quality and risk of bias

**Fig. 2:**
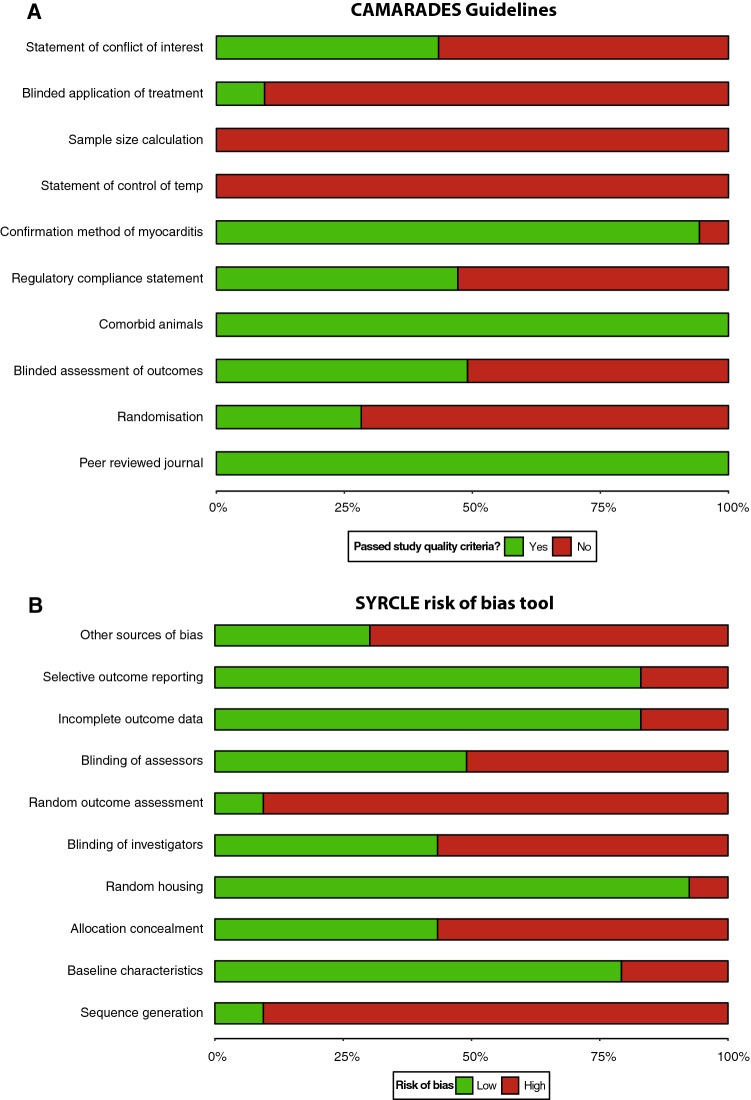
Reporting of study quality indicators. Study quality was assessed using the CAMRADES checklist (**a**) and SYRCLE risk of bias tool (**b**). Values are expressed as the percentage of studies reporting each quality indicator

Reports achieved a median SYRCLE risk of bias tool score of 5 (interquartile range 4–7; Fig. 2) out of 10 and a median adapted CAMARADES checklist score of 5 (interquartile range 4–5; Fig. 2). For example, using the SYRCLE risk of bias tool, only 9% reported random outcome assessment, 43% reported allocation concealment and 43% reported blinding to experimental protocol. Similarly, using the adapted CAMARADES checklist, no studies reported experimental temperature control or sample size calculation, and only 9% reported blinded application of treatment. A full breakdown of scores is included in the Supplementary material. Meta-regression indicated that neither the SYRCLE nor CAMARADES study quality score was associated with any of the endpoints tested (see Supplementary material).

### Meta-analysis of treatment efficacy

**Fig. 3:**
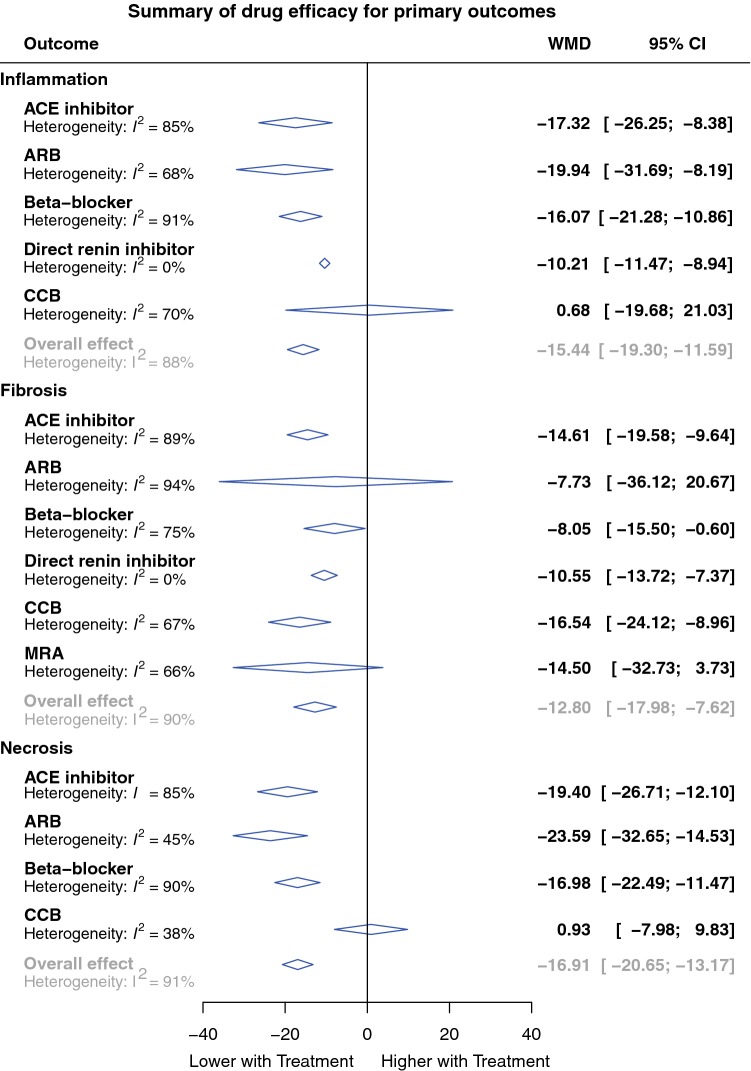
Summary plot of meta-analysis of drug efficacy for primary outcomes. Forest plots of the effect of eligible treatments on primary outcomes, pooled using random-effects meta-analysis. Overall, 172 controlled comparisons were included. The diamonds represent the pooled difference using a random-effects model. *I*^2^ is the percentage of total variation across studies due to heterogeneity. *CI* Confidence interval, *WMD* weighted mean difference

**Fig. 4:**
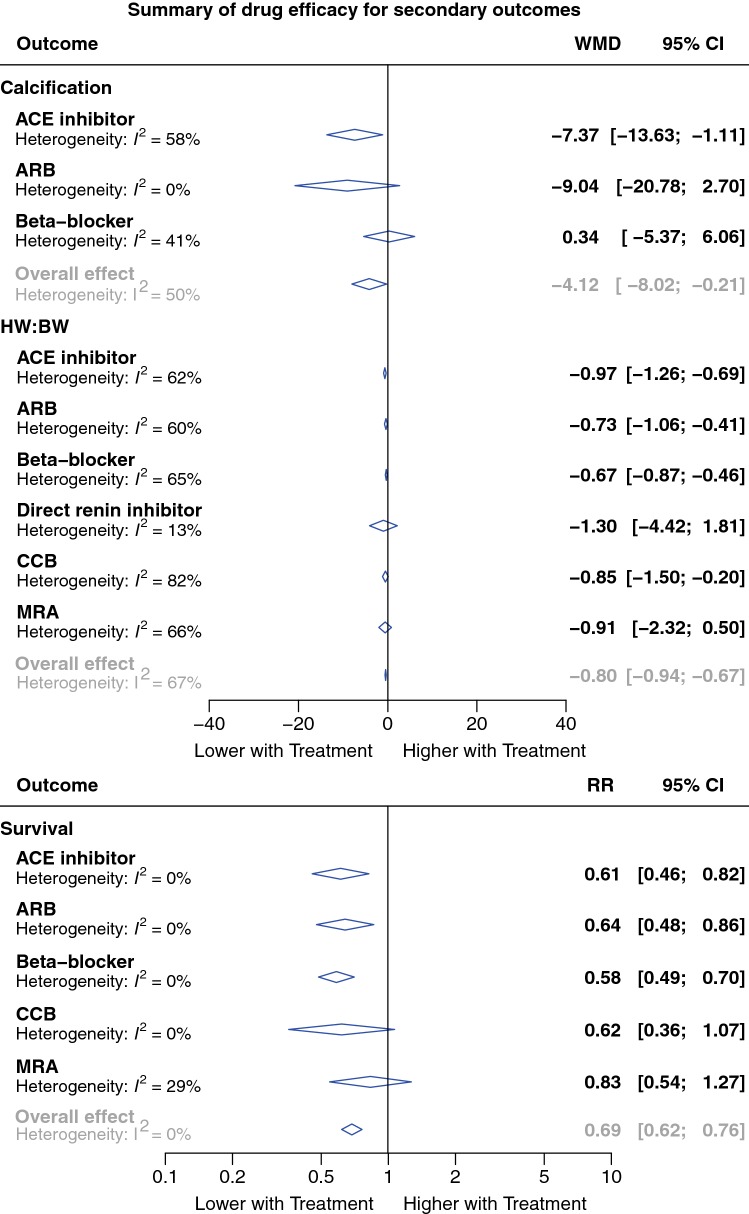
Summary plot of meta-analysis of drug efficacy for secondary outcomes. Forest plots of the effect of eligible treatments on secondary outcomes, pooled using random-effects meta-analysis. Overall, 263 controlled comparisons were included. The diamonds represent the pooled difference using a random-effects model. *I*^2^ is the percentage of total variation across studies due to heterogeneity. *CI* Confidence interval, *WMD* weighted mean difference, *RR* relative risk

All studies had extractable data and contributed to meta-analysis, which was performed for six outcomes: necrosis, fibrosis, calcification, inflammation, survival, HW/BW. A summary of the individual meta-analyses performed is presented in Figs. 3, 4 and detailed results are discussed below.

#### Necrosis

Data on 71 comparisons of drug intervention versus control was extracted. Overall, the included study drugs reduced necrosis by 16.9% (95% CI 13.2–20.7%) when compared to untreated controls (*P* < 0.001; Supplementary Fig. S1). Subgroup analysis according to drug class revealed a similar effect for all included classes with the exception of CCB, which was associated with a neutral effect on necrosis (0.9%; 95% CI − 8.0 to 9.8%; *P* = 0.80; *n* = 6; Fig. 3). Significant heterogeneity was observed in the overall analysis (*T*^2^ 124.2 and *I*^2^ 91.1%; *P* < 0.001). Subgroup analyses demonstrated that the main selected covariates contributing to this heterogeneity were drug class (residual *I*^2^ 86%; *P* < 0.01), sex (the mixed sex group had a lower pooled WMD than the male and female sex groups, residual *I*^2^ 89%; *P* < 0.01) and measurement method (the automatic measurement group had a lower pooled WMD than the manual and ‘not stated’ groups, residual *I*^2^ 90%; *P* < 0.01). Meta-regression did not show any impact of timing of therapy, length of treatment or study quality on WMD (Supplementary Tables S11–12).

#### Fibrosis

Data on 73 controlled comparisons was extracted. Overall, the included study drugs reduced fibrosis by 12.8% (95% CI 7.6–18.0%; *P* < 0.001), when compared to untreated controls (*P* < 0.001; Supplementary Fig. S2). With respect to drug class, a similar effect was observed for all included classes with the exception of ARBs (7.7%; 95% CI − 20.7 to 36.1%; *P* = 0.57; *n* = 19) and MRAs (14.5%; 95% CI −3.7 to 32.7%; *P* = 0.08; *n* = 3; Fig. 3). Significant heterogeneity was observed in the overall analysis (*T*^2^ 98.0 and *I*^2^ 90.4%; *P* < 0.001). The main selected covariates contributing to heterogeneity were drug class (residual *I*^2^ 89%; *P* < 0.01), species (rats had a higher pooled WMD than mice, residual *I*^2^ 88%; *P* < 0.01) and myocarditis induction method (the cardiac myosin and clozapine groups had higher pooled WMD, residual *I*^2^ 87%, *P* < 0.01). In addition, post hoc analysis of staining method showed that studies using Masson’s trichrome did not reach significance (residual *I*^2^ 88%, *P* < 0.01; Supplementary Fig. S3). Meta-regression did not show any impact of timing of therapy, length of treatment or study quality on WMD (Supplementary Tables S13–14).

#### Calcification

Data on 28 controlled comparisons was extracted. Overall, the included study drugs reduced calcification by 4.1% (95% CI 0.2–8.0%) when compared to untreated controls (*P* < 0.001; Supplementary Fig. S4). With respect to drug class, only ACE inhibitors achieved significance (7.4%; 95% CI 1.1–13.6%; *P* = 0.02; *n* = 13; Fig. 3). In the overall analysis, there was no significant heterogeneity (*T*^2^ = 26.0 and *I*^2^ = 50.1%; *P* = 0.001) and meta-regression did not show any impact of timing of therapy, length of treatment or study quality on WMD (Supplementary Tables S15–16).

#### Secondary outcomes

After initial data extraction, it was apparent that most studies included the additional outcome measures survival, HW/BW and inflammation. Although these are not directly related to myocardial injury and scar deposition they are of considerable interest and we therefore amended our protocol to include them as secondary endpoints. There were 99, 95 and 69 controlled comparisons for meta-analysis of survival, HW/BW and inflammation, respectively. Overall, treatment improved survival (RR 0.69, 95% CI 0.62–0.76; *P* < 0.001), HW/BW (WMD 0.8; 95% CI 0.7–0.9; *P* < 0.001) and inflammation (WMD 15.4%; 95% CI 11.6–19.3%; *P* < 0.001), compared to untreated controls (Supplementary Figs. S5–7). With respect to drug class, the beneficial effect on survival was not seen with CCBs [RR 0.62 (0.36–1.07); *P* = 0.08; Fig. 4, Supplementary Table S17] or MRAs (RR 0.83 (0.54–1.27); *P* = 0.26; Supplementary Table S17), and the improvement in inflammation was not seen with CCBs (WMD − 0.68%, 95% CI − 21.0 to 19.7%; *P* = 0.93; Supplementary Table S21). Significant heterogeneity was observed in the overall analysis only for inflammmation (*T*^2^ = 149.2 and *I*^2^ = 87.9%; *P* < 0.001) and the main tested covariate contributing to this was sex (the mixed sex group had a lower pooled WMD than the male and female sex groups, residual *I*^2^ 88%, *P* < 0.01). Meta-regression of the effect of timing of starting treatment confirmed that studies where treatment was started earlier had a greater association between drug intervention and improvement in inflammation (*β* = 0.78, *P* = 0.023). Detailed results are given in Supplementary Tables S17–22).

### Publication bias

There was evidence of small study effects and publication bias for the necrosis outcome (*P* < 0.001; Supplementary Fig. S8). The application of the trim and fill method to recalculate the pooled effect estimate attenuated the WMD to 4.5% (95% CI − 0.3 to 9.2%), with less evidence of an effect (*P* = 0.06), indicating that the results were affected by publication bias. The fibrosis outcome showed similar evidence for small study effects and publication bias (*P* = 0.039; Supplementary Fig. S9); however, the trim and fill method imputed a WMD of 7.8% (95% CI 2.6–13.0%) and although this attenuated the treatment effect, it remained significant (*P* = 0.004).

There was no evidence of small study effects or publication bias in the calcification or inflammation outcomes (Supplementary Figs. S10–11), while evidence for HW/BW and survival was mixed (Supplementary Figs. S12–13) For HW/BW, trim and fill resulted in WMD of 0.6 (95% CI 0.5–0.8; *P* < 0.0001), and for survival trim and fill resulted in a relative risk of 0.7 (95% CI 0.6–0.8; *P* < 0.0001), which both remain significant.

## Discussion

We aimed to describe the effect of drug treatment on important parameters of myocardial injury and scar formation in in vivo animal models of myocarditis using a systematic review and meta-analysis. We found a reduction in necrosis, fibrosis and calcification with therapy, compared to untreated control animals with myocarditis, based on data from 52 studies and 2220 animals. There were similar beneficial effects on the secondary outcomes of survival, HW/BW and inflammation. However, the significant impact on necrosis was less evident after correction for publication bias. Nonetheless, these are important observations in the context of the paucity of recommendations for prevention of myocardial injury and scar deposition after myocarditis in patients with normal LV systolic function in international guidelines [2].

### Study characteristics

Experimental animals were predominantly male and most induced myocarditis by injection of the cardiotropic viruses Coxsackievirus B3 (CVB3) or encephalomyocarditis virus (EMCV), which directly mediate focal necrosis and is appropriate given that viral infections are the most important cause of myocarditis in North America and Europe [2, 44, 45].

Most outcomes were assessed using a good range of drug classes. Exceptions are that MRA treatment was only used in a small number of studies investigating survival, fibrosis and HW/BW. This is surprising as MRA therapy has the best evidence for treating fibrosis in the general heart failure literature [46]. Aldosterone stimulates fibrosis via Nox2-containing nicotinamide adenine dinucleotide phosphate (NADPH) oxidase [47], and MRAs have been shown to abrogate this effect in several pre-clinical studies and prospective randomized trials in heart failure [48–53]. Similarly, few studies used direct renin inhibitor or CCB treatment.

With respect to histological stain, most studies used HE. While inflammation was not defined a priori, all studies used HE stains for this outcome and quantified mononuclear and polynuclear cellular infiltration. For fibrosis, studies used a mix of stains. Masson’s trichrome and Azan-Mallory are predominantly used for differentiating muscle and collagen, are typically used in studies of fibrosis, while HE is less specific. However, post hoc analysis of staining method showed a similar overall trend regardless of stain. Although this did not reach significance for Masson’s trichrome, this is likely to relate to the small group size.

### Study quality and risk of bias

Studies had mixed methodological quality that, together with publication bias, can lead to over-estimation of effect size [54–56], although no statistical association was evident using meta-regression. Confirmation of myocarditis was generally well reported which is reassuring. In addition, there was only infrequent selective outcome reporting and incomplete outcome data. However, there was only mixed observation of CAMARADES and SYRCLE guidelines and the worst-performing criteria typically related to study design, including sample size calculation, sequence generation, blinding and random allocation. In some cases, this will reflect unsatisfactory reporting (despite the criteria being met) but it appears more likely that studies are excessively biased due to omission of crucial elements of study design. These elements are particularly important as it is known that failure to do them can significantly increase effect size [57].

### Treatment efficacy and sources of heterogeneity

There were high levels of heterogeneity between studies investigating necrosis, fibrosis, and inflammation, which should therefore be interpreted with caution. To explore factors that could account for heterogeneity, and to elucidate any determinants of drug efficacy, we performed subgroup analysis of a priori variables that we hypothesised were likely to impact on the efficacy of experimental myocarditis drug treatment. The approach of identifying variables that can influence an intervention’s efficacy may inform clinical study design and improve attempts to translate novel therapies. For example, a meta-analysis of determinants of efficacy of cardiac ischaemic preconditioning in animal studies suggested attenuated efficacy in comorbid animals, which may have important implications for clinical study design [58]. In the present study, the beneficial effects of drug treatment were generally present across all subgroups and outcomes. The important exception to this was drug class. This is likely to relate to a range of variability in efficacy within drug classes, depending on the specific agent used and its possible off-target effects, as well as some important differences between classes.

#### Drug class

The largest effect on necrosis was seen with ARB treatment and, although the mechanism for this is not clear, it has been attributed to attenuation of virus-mediated oxidative stress [59]. Treatment with beta blockers and ACE inhibitors was also effective in this context.

MRA treatment was only used in two studies investigating fibrosis in the present analysis and, where it was, it had no significant effect on fibrosis. In addition to the paucity of studies investigating CCB and direct renin inhibitor treatment effects on fibrosis, this may account for heterogeneity related to drug class. Most of the available literature has investigated MRA therapy in heart failure secondary to ischaemic heart disease and it may be that there are systematic differences between this and myocarditis-induced fibrosis, although this has not been specifically investigated. ARB treatment was similarly neutral. In contrast, beta blockers and ACE inhibitors were beneficial, while the greatest effect was seen with CCB treatment. This may relate to direct inhibition of fibroblast activation by dihydropyridine calcium channel blockers, possibly by inhibition of transforming growth factor-β [60, 61], tumour necrosis factor-α or inducible nitric oxide synthase [62]. However, it should be noted that a clinical trial of a dihydropyridine CCB (amlodipine) in chronic heart failure was neutral [63]. Furthermore, non-dihydropyridine CCBs to prevent ventricular remodelling after acute myocardial infarction in the context of LV impairment have been associated with harm [64], despite similarly promising pre-clinical evidence [65].

Finally, drug class also contributed to significant heterogeneity in the inflammation outcome. Here, ACE, ARB, beta blocker and direct renin inhibitor treatment was effective in reducing inflammation in in vivo models of myocarditis. CCB treatment was ineffective at attenuating inflammation but was only tested in a small number of studies and this should be interpreted with caution. Interestingly, the majority of evidence for immunosuppression relates to virus-negative myocarditis and this is reflected in trials registered on ClinicalTrials.gov and clinical guidelines [2]. This is supported by neutral results in trials recruiting patients with unknown aetiology [66]. In contrast, the majority of studies in our meta-analysis investigated inflammation in virus-induced myocarditis models and found a reduction in the primary outcome measures despite significant anti-inflammatory effects. Furthermore, several studies measured virus replication and found no difference versus control despite evidence of reduced inflammation [67–70]. Taken together this suggests that, whatever the aetiology of myocarditis, treatment to prevent myocardial injury and fibrosis may be beneficial.

Overall, it appears ACE inhibitor and beta blocker treatment might have the broadest effectiveness in preventing the most clinically relevant outcomes of necrosis and fibrosis in experimental myocarditis. ACE inhibition also prevented myocardial calcification. This is a rare clinical manifestation of myocarditis, largely confined to case reports, that has been associated with poor outcomes [71]. The positive findings for ACE inhibitors and beta blockers complement the established literature for these drugs in chronic heart failure, most of which comes from their use after myocardial infarction. However, no clinical trials have specifically investigated their use in the context of myocarditis in the absence of LV systolic dysfunction and only trials investigating specific immunosuppressive therapies are registered on ClinicalTrials.gov. The only exception is the MIRACLE HIV study, which is studying the efficacy of eplerenone in patients with HIV on myocardial inflammation and fibrosis, but this is not a study of patients with myocarditis per se [72].

#### Animal sex

The second variable that influenced some outcomes was animal sex. Specifically, drug treatment had no significant impact on necrosis and inflammation in experiments using mixed sex experimental groups [33–35, 73]. However, no difference in drug efficacy was seen between male and female groups, and this finding should be interpreted with caution in view of the small number of comparisons, from only two research groups, available for subgroup analysis.

#### Timing of treatment

Finally, we found that improvement in inflammation, and a trend for fibrosis, were associated with the timing of starting treatment. This suggests that the earlier treatment was started, the larger the effect. Although the drug classes we analysed are not anti-inflammatory per se, the anti-inflammatory effect we reported in this analysis has, for example, been attributed to reduced EMCV-induced inflammation by impeding interleukin-1 production in monocytes [74], and regulation of inflammatory cytokine expression from T cells [75]. Given that the inflammatory response to injury peaks in the first 3 days after injury, it follows that early treatment is likely to have the biggest effect.

### Publication bias

There was evidence of small study effects and publication bias in several outcomes, which may affect the translational application of these findings. With respect to necrosis, while visual analysis of the funnel plot is reassuring, the application of the trim and fill method abrogated a significant change in the WMD with drug therapy. Funnel plot asymmetry was most marked in the fibrosis data, indicating the absence of small studies showing an improvement with drug treatment, suggesting that not all the relevant studies have been reported or included in the meta-analysis. However, after application of the trim and fill method, the treatment effect remains significant and may be expected to be more so should small positive studies be included. With respect to survival, visual assessment of the funnel plot suggests that small, negative studies may be under-represented. However, after application of the trim and fill method the treatment effect remains significant. In addition to true publication bias, funnel plot asymmetry can also result from study heterogeneity, which is a relevant consideration in the present meta-analysis [76].

### Strengths and limitations

To our knowledge, this is the first systematic review and meta-analysis investigating non-immunotherapy treatment strategies in myocarditis using in vivo animal models. We included many studies and animals in intervention and control arms, which allowed for robust analysis of subgroups. Our review is based on reported results of independent published studies, prepared according to explicit reproducible methods.

With respect to limitations, first, there was significant heterogeneity between studies in several analyses. We have accounted for this, at least in part, by using random-effects, subgroup analyses and meta-regression. However, there is residual heterogeneity that is likely to relate to other, unmeasured, variables as well as reflecting the inclusion of small, non-randomized studies and a degree of caution is necessary when interpreting the findings [77]. Second, no studies documented LV function at start of the experiment. All studies used healthy mice and we excluded studies that included co-morbid animals, so it is reasonable to assume that baseline LV function was normal. In fact, very few studies examined cardiac morphology and function at all, although those that did typically reported worsening LV systolic function after myocarditis induction and at least partial recovery with the experimental drug [61, 62, 78–82]. Third, as with all meta-analyses, the quality of reporting of the included studies is crucial to its validity, therefore studies with missing data were excluded. However, several studies did not meet important quality indices, especially with respect to including sample size calculation, sequence generation, blinding and random allocation and, although study quality was not related to any outcomes, it has been asserted that poorly performed animal studies should be interpreted with caution, especially when used as a rationale for human trials. Fourth, the included literature was dated, which reflects trends in cardiovascular research whereby more contemporary studies have investigated immunotherapy for virus-negative myocarditis. Fifth, only English language publications were considered. Sixth, it is important to acknowledge the possible impact of co-morbidities and other medications on the efficacy of drug treatment in myocarditis. Pre-clinical animal models used frequently over-simplify complex co-morbidity, risk factor and medication profiles of humans with myocarditis [83]. Studies using animals with co-morbidities were specifically excluded due to the limited number of available studies. Nonetheless, co-morbidities are an important consideration with respect to translational application of the present findings. Finally, we did not formally assess the relative efficacy of these medications with respect to each other, using network meta-analysis, and this would be an interesting avenue for further exploration.

## Clinical implications and conclusions

This systematic review and meta-analysis of in vivo, experimental studies of myocarditis demonstrates a significant impact of treatment with beta blockers, calcium channel blockers or RAS antagonists in ameliorating necrosis, fibrosis and calcification. Observed heterogeneity was contingent on drug class. Beta blockers and ACE inhibitors were the only agents that were effective for both the clinically common sequalae of necrosis and fibrosis. They also attenuated inflammation together with improving HW/BW and survival. ACE inhibitors were also effective at preventing calcification, although this is an unusual clinical manifestation.

Clinically, the use of CMR has allowed quantification of replacement fibrosis using late gadolinium enhancement. This is a nidus for arrhythmia [84], and is associated with increased risk of MACE, even after adjustment for LV function [8–10]. This association remains in patients with LV ejection fraction ≥ 40%, a range in which prognostic heart failure medication is not indicated [8, 9]. In contrast, the absence of late gadolinium enhancement and normal LV ejection fraction was associated with a very low event rate [8, 9]. There is therefore an unmet need for therapeutic agents that target myocyte necrosis and fibrosis in the context of myocarditis with normal LV ejection fraction at presentation.

To date, this meta-analysis provides the most robust pre-clinical evidence for a role for ACE inhibitors or beta blockers in this setting. However, this enthusiasm is tempered by mixed methodological quality, risk of bias and the age of the literature. There is an urgent need for contemporary, well-performed studies using more advanced techniques and improved current understanding of the immune response to cardiac injury. In addition, more mechanistic studies are needed so we can better target therapy to patients who are most likely to gain benefit.

We advocate clinical studies investigating beta blockers and ACE inhibitors in patients presenting with myocarditis in the absence of LV dysfunction. Such trials should cautiously consider investigating all aetiologies due to the apparent absence of harm in virus-induced myocarditis and starting treatment as early as practicable due to evidence of greater benefit.

## Electronic supplementary material

Below is the link to the electronic supplementary material.

Supplementary material 1 (DOCX 365 kb)

Supplementary material 2 (PDF 343 kb)

Supplementary material 3 (PDF 355 kb)

Supplementary material 4 (PDF 13 kb)

Supplementary material 5 (PDF 262 kb)

Supplementary material 6 (PDF 14 kb)

Supplementary material 7 (PDF 15 kb)

Supplementary material 8 (PDF 12 kb)

Supplementary material 9 (PDF 10 kb)

Supplementary material 10 (PDF 10 kb)

Supplementary material 11 (PDF 10 kb)

Supplementary material 12 (PDF 10 kb)

Supplementary material 13 (PDF 11 kb)

Supplementary material 14 (PDF 11 kb)

Supplementary material 15 (DOC 63 kb)

Supplementary material 16 (DOC 57 kb)

## References

[1] Richardson P, McKenna W, Bristow M, Maisch B, Mautner B, O’Connell J, Olsen E, Thiene G, Goodwin J, Gyarfas I, Martin I, Nordet P (1996). Report of the 1995 World Health Organization/International Society and Federation of Cardiology Task Force on the definition and classification of cardiomyopathies. Circulation.

[2] Caforio AL, Pankuweit S, Arbustini E, Basso C, Gimeno-Blanes J, Felix SB, Fu M, Helio T, Heymans S, Jahns R, Klingel K, Linhart A, Maisch B, McKenna W, Mogensen J, Pinto YM, Ristic A, Schultheiss HP, Seggewiss H, Tavazzi L, Thiene G, Yilmaz A, Charron P, Elliott PM, European Society of Cardiology Working Group on M, Pericardial D (2013). Current state of knowledge on aetiology, diagnosis, management, and therapy of myocarditis: a position statement of the European Society of Cardiology Working Group on Myocardial and Pericardial Diseases. Eur Heart J.

[3] Caforio AL, Calabrese F, Angelini A, Tona F, Vinci A, Bottaro S, Ramondo A, Carturan E, Iliceto S, Thiene G, Daliento L (2007). A prospective study of biopsy-proven myocarditis: prognostic relevance of clinical and aetiopathogenetic features at diagnosis. Eur Heart J.

[4] Felker GM, Hu W, Hare JM, Hruban RH, Baughman KL, Kasper EK (1999). The spectrum of dilated cardiomyopathy. The Johns Hopkins experience with 1278 patients. Medicine (Baltimore).

[5] Kindermann I, Barth C, Mahfoud F, Ukena C, Lenski M, Yilmaz A, Klingel K, Kandolf R, Sechtem U, Cooper LT, Bohm M (2012). Update on myocarditis. J Am Coll Cardiol.

[6] Gulati A, Jabbour A, Ismail TF, Guha K, Khwaja J, Raza S, Morarji K, Brown TD, Ismail NA, Dweck MR, Di Pietro E, Roughton M, Wage R, Daryani Y, O’Hanlon R, Sheppard MN, Alpendurada F, Lyon AR, Cook SA, Cowie MR, Assomull RG, Pennell DJ, Prasad SK (2013). Association of fibrosis with mortality and sudden cardiac death in patients with nonischemic dilated cardiomyopathy. JAMA.

[7] Halliday BP, Gulati A, Ali A, Guha K, Newsome S, Arzanauskaite M, Vassiliou VS, Lota A, Izgi C, Tayal U, Khalique Z, Stirrat C, Auger D, Pareek N, Ismail TF, Rosen SD, Vazir A, Alpendurada F, Gregson J, Frenneaux MP, Cowie MR, Cleland JGF, Cook SA, Pennell DJ, Prasad SK (2017). Association between Midwall Late Gadolinium enhancement and sudden cardiac death in patients with dilated cardiomyopathy and mild and moderate left ventricular systolic dysfunction. Circulation.

[8] Aquaro GD, Perfetti M, Camastra G, Monti L, Dellegrottaglie S, Moro C, Pepe A, Todiere G, Lanzillo C, Scatteia A, Di Roma M, Pontone G, Perazzolo Marra M, Barison A, Di Bella G, Cardiac Magnetic Resonance Working Group of the Italian Society of C (2017). Cardiac MR with late gadolinium enhancement in acute myocarditis with preserved systolic function: ITAMY Study. J Am Coll Cardiol.

[9] Grani C, Eichhorn C, Biere L, Murthy VL, Agarwal V, Kaneko K, Cuddy S, Aghayev A, Steigner M, Blankstein R, Jerosch-Herold M, Kwong RY (2017). Prognostic value of cardiac magnetic resonance tissue characterization in risk stratifying patients with suspected myocarditis. J Am Coll Cardiol.

[10] Halliday BP, Baksi AJ, Gulati A, Ali A, Newsome S, Izgi C, Arzanauskaite M, Lota A, Tayal U, Vassiliou VS, Gregson J, Alpendurada F, Frenneaux MP, Cook SA, Cleland JGF, Pennell DJ, Prasad SK (2018). Outcome in dilated cardiomyopathy related to the extent, location, and pattern of Late Gadolinium enhancement. JACC Cardiovasc Imaging.

[11] Adler Y, Charron P, Imazio M, Badano L, Baron-Esquivias G, Bogaert J, Brucato A, Gueret P, Klingel K, Lionis C, Maisch B, Mayosi B, Pavie A, Ristic AD, Sabate Tenas M, Seferovic P, Swedberg K, Tomkowski W, Group ESCSD (2015). 2015 ESC guidelines for the diagnosis and management of pericardial diseases: the task force for the diagnosis and management of pericardial diseases of the European Society of Cardiology (ESC) endorsed by: the European Association for Cardio-Thoracic Surgery (EACTS). Eur Heart J.

[12] Liberati A, Altman DG, Tetzlaff J, Mulrow C, Gotzsche PC, Ioannidis JP, Clarke M, Devereaux PJ, Kleijnen J, Moher D (2009). The PRISMA statement for reporting systematic reviews and meta-analyses of studies that evaluate healthcare interventions: explanation and elaboration. BMJ.

[13] Flores-Mir C, Major MP, Major PW (2006). Search and selection methodology of systematic reviews in orthodontics (2000–2004). Am J Orthodont Dentofac Orthop.

[14] Major MP, Major PW, Flores-Mir C (2007). Benchmarking of reported search and selection methods of systematic reviews by dental speciality. Evid Based Dentist.

[15] Major MP, Major PW, Flores-Mir C (2006). An evaluation of search and selection methods used in dental systematic reviews published in English. J Am Dent Assoc.

[16] Higgins JPT, Green S, eds. Cochrane handbook for systematic reviews of interventions version 510: the Cochrane Collaboration; 2011.

[17] Atteya M, Mohamed RA, Ahmed AM, Abdel-Baky NA, Alfayez MA, Almalke HD, El Fouhil AF (2017). Lisinopril has a cardio-protective effect on experimental acute autoimmune myocarditis in rats. Histol Histopathol.

[18] Liu H, Li W, Gu W, Kong Y, Yang N, Chen L (2010). Immunoregulatory effects of carvedilol on rat experimental autoimmune myocarditis. Scand J Immunol.

[19] Nimata M, Kishimoto C, Yuan Z, Shioji K (2004). Beneficial effects of olmesartan, a novel angiotensin II receptor type 1 antagonist, upon acute autoimmune myocarditis. Mol Cell Biochem.

[20] Nishii M, Inomata T, Niwano H, Takehana H, Takeuchi I, Nakano H, Shinagawa H, Naruke T, Koitabashi T, Nakahata J, Izumi T (2006). Beta2-Adrenergic agonists suppress rat autoimmune myocarditis: potential role of beta2-adrenergic stimulants as new therapeutic agents for myocarditis. Circulation.

[21] Ogawa T, Veinot JP, Kuroski de Bold ML, Georgalis T, de Bold AJ (2008). Angiotensin II receptor antagonism reverts the selective cardiac BNP upregulation and secretion observed in myocarditis. Am J Physiol Heart Circ Physiol.

[22] Seko Y (2006). Effect of the angiotensin II receptor blocker olmesartan on the development of murine acute myocarditis caused by coxsackievirus B3. Clin Sci (Lond).

[23] Skrzypiec-Spring M, Haczkiewicz K, Sapa A, Piasecki T, Kwiatkowska J, Ceremuga I, Wozniak M, Biczysko W, Kobierzycki C, Dziegiel P, Podhorska-Okolow M, Szelag A (2018). Carvedilol inhibits matrix metalloproteinase-2 activation in experimental autoimmune myocarditis: possibilities of cardioprotective application. J Cardiovasc Pharmacol Ther.

[24] Wang D, Chen Y, Jiang J, Zhou A, Pan L, Chen Q, Qian Y, Chu M, Chen C (2014). Carvedilol has stronger anti-inflammation and anti-virus effects than metoprolol in murine model with coxsackievirus B3-induced viral myocarditis. Gene.

[25] Wang JF, Min JY, Hampton TG, Amende I, Yan X, Malek S, Abelmann WH, Green AI, Zeind J, Morgan JP (2008). Clozapine-induced myocarditis: role of catecholamines in a murine model. Eur J Pharmacol.

[26] Yuan Z, Kishimoto C, Shioji K (2003). Beneficial effects of low-dose benidipine in acute autoimmune myocarditis: suppressive effects on inflammatory cytokines and inducible nitric oxide synthase. Circ J.

[27] Yuan Z, Kishimoto C, Shioji K, Nakamura H, Yodoi J, Sasayama S (2003). Temocapril treatment ameliorates autoimmune myocarditis associated with enhanced cardiomyocyte thioredoxin expression. Mol Cell Biochem.

[28] Yuan Z, Nimata M, Okabe TA, Shioji K, Hasegawa K, Kita T, Kishimoto C (2005). Olmesartan, a novel AT1 antagonist, suppresses cytotoxic myocardial injury in autoimmune heart failure. Am J Physiol Heart Circ Physiol.

[29] Yuan Z, Shioji K, Kihara Y, Takenaka H, Onozawa Y, Kishimoto C (2004). Cardioprotective effects of carvedilol on acute autoimmune myocarditis: anti-inflammatory effects associated with antioxidant property. Am J Physiol Heart Circ Physiol.

[30] Zhang YY, Li JN, Xia HH, Zhang SL, Zhong J, Wu YY, Miao SK, Zhou LM (2013). Protective effects of losartan in mice with chronic viral myocarditis induced by coxsackievirus B3. Life Sci.

[31] Araki M, Kanda T, Imai S, Suzuki T, Murata K, Kobayashi I (1995). Comparative effects of losartan, captopril, and enalapril on murine acute myocarditis due to encephalomyocarditis virus. J Cardiovasc Pharmacol.

[32] Chen XJ, Bian ZP, Lu S, Xu JD, Gu CR, Yang D, Zhang JN (2006). Cardiac protective effect of Astragalus on viral myocarditis mice: comparison with Perindopril. Am J Chin Med.

[33] Rezkalla S, Kloner RA, Khatib G, Khatib R (1990). Beneficial effects of captopril in acute coxsackievirus B3 murine myocarditis. Circulation.

[34] Rezkalla S, Kloner RA, Khatib G, Khatib R (1990). Effect of delayed captopril therapy on left ventricular mass and myonecrosis during acute coxsackievirus murine myocarditis. Am Heart J.

[35] Rezkalla S, Kloner RA, Khatib G, Smith FE, Khatib R (1988). Effect of metoprolol in acute coxsackievirus B3 murine myocarditis. J Am Coll Cardiol.

[36] Suzuki H, Matsumori A, Matoba Y, Kyu BS, Tanaka A, Fujita J, Sasayama S (1993). Enhanced expression of superoxide dismutase messenger RNA in viral myocarditis. An SH-dependent reduction of its expression and myocardial injury. J Clin Invest.

[37] Hooijmans CR, Rovers MM, de Vries RB, Leenaars M, Ritskes-Hoitinga M, Langendam MW (2014). SYRCLE’s risk of bias tool for animal studies. BMC Med Res Methodol.

[38] Macleod MR, O’Collins T, Howells DW, Donnan GA (2004). Pooling of animal experimental data reveals influence of study design and publication bias. Stroke J Cereb Circ.

[39] Vesterinen HM, Sena ES, Egan KJ, Hirst TC, Churolov L, Currie GL, Antonic A, Howells DW, Macleod MR (2014). Meta-analysis of data from animal studies: a practical guide. J Neurosci Methods.

[40] DerSimonian R, Laird N (1986). Meta-analysis in clinical trials. Control Clin Trials.

[41] Hooijmans CR, IntHout J, Ritskes-Hoitinga M, Rovers MM (2014). Meta-analyses of animal studies: an introduction of a valuable instrument to further improve healthcare. ILAR J Natl Res Council Inst Lab Anim Resour.

[42] Duval S, Tweedie R (2000). Trim and fill: a simple funnel-plot-based method of testing and adjusting for publication bias in meta-analysis. Biometrics.

[43] Liu W, Shimada M, Xiao J, Hu D, Matsumori A (2009). Nifedipine inhibits the activation of inflammatory and immune reactions in viral myocarditis. Life Sci.

[44] Kawai C (1999). From myocarditis to cardiomyopathy: mechanisms of inflammation and cell death: learning from the past for the future. Circulation.

[45] Kearney MT, Cotton JM, Richardson PJ, Shah AM (2001). Viral myocarditis and dilated cardiomyopathy: mechanisms, manifestations, and management. Postgrad Med J.

[46] Zannad F, Gattis Stough W, Rossignol P, Bauersachs J, McMurray JJ, Swedberg K, Struthers AD, Voors AA, Ruilope LM, Bakris GL, O’Connor CM, Gheorghiade M, Mentz RJ, Cohen-Solal A, Maggioni AP, Beygui F, Filippatos GS, Massy ZA, Pathak A, Pina IL, Sabbah HN, Sica DA, Tavazzi L, Pitt B (2012). Mineralocorticoid receptor antagonists for heart failure with reduced ejection fraction: integrating evidence into clinical practice. Eur Heart J.

[47] Johar S, Cave AC, Narayanapanicker A, Grieve DJ, Shah AM (2006). Aldosterone mediates angiotensin II-induced interstitial cardiac fibrosis via a Nox2-containing NADPH oxidase. FASEB J.

[48] Brilla CG (2000). Aldosterone and myocardial fibrosis in heart failure. Herz.

[49] Fraccarollo D, Galuppo P, Bauersachs J (2004). Mineralocorticoid receptor antagonism and cardiac remodeling in ischemic heart failure. Curr Med Chem Cardiovasc Hematol Agents.

[50] Hayashi M, Tsutamoto T, Wada A, Tsutsui T, Ishii C, Ohno K, Fujii M, Taniguchi A, Hamatani T, Nozato Y, Kataoka K, Morigami N, Ohnishi M, Kinoshita M, Horie M (2003). Immediate administration of mineralocorticoid receptor antagonist spironolactone prevents post-infarct left ventricular remodeling associated with suppression of a marker of myocardial collagen synthesis in patients with first anterior acute myocardial infarction. Circulation.

[51] Iraqi W, Rossignol P, Angioi M, Fay R, Nuee J, Ketelslegers JM, Vincent J, Pitt B, Zannad F (2009). Extracellular cardiac matrix biomarkers in patients with acute myocardial infarction complicated by left ventricular dysfunction and heart failure: insights from the Eplerenone Post-Acute Myocardial Infarction Heart Failure Efficacy and Survival Study (EPHESUS) study. Circulation.

[52] Suzuki G, Morita H, Mishima T, Sharov VG, Todor A, Tanhehco EJ, Rudolph AE, McMahon EG, Goldstein S, Sabbah HN (2002). Effects of long-term monotherapy with eplerenone, a novel aldosterone blocker, on progression of left ventricular dysfunction and remodeling in dogs with heart failure. Circulation.

[53] Zannad F, Alla F, Dousset B, Perez A, Pitt B (2000). Limitation of excessive extracellular matrix turnover may contribute to survival benefit of spironolactone therapy in patients with congestive heart failure: insights from the randomized aldactone evaluation study (RALES). Rales Investigators. Circulation.

[54] Sena ES, van der Worp HB, Bath PM, Howells DW, Macleod MR (2010). Publication bias in reports of animal stroke studies leads to major overstatement of efficacy. PLoS Biol.

[55] van der Worp HB, Howells DW, Sena ES, Porritt MJ, Rewell S, O’Collins V, Macleod MR (2010). Can animal models of disease reliably inform human studies?. PLoS Med.

[56] van der Worp HB, Macleod MR (2011). Preclinical studies of human disease: time to take methodological quality seriously. J Mol Cell Cardiol.

[57] Hirst JA, Howick J, Aronson JK, Roberts N, Perera R, Koshiaris C, Heneghan C (2014). The need for randomization in animal trials: an overview of systematic reviews. PloS One.

[58] Wever KE, Hooijmans CR, Riksen NP, Sterenborg TB, Sena ES, Ritskes-Hoitinga M, Warle MC (2015). Determinants of the efficacy of cardiac ischemic preconditioning: a systematic review and meta-analysis of animal studies. PLoS One.

[59] Baba T, Kanda T, Kobayashi I (2000). Reduction of cardiac endothelin-1 by angiotensin II type 1 receptor antagonist in viral myocarditis of mice. Life Sci.

[60] Teng G, Svystonyuk D, Mewhort HE, Turnbull JD, Belke DD, Duff HJ, Fedak PW (2015). Tetrandrine reverses human cardiac myofibroblast activation and myocardial fibrosis. Am J Physiol Heart Circ Physiol.

[61] Wahed MI, Watanabe K, Ma M, Nakazawa M, Takahashi T, Hasegawa G, Naito M, Yamamoto T, Kodama M, Aizawa Y (2004). Effects of pranidipine, a novel calcium channel antagonist, on the progression of left ventricular dysfunction and remodeling in rats with heart failure. Pharmacology.

[62] Veeraveedu PT, Watanabe K, Ma M, Gurusamy N, Palaniyandi SS, Wen J, Prakash P, Wahed MI, Kamal FA, Mito S, Kunisaki M, Kodama M, Aizawa Y (2006). Comparative effects of pranidipine with amlodipine in rats with heart failure. Pharmacology.

[63] Packer M, O’Connor CM, Ghali JK, Pressler ML, Carson PE, Belkin RN, Miller AB, Neuberg GW, Frid D, Wertheimer JH, Cropp AB, DeMets DL (1996). Effect of amlodipine on morbidity and mortality in severe chronic heart failure. Prospective Randomized Amlodipine Survival Evaluation Study Group. N Engl J Med.

[64] Goldstein RE, Boccuzzi SJ, Cruess D, Nattel S (1991). Diltiazem increases late-onset congestive heart failure in postinfarction patients with early reduction in ejection fraction. The Adverse Experience Committee; and the Multicenter Diltiazem Postinfarction Research Group. Circulation.

[65] Yoshiyama M, Takeuchi K, Omura T, Izutani S, Nakamura Y, Akioka K, Kim S, Iwao H, Yoshikawa J (1999). Effect of diltiazem on cardiac remodeling in rats assessed by Doppler echocardiography and mRNA expression. Cardiovasc Drugs Ther.

[66] Mason JW, O’Connell JB, Herskowitz A, Rose NR, McManus BM, Billingham ME, Moon TE (1995). A clinical trial of immunosuppressive therapy for myocarditis. The Myocarditis Treatment Trial Investigators. N Engl J Med.

[67] Gluck B, Dahlke K, Zell R, Krumbholz A, Decker M, Lehmann J, Wutzler P (2010). Cardioprotective effect of NO-metoprolol in murine coxsackievirus B3-induced myocarditis. J Med Virol.

[68] Nishio R, Shioi T, Sasayama S, Matsumori A (2003). Carvedilol increases the production of interleukin-12 and interferon-gamma and improves the survival of mice infected with the encephalomyocarditis virus. J Am Coll Cardiol.

[69] Yue-Chun L, Li-Sha G, Jiang-Hua R, Peng-Lin Y, Jia-Feng L, Ji-Fei T, Peng C, Zhan-Qiu Y (2008). Protective effects of carvedilol in murine model with the coxsackievirus B3-induced viral myocarditis. J Cardiovasc Pharmacol.

[70] Yue-Chun L, Teng Z, Na-Dan Z, Li-Sha G, Qin L, Xue-Qiang G, Jia-Feng L (2012). Comparison of effects of ivabradine versus carvedilol in murine model with the Coxsackievirus B3-induced viral myocarditis. PLoS One.

[71] Stallion A, Rafferty JF, Warner BW, Ziegler MM, Ryckman FC (1994). Myocardial calcification: a predictor of poor outcome for myocarditis treated with extracorporeal life support. J Pediatr Surg.

[72] ClinicalTrials.gov. https://www.clinicaltrials.gov/ct2/show/NCT02740179?term=miracle+hiv&rank=1. Accessed 23 Apr 2019

[73] Saegusa S, Fei Y, Takahashi T, Sumino H, Moriya J, Kawaura K, Yamakawa J, Itoh T, Morimoto S, Nakahashi T, Iwai K, Matsumoto M, Kanda T (2007). Oral administration of candesartan improves the survival of mice with viral myocarditis through modification of cardiac adiponectin expression. Cardiovasc Drugs Ther.

[74] Wang WZ, Matsumori A, Yamada T, Shioi T, Okada I, Matsui S, Sato Y, Suzuki H, Shiota K, Sasayama S (1997). Beneficial effects of amlodipine in a murine model of congestive heart failure induced by viral myocarditis. A possible mechanism through inhibition of nitric oxide production. Circulation.

[75] Wang JF, Meissner A, Malek S, Chen Y, Ke Q, Zhang J, Chu V, Hampton TG, Crumpacker CS, Abelmann WH, Amende I, Morgan JP (2005). Propranolol ameliorates and epinephrine exacerbates progression of acute and chronic viral myocarditis. Am J Physiol Heart Circ Physiol.

[76] Egger M, Davey Smith G, Schneider M, Minder C (1997). Bias in meta-analysis detected by a simple, graphical test. BMJ.

[77] Reeves B, Deeks J, Higgins J, Wells G, Higgins J, Green S (2008). Including non-randomized studies. Cochrane handbook for systematic reviews of interventions.

[78] Li YC, Ge LS, Yang PL, Tang JF, Lin JF, Chen P, Guan XQ (2010). Carvedilol treatment ameliorates acute coxsackievirus B3-induced myocarditis associated with oxidative stress reduction. Eur J Pharmacol.

[79] Sukumaran V, Veeraveedu PT, Gurusamy N, Yamaguchi K, Lakshmanan AP, Ma M, Suzuki K, Kodama M, Watanabe K (2011). Cardioprotective effects of telmisartan against heart failure in rats induced by experimental autoimmune myocarditis through the modulation of angiotensin-converting enzyme-2/angiotensin 1-7/mas receptor axis. Int J Biol Sci.

[80] Tachikawa H, Kodama M, Hui L, Yoshida T, Hayashi M, Abe S, Kashimura T, Kato K, Hanawa H, Watanabe K, Nakazawa M, Aizawa Y (2003). Angiotensin II type 1 receptor blocker, valsartan, prevented cardiac fibrosis in rat cardiomyopathy after autoimmune myocarditis. J Cardiovasc Pharmacol.

[81] Takamura C, Suzuki J, Ogawa M, Watanabe R, Tada Y, Maejima Y, Akazawa H, Komuro I, Isobe M (2016). Suppression of murine autoimmune myocarditis achieved with direct renin inhibition. J Cardiol.

[82] Wahed MI, Watanabe K, Ma M, Yamaguchi K, Takahashi T, Tachikawa H, Kodama M, Aizawa Y (2005). Effects of eplerenone, a selective aldosterone blocker, on the progression of left ventricular dysfunction and remodeling in rats with dilated cardiomyopathy. Pharmacology.

[83] Bromage DI, Pickard JM, Rossello X, Ziff OJ, Burke N, Yellon DM, Davidson SM (2017). Remote ischaemic conditioning reduces infarct size in animal in vivo models of ischaemia-reperfusion injury: a systematic review and meta-analysis. Cardiovasc Res.

[84] Muser D, Santangeli P, Selvanayagam JB, Nucifora G (2019). Role of cardiac magnetic resonance imaging in patients with idiopathic ventricular arrhythmias. Curr Cardiol Rev.

