# Supplementary material for: Therapies to limit myocardial injury in animal models of myocarditis: a systematic review and meta-analysis
Source: Basic Res Cardiol. 2019 Oct 31;114(6):48. doi: 10.1007/s00395-019-0754-x (PMC6823299; doi:10.1007/s00395-019-0754-x)
Supplement: Supplementary file 1 — Supplementary material 1 (DOCX 365 kb) [file 395_2019_754_MOESM1_ESM.docx]

SUPPLEMENTARY MATERIAL

PICOS statement

*In vivo* animal studies were included and were eligible if they investigated the effect of beta blockers, calcium channel blockers and/or medications that manipulate the renin-angiotensin system (including angiotensin converting enzyme inhibitor, angiotensin receptor blockers and aldosterone antagonists) vs. control (sham treatment) on histological parameters of scar formation and remodelling, including myofibrilar diameter, necrosis, calcification or fibrosis, in any mammalian species with experimental myocarditis (however induced), regardless of study design (**Table S1**).

| Category | Data item |
| --- | --- |
| Study details | First author, senior author, date, Journal |
| Populations/subjects (P) | Mammalian species with experimental myocarditis (however induced) |
| Interventions (I) | Beta blockers, calcium channel blockers, medications that manipulate the renin-angiotensin system (including angiotensin converting enzyme inhibitor, angiotensin receptor blockers and aldosterone antagonists) |
| Outcomes (O) | Histological parameters of scar formation and remodelling, including myofibrilar diameter, necrosis, calcification, fibrosis |
| Study design (S) | Any |

Table S1. Data items, developed using the PICOS approach

Search strategy

1. exp myocarditis/
2. (myocarditis or encephalomyocarditis or inflammatory cardiomyopathy).mp.
3. 1 or 2
4. exp renin angiotensin aldosterone system/
5. renin angiotensin system.mp.
6. 4 or 5
7. exp angiotensin-converting enzyme inhibitors/
8. Angiotensin-Converting Enzyme Inhibitor$.mp.
9. (captopril or co-zidocapt or enalapril or fosinopril sodium or imidapril hydrochloride or lisinopril or moexipril hydrochloride or perindopril or quinapril or ramipril or trandolapril).mp.
10. 7 or 8 or 9
11. exp Angiotensin Receptor Antagonist/ or exp sartan derivative/
12. (Angiotensin Receptor Antagonist$ or Angiotensin Receptor Blocker or Sartan).mp.
13. (amlodipine or azilsartan medoxomil or andesartan cilexetil or eprosartan or irbesartan or losartan or olmesartan or telmisartan or valsartan).mp.
14. 11 or 12 or 13
15. exp renin inhibitor/ or exp renin/
16. (Renin-Angiotensin System or Renin).mp.
17. aliskiren.mp.
18. 15 or 16 or 17
19. exp mineralocorticoid antagonist/
20. (mineralcorticoid receptor antagonist$ or aldosterone antagonist$ or antimineralocorticoid).mp.
21. (co-flumactone or eplerenone or spironolactone).mp.
22. 19 or 20 or 21
23. exp beta adrenergic receptor blocking agent/
24. beta block$.mp.
25. exp atenolol/ or exp bisoprolol/ or exp metoprolol/ or exp nebivolol/ or exp nadolol/ or exp propranolol/
26. (acebutolol or atenolol or betaxolol or bimatoprost or bisoprolol fumarate or brimonidine or brinzolamide or carteolol hydrochloride or carvedilol or celiprolol hydrochloride or co-tenidone or dorzolamide or esmolol hydrochloride or labetalol hydrochloride or latanoprost or levobunolol hydrochloride or metoprolol or mirabegron or nadolol or nebivolol or oxprenolol hydrochloride or pindolol or propranolol hydrochloride or sotalol hydrochloride or tafluprost or tartrate or timolol or travoprost).mp.
27. 23 or 24 or 25 or 26
28. exp calcium channel blocking agent/
29. calcium channel antagonist$.mp.
30. exp amlodipine/ or exp diltiazem/ or exp felodipine/ or exp isradipine/ or exp nicardipine/ or exp nifedipine/ or exp nisoldipine/ or exp verapamil/
31. (amlodipine or atenolol or clevidipine or diltiazem hydrochloride or felodipine or isradipine or lacidipine or lercanidipine hydrochloride or nicardipine hydrochloride or nifedipine or nimodipine or olmesartan or ramipril or verapamil hydrochloride).mp.
32. 28 or 29 or 30 or 31
33. 6 or 10 or 14 or 18 or 22 or 27 or 32
34. 3 and 33
35. 34 not exp "Review"/
36. Limit 35 to animal
37. limit 36 to english language

Differences in the search strategy between Medline and Embase are described in **Table S2**.

| Search term | Database | |
| --- | --- | --- |
|  | Embase | Medline |
| Angiotensin-Converting Enzyme Inhibitors | No available Mesh term | Angiotensin-Converting Enzyme Inhibitors/ (MeSH term) |
| Angiotensin receptor antagonist | Angiotensin receptor antagonist/ (MeSH term) | Angiotensin receptor antagonists/ (MeSH term) |
| Sartans | Sartan derivative/ (MeSH term) | Sartan/ (MeSH term) |
| Renin | Renin angiotensin aldosterone system/ or Renin inhibitor/ or Renin/ | Renin-Angiotensin System/ or Renin/ (MeSH term) |
| Mineralocorticoid Receptor Antagonist | Mineralocorticoid Antagonist/ (MeSH term) | Mineralocorticoid Receptor Antagonists/ (MeSH term) |
| Adrenergic beta-Antagonists | beta adrenergic receptor blocking agent/ (MeSH term) | Adrenergic beta-Antagonists/ (MeSH term) |
| Calcium Channel blockers | Calcium channel blocking agent/ (MeSH term) | Calcium Channel Blockers/ (MeSH term) |

Table S2. Differences in search strategy between databases

Data collection process

We developed a data extraction sheet based on the Cochrane Consumers and Communications Review Group’s data extraction template.[^1^](#_ENREF_1) Following data extraction, searches will be conducted based on first and senior author name, sample size and size of outcome to identify double counting. These parameters have been chosen due to expected high completeness and based on recommendations in the PRISMA statement.[^2^](#_ENREF_2) If multiple reports for the same study are identified they will be compared for logical inconsistencies, which will subsequently be accounted for by contacting the report author by e-mail.

Data items

With respect to the animal model, we extracted details on the species, sex, strain, age, and the method, dose and frequency of myocarditis induction. With respect to the intervention we extracted details of the drug used, together with its dose, route of administration, start date and length of treatment. We made the following assumptions:

1. With respect to outcome recording, most studies used either a 1-4 scale or percentage, as follows: grade 1, lesions involving <25% of the myocardium; grade 2, lesions involving 25-50%; grade 3, lesions involving 50-75%; and grade 4, lesions involving 75-100%.[^3^](#_ENREF_3) Others defined a similar scoring system of 0-4, whereby 1, 2, 3 and 4 represent 25%, 50%, 75% and 100% involvement of the histological section, respectively.[^4^](#_ENREF_4) We assumed that these scales were the same. Studies and outcomes that did not use this scoring system,[^5^](#_ENREF_5) or used a composite score with no indication of scores for individual outcomes,[^6^](#_ENREF_6) were excluded if the information was not available from the study authors.
2. Three studies, the timing of treatment was not specified and we assumed it was started concurrently with myocarditis induction.[^7-9^](#_ENREF_7)
3. In one study, two histopathological stains were used. We extracted data acquired using H&E staining to be consistent with other studies.[^5^](#_ENREF_5)
4. One study described ‘mineralisation’. We assumed this to be synonymous with calcification.[^10^](#_ENREF_10)
5. One study provided a treatment window of 42-46 d. We assumed a mean treatment duration of 44 d.[^11^](#_ENREF_11) In addition, rats were only treated for 6 d per week and we assumed this to be equivalent to treatment for 7 d per week used in other studies.
6. We assumed that animals were sacrificed for histological assessment concurrently with the end of treatment, unless explicitly stated.[^10^](#_ENREF_10)^,^[^12^](#_ENREF_12)
7. One study did not specify histological stain, but 7 of 8 similar studies from the same group used Azan-Mallory, so we have assumed this study to be the same.[^8^](#_ENREF_8)

Statistical methods

The following data processing was performed during analysis:

1. Where HW and BW were given separately, approximations for mean and variance of a ratio were calculated using a Taylor series expansion method. We used Eq (20) and assumed that the covariance between R and S was 0.[^5^](#_ENREF_5)^,^[^13^](#_ENREF_13)^,^[^14^](#_ENREF_14)
2. When it was not clear if given variances were SD or SEM, this was derived using descriptive statistics where possible.[^3^](#_ENREF_3) In one study, where the measure of variance was not stated or derivable, we assumed it to be SD.[^15^](#_ENREF_15)
3. Where date were presented as median and IQR,[^16^](#_ENREF_16)^,^[^17^](#_ENREF_17) the sample mean and SD were estimated using the methods described by Wan *et al.*[^18^](#_ENREF_18)
4. For the survival endpoint, we used 0.5 in place of 0 to avoid a computational error by dividing by a zero count (see: https://handbook-5-1.cochrane.org/chapter_16/16_9_2_studies_with_zero_cell_counts.htm).[^19^](#_ENREF_19) As studies with non-positive values for standard deviation get no weight in meta-analysis, where standard deviation was 0 we corrected this to 0.01.[^3^](#_ENREF_3)^,^[^10^](#_ENREF_10)^,^[^20^](#_ENREF_20) We performed a sensitivity analysis and inclusion/exclusion of these studies had no effect on results.

Results

Our search returned 347 records, including 124 duplicate reports (consisting of reports returned by both Medline and Embase). 223 reports underwent title and abstract screening, which resulted in 132 exclusions. The remaining 91 reports were retrieved for detailed full text evaluation. 39 articles were excluded, 29 because they failed to meet the inclusion criteria, 4 were abstracts, 3 were reviews, 1 was a letter and 1 that appeared to repeat data reported elsewhere. Of the remaining 53 reports 1 was missing data on one or more important experimental variables that we were unable to retrieve by contacting the study authors.[^21^](#_ENREF_21)

Qualitative data extraction table

| **First Author** | **Age/weight** | **Dose** | **Frequency** | **Drug** | **Dose (mg/kg/d)** | **Fibrosis stain** | **Necrosis stain** | **Calcification stain** | **Inflammation stain** |
| --- | --- | --- | --- | --- | --- | --- | --- | --- | --- |
| **Abdel-Wahab 2014 - a** | 200-250g | 25mg/kg | Daily for duration | Captopril | 5 | HE |  |  |  |
| **Abdel-Wahab 2014- b** | 200-250g | 25mg/kg | Daily for duration | Captopril | 10 | HE |  |  |  |
| **Araki 1995 - a** | 8w | 500 pfu | Once At Start | Captopril | 7.5 |  | HE | HE | HE |
| **Araki 1995 - b** | 8w | 500 pfu | Once At Start | Enalapril | 1 |  | HE | HE | HE |
| **Araki 1995 - c** | 8w | 500 pfu | Once At Start | Losartan | 1.2 |  | HE | HE | HE |
| **Araki 1995 - d** | 8w | 500 pfu | Once At Start | Losartan | 12 |  | HE | HE | HE |
| **Araki 1995 - e** | 8w | 500 pfu | Once At Start | Losartan | 60 |  | HE | HE | HE |
| **Baba 2000 - a** | 8w | 500 pfu | Once At Start | L-158,809 | 0.12 |  | HE |  | HE |
| **Baba 2000 - b** | 8w | 500 pfu | Once At Start | L-158,809 | 1.2 |  | HE |  | HE |
| **Baba 2000 - c** | 8w | 500 pfu | Once At Start | L-158,809 | 6 |  | HE |  | HE |
| **Baba 2000 - d** | 8w | 500 pfu | Once At Start | Enalapril | 1 |  | HE |  | HE |
| **Baba 2000 - e** | 8w | 500 pfu | Once At Start | Captopril | 7.5 |  | HE |  | HE |
| **Bahk 2007 - a** | 4-6w | 300μg | Once At Start | Losartan | 250 mg/L in drinking water | MT |  |  | HE |
| **Bahk 2007 - b** | 4-6w | 300μg | Once At Start | Captopril | 75mg/L in drinking water | MT |  |  | HE |
| **Chen 2006** | 3w 12-15g | N/A | Once At Start | Perindopril | 0.44mcg/kg/day |  | HE |  | HE |
| **Gluck 2010 - a** | 7-9w | 10000 pfu | Once At Start | Metoprolol | 0.67 | HE |  |  | HE |
| **Gluck 2010 - b** | 7-9w | 10000 pfu | Once At Start | Metoprolol | 0.67 | HE |  |  | HE |
| **Godsel 2003** | 6-8w | 300μg | Day 1 and 8 | Captopril | 25mg/kg | MT |  |  | HE |
| **Guo 2009** | 4w 15-20g | 20000 pfu | Day 1, 15, 29 | Captopril | 100 | HE |  |  |  |
| **Juan 2003 - a** | 9w | Not stated | Once At Start | Quinapril | 0.2 | AM |  |  |  |
| **Juan 2003 - b** | 9w | Not stated | Once At Start | Quinapril | 2 | AM |  |  |  |
| **Juan 2003 - c** | 9w | Not stated | Once At Start | Quinapril | 20 | AM |  |  |  |
| **Kanda 1995 - a** | 8w | 500pfu | Once At Start | Losartan | 1.2 | HE | HE |  |  |
| **Kanda 1995 - b** | 8w | 500pfu | Once At Start | Losartan | 12 | HE | HE |  |  |
| **Kanda 1995 - c** | 8w | 500pfu | Once At Start | Losartan | 60 | HE | HE |  |  |
| **Kanda 1995 - d** | 8w | 500pfu | Once At Start | Captopril | 7.5 | HE | HE |  |  |
| **Kanda 1993 - a** | 8w | 500pfu | Once At Start | Metoprolol | 1 | HE | HE | HE |  |
| **Kanda 1993 - b** | 8w | 500pfu | Once At Start | Metoprolol | 10 | HE | HE | HE |  |
| **Kanda 1993 - c** | 8w | 500pfu | Once At Start | Captopril | 1.2 | HE | HE | HE |  |
| **Kanda 1993 - d** | 8w | 500pfu | Once At Start | Captopril | 12 | HE | HE | HE |  |
| **Kashimura 2003 - a** | 10w | 20000 pfu | Once At Start | Imidapril | 0.5 | AM |  |  |  |
| **Kashimura 2003 - b** | 10w | 20000 pfu | Once At Start | Imidapril | 2 | AM |  |  |  |
| **Kashimura 2003 - c** | 10w | 20000 pfu | Once At Start | TA-606 | 2 | AM |  |  |  |
| **Kashimura 2003 - d** | 10w | 20000 pfu | Once At Start | TA-606 | 6 | AM |  |  |  |
| **Li 2010 - a** | 8w | 1,000,000pfu | Once At Start | Carvedilol | 10 |  | HE |  | HE |
| **Li 2010 - b** | 8w | 1,000,000pfu | Once At Start | Metoprolol | 30 |  | HE |  | HE |
| **Li 2013 - a** | 4w | 1,000,000pfu | Once At Start | Carvedilol | 10 |  | HE |  | HE |
| **Li 2013 - b** | 4w | 1,000,000pfu | Once At Start | Carvedilol | 10 | MT | HE |  | HE |
| **Li-Sha 2013 - a** | 4w | 1,000,000pfu | Once At Start | Carvedilol | 10 |  | HE |  |  |
| **Li-Sha 2013 - b** | 4w | 1,000,000pfu | Once At Start | Carvedilol | 10 | MT | HE |  |  |
| **Liu 2009** | 4w | 2pfu | Once At Start | Nifedipine | Fed chow c̅ 0.01% drug |  | MT |  |  |
| **Ma 2001** | 9w | 2mg | Once At Start | Quinapril | 20 | AM |  |  |  |
| **Nishio 2003 - a** | 4w | 10pfu | Once At Start | Carvedilol | 3 |  | HE |  | HE |
| **Nishio 2003 - b** | 4w | 10pfu | Once At Start | Carvedilol | 10 |  | HE |  | HE |
| **Nishio 2003 - c** | 4w | 10pfu | Once At Start | Carvedilol | 10 |  | HE |  | HE |
| **Nishio 2003 - d** | 4w | 10pfu | Once At Start | Metoprolol | 30 |  | HE |  | HE |
| **Nishio 2003 - e** | 4w | 10pfu | Once At Start | Propranolol | 30 |  | HE |  | HE |
| **Reyes 1988 - a** | 3w | Dose not stated | Once At Start | Captopril | 300! | HE |  |  |  |
| **Reyes 1988 - b** | 3w | Dose not stated | Once At Start | Captopril | 300! | HE |  |  |  |
| **Rezkalla 1988 - a** | 3w | Dose not stated | Once At Start | Metoprolol | 32.5 |  | HE | HE | HE |
| **Rezkalla 1988 - b** | 3w | Dose not stated | Once At Start | Metoprolol | 32.5 |  | HE | HE | HE |
| **Rezkalla 1988 - c** | 3w | Dose not stated | Once At Start | Metoprolol | 32.5 |  | HE | HE | HE |
| **Rezkalla 1988 - d** | 3w | Dose not stated | Once At Start | Metoprolol | 32.5 |  | HE | HE | HE |
| **Rezkalla 1990(1)** | 3w | Dose not stated | Once At Start | Captopril | 100 |  | HE | HE | HE |
| **Rezkalla 1990(2) - a** | 3w | Dose not stated | Once At Start | Captopril | 100 |  | HE | HE | HE |
| **Rezkalla 1990(2) - b** | 3w | Dose not stated | Once At Start | Captopril | 100 |  | HE | HE | HE |
| **Rezkalla 1990(2) - c** | 3w | Dose not stated | Once At Start | Captopril | 100 |  | HE | HE | HE |
| **Saegusa 2007** | 9w | 500 pfu | Once At Start | Candesartan | 10 |  | HE |  | HE |
| **Shirai 2004 - a** | 9w | Dose not stated | Once At Start | Candesartan | 0.05 | AM |  |  |  |
| **Shirai 2004 - b** | 9w | Dose not stated | Once At Start | Candesartan | 0.5 | AM |  |  |  |
| **Shirai 2004 - c** | 9w | Dose not stated | Once At Start | Candesartan | 5 | AM |  |  |  |
| **Sukumaran 2010(1)** | 8w | Dose not stated | Once At Start | Olmesartan | 10 | AM |  |  |  |
| **Sukumaran 2010(2)** | 8w | Dose not stated | Once At Start | Telmisartan | 10 | AM |  |  |  |
| **Sukumaran 2011(1)** | 8w | Dose not stated | Once At Start | Olmesartan | 10 | AM |  |  | HE |
| **Sukumaran 2011(2)** | 8w | Dose not stated | Once At Start | Telmisartan | 10 | AM |  |  |  |
| **Sukumaran 2011(3)** | 8w | Dose not stated | Once At Start | Olmesartan | 10 | AM |  |  |  |
| **Sukumaran 2012(1)** | 8w | Dose not stated | Once At Start | Telmisartan | 10 | AM |  |  |  |
| **Sukumaran 2012(2)** | 8w | Dose not stated | Once At Start | Telmisartan | 10 | AM |  |  |  |
| **Suzuki 1993 - a** | 4w | 100pfu | Once At Start | Captopril | 10 |  | HE | HE | HE |
| **Suzuki 1993 - b** | 4w | 100pfu | Once At Start | Captopril | 30 |  | HE | HE | HE |
| **Suzuki 1993 - c** | 4w | 100pfu | Once At Start | Captopril | 100 |  | HE | HE | HE |
| **Tachikawa 2004** | 8w | Dose not stated | Once At Start | Quinapril | 2 | AM |  |  |  |
| **Tachikawa 2003 - a** | 8w | Dose not stated | Once At Start | Valsartan | 10 | AM |  |  |  |
| **Tachikawa 2003 - b** | 8w | Dose not stated | Once At Start | Valsartan | 30 | AM |  |  |  |
| **Takada 1997 - a** | 2w | 100pfu | Once At Start | Captopril | 100 | MT | HE | HE | HE |
| **Takada 1997 - b** | 2w | 100pfu | Once At Start | Captopril | 100 | MT | HE | HE | HE |
| **Takamura 2016 - a** | 7w (23-26g) | Dose not stated | Day 1 and 8 | Aliskiren | 25 | AM |  |  | HE |
| **Takamura 2016 - b** | 7w (23-26g) | Dose not stated | Day 1 and 8 | Aliskiren | 50 | AM |  |  | HE |
| **Tanaka 1994 - a** | 4w | 100pfu | Once At Start | TCV-116 | 0.3 |  | HE | HE | HE |
| **Tanaka 1994 - b** | 4w | 100pfu | Once At Start | TCV-116 | 3 |  | HE | HE | HE |
| **Tominga 1991 - a** | 4w | 100pfu | Once At Start | Carteolol | 1 |  | HE | HE | HE |
| **Tominga 1991 - b** | 4w | 100pfu | Once At Start | Carteolol | 10 |  | HE | HE | HE |
| **Tominga 1991 - c** | 4w | 100pfu | Once At Start | Carteolol | 1 | HE |  | HE | HE |
| **Tominga 1991 - d** | 4w | 100pfu | Once At Start | Carteolol | 10 | HE |  | HE | HE |
| **Tominga 1991 - e** | 4w | 100pfu | Once At Start | Metoprolol | 30 | HE |  |  |  |
| **Tominga 1991 - f** | 4w | 100pfu | Once At Start | Carteolol | 1 | HE |  |  |  |
| **Tominga 1991 - g** | 4w | 100pfu | Once At Start | Carteolol | 10 | HE |  |  |  |
| **Veeraveedu 2006 - a** | 9w | Dose not stated | Once At Start | Amlodipine | 1 | AM |  |  |  |
| **Veeraveedu 2006 - b** | 9w | Dose not stated | Once At Start | Amlodipine | 5 | AM |  |  |  |
| **Veeraveedu 2006 - c** | 9w | Dose not stated | Once At Start | Pranidipine | 0.3 | AM |  |  |  |
| **Wahed 2004 - a** | 9w | Dose not stated | Once At Start | Pranidipine | 0.03 | AM |  |  |  |
| **Wahed 2004 - b** | 9w | Dose not stated | Once At Start | Pranidipine | 0.3 | AM |  |  |  |
| **Wahed 2005 - a** | 9w | Dose not stated | Once At Start | Eplerenone | 300 | AM |  |  |  |
| **Wahed 2005 - b** | 9w | Dose not stated | Once At Start | Eplerenone | 1000 | AM |  |  |  |
| **Wang 1997 - a** | 4w | 10pfu | Once At Start | Diltiazem | 60 |  | HE |  | HE |
| **Wang 1997 - b** | 4w | 10pfu | Once At Start | Amlodipine | 10 |  | HE |  | HE |
| **Wang 2005 - a** | 4w | 140pfu | Once At Start | Propranolol | 3 |  | HE |  | HE |
| **Wang 2005 - b** | 4w | 140pfu | Once At Start | Propranolol | 3 |  | HE |  | HE |
| **Wang 2005 - c** | 4w | 140pfu | Once At Start | Propranolol | 3 |  | HE |  | HE |
| **Wang 2005 - d** | 4w | 140pfu | Once At Start | Propranolol | 3 |  | HE |  | HE |
| **Wang 2005 - e** | 4w | 140pfu | Once At Start | Propranolol | 3 |  | HE |  | HE |
| **Wang 2005 - f** | 4w | 140pfu | Once At Start | Propranolol | 3 |  | HE |  | HE |
| **Wang 2005 - g** | 4w | 140pfu | Once At Start | Propranolol | 3 |  | HE |  | HE |
| **Wang 2005 - h** | 4w | 140pfu | Once At Start | Propranolol | 3 |  | HE |  | HE |
| **Wang 2005 - i** | 4w | 140pfu | Once At Start | Propranolol | 3 |  | HE |  | HE |
| **Watanabe 2000 - a** | 9w | Dose not stated | Once At Start | Carvedilol | 2 | AM |  |  |  |
| **Watanabe 2000 - b** | 9w | Dose not stated | Once At Start | Carvedilol | 20 | AM |  |  |  |
| **Watanabe 2001 - a** | 9w | Dose not stated | Once At Start | Bisoprolol | 0.1 | AM |  |  |  |
| **Watanabe 2001 - b** | 9w | Dose not stated | Once At Start | Bisoprolol | 1 | AM |  |  |  |
| **Watanabe 2001 - c** | 9w | Dose not stated | Once At Start | Bisoprolol | 10 | AM |  |  |  |
| **Watanabe 2003 (1) - a** | 9w | Dose not stated | Once At Start | Quinapril | 20 | AM |  |  |  |
| **Watanabe 2003 (1) - b** | 9w | Dose not stated | Once At Start | Candesartan | 0.5 | AM |  |  |  |
| **Watanabe 2003 (2) - a** | 9w | Dose not stated | Once At Start | Betaxolol | 0.1 | AM |  |  |  |
| **Watanabe 2003 (2) - b** | 9w | Dose not stated | Once At Start | Betaxolol | 1 | AM |  |  |  |
| **Watanabe 2003 (2) - c** | 9w | Dose not stated | Once At Start | Betaxolol | 10 | AM |  |  |  |
| **Watanabe 2003 (3) - a** | 9w | Dose not stated | Once At Start | Perindopril | 0.02 | AM |  |  |  |
| **Watanabe 2003 (3) - b** | 9w | Dose not stated | Once At Start | Perindopril | 0.2 | AM |  |  |  |
| **Watanabe 2003 (3) - c** | 9w | Dose not stated | Once At Start | Perindopril | 2 | AM |  |  |  |
| **Watanabe 2003 (3) - d** | 9w | Dose not stated | Once At Start | Enalapril | 2 | AM |  |  |  |
| **Watanabe 2003 (3) - e** | 9w | Dose not stated | Once At Start | Enalapril | 20 | AM |  |  |  |
| **Xiao 2009 - a** | 4w | 2pfu | Once At Start | Eplerenone | 2.5 | MT |  |  |  |
| **Xiao 2009 - a** | 4w | 2pfu | Once At Start | Eplerenone | 2.5 |  |  |  |  |
| **Xu 1992 - a** | 5w | Dose not stated | Once At Start | Verapamil | 0.2ml of 10^-8 mol/g |  | HE |  | HE |
| **Xu 1992 - b** | 5w | Dose not stated | Once At Start | Verapamil | 0.2ml of 10^-8 mol/g |  | HE |  | HE |
| **Xu 1992 - c** | 5w | Dose not stated | Once At Start | Verapamil | 0.2ml of 10^-8 mol/g |  | HE |  | HE |
| **Yue-Chun 2008 - a** | 8w | 1,000,000 pfu | Once At Start | Carvedilol | 10 |  | HE |  | HE |
| **Yue-Chun 2008 - b** | 8w | 1,000,000 pfu | Once At Start | Metoprolol | 30 |  | HE |  | HE |
| **Yue-Chun 2008 - c** | 8w | 1,000,000 pfu | Once At Start | Carvedilol | 10 |  | HE |  | HE |
| **Yue-Chun 2008 - d** | 8w | 1,000,000 pfu | Once At Start | Metoprolol | 30 |  | HE |  | HE |
| **Yue-Chun 2012 - a** | 4w | 1,000,000 pfu | Once At Start | Carvedilol | 10 |  | HE |  | HE |
| **Yue-Chun 2012 - b** | 4w | 1,000,000 pfu | Once At Start | Carvedilol | 10 |  | HE |  | HE |
| **Yue-Chun 2012 - c** | 4w | 1,000,000 pfu | Once At Start | Carvedilol | 10 |  | HE |  | HE |

Table S3. Characteristics of included comparisons for qualitative synthesis. HE, hematoxylin and eosin; MT, Masson’s trichrome; AZ, Azan-Mallory.

Quantitative data extraction tables

Key to main characteristics

|  | **Sex** | **Strain** | **Species** | **Myocarditis Induction** | **Drug Class** | **Measurement Method** | **Outcome** |
| --- | --- | --- | --- | --- | --- | --- | --- |
| **1** | Female | C3H | Mice | EMCV | ACE inhibitor | Manual | Inflammation |
| **2** | Male | A/J | Rat | AI - cardiac myosin | ARB | Mixed | Fibrosis |
| **3** | Mixed | BALB/c |  | CVB3 | Beta blocker | Automatic | Necrosis |
| **4** |  | A.CA/SnJ |  | Clozapine | Direct renin inhibitor | Not stated | Calcification |
| **5** |  | DBA/2 |  |  | CCB |  | Survival |
| **6** |  | CD1 |  |  | MRA |  | HW/BW |
| **7** |  | Lewis |  |  |  |  |  |
| **8** |  | Wistar |  |  |  |  |  |

Table S4. Key to main characteristics of included comparisons

Fibrosis

| First author | Sex | Strain | Species | Myocarditis induction | Drug class | Start of treatment | Length of treatment | Histological measurement method | SYRCLE risk of bias score | CAMARADES checklist score | Treated group number | Treated group fibrosis (%) | Treated group SD (%) | Control group number | Control group fibrosis (%) | Control group SD (%) |
| --- | --- | --- | --- | --- | --- | --- | --- | --- | --- | --- | --- | --- | --- | --- | --- | --- |
| Abdel-Wahab 2014 - a | 2 | 8 | 2 | 4 | 1 | 0 | 21 | 1 | 5 | 7 | 10 | 19 | 13.44 | 5.00 | 49.25 | 16.77 |
| Abdel-Wahab 2014 - b | 2 | 8 | 2 | 4 | 1 | 0 | 21 | 1 | 5 | 7 | 10 | 0.62 | 1.42 | 5.00 | 49.25 | 16.77 |
| Bahk 2007 - a | 2 | 2 | 1 | 2 | 2 | 0 | 21 | 4 | 7 | 3 | 19 | 15.75 | 17.44 | 10.00 | 43.75 | 15.02 |
| Bahk 2007 - b | 2 | 2 | 1 | 2 | 1 | 0 | 21 | 4 | 7 | 3 | 18 | 7 | 11.67 | 10.00 | 43.75 | 15.02 |
| Gluck 2010 - a | 2 | 4 | 1 | 3 | 3 | 0 | 14 | 1 | 4 | 3 | 19 | 25.00 | 20.02 | 22.00 | 50.00 | 19.81 |
| Gluck 2010 - b | 2 | 4 | 1 | 3 | 3 | 3 | 11 | 1 | 4 | 3 | 8 | 39.58 | 22.34 | 7.00 | 62.50 | 28.70 |
| Godsel 2003 | 2 | 2 | 1 | 2 | 1 | 0 | 21 | 1 | 8 | 5 | 6 | 20 | 20.00 | 19.00 | 32.50 | 15.00 |
| Guo 2009 | 2 | 3 | 1 | 3 | 1 | 42 | 28 | 3 | 7 | 4 | 18 | 9.43 | 1.18 | 18.00 | 14.88 | 1.11 |
| Juan 2003 - a | 2 | 7 | 2 | 2 | 1 | 28 | 30 | 1 | 4 | 4 | 9 | 22 | 12.00 | 3.67 | 32.00 | 7.66 |
| Juan 2003 - b | 2 | 7 | 2 | 2 | 1 | 28 | 30 | 1 | 4 | 4 | 11 | 13 | 9.95 | 3.67 | 32.00 | 7.66 |
| Juan 2003 - c | 2 | 7 | 2 | 2 | 1 | 28 | 30 | 1 | 4 | 4 | 11 | 6 | 3.32 | 3.67 | 32.00 | 7.66 |
| Kanda 1993 - a | 1 | 1 | 1 | 1 | 3 | 28 | 84 | 3 | 8 | 4 | 10 | 15.07 | 6.64 | 2.50 | 18.61 | 5.70 |
| Kanda 1993 - b | 1 | 1 | 1 | 1 | 3 | 28 | 84 | 3 | 8 | 4 | 10 | 14.92 | 7.74 | 2.50 | 18.61 | 5.70 |
| Kanda 1993 - c | 1 | 1 | 1 | 1 | 1 | 28 | 84 | 3 | 8 | 4 | 8 | 12.48 | 5.85 | 2.50 | 18.61 | 5.70 |
| Kanda 1993 - d | 1 | 1 | 1 | 1 | 1 | 28 | 84 | 3 | 8 | 4 | 4 | 8.37 | 3.17 | 2.50 | 18.61 | 5.70 |
| Kanda 1995 - a | 1 | 5 | 1 | 1 | 2 | 28 | 84 | 1 | 7 | 5 | 8 | 67.5 | 20.00 | 2.50 | 67.50 | 20.00 |
| Kanda 1995 - b | 1 | 5 | 1 | 1 | 2 | 28 | 84 | 1 | 7 | 5 | 8 | 55 | 20.00 | 2.50 | 67.50 | 20.00 |
| Kanda 1995 - c | 1 | 5 | 1 | 1 | 2 | 28 | 84 | 1 | 7 | 5 | 7 | 65 | 27.50 | 2.50 | 67.50 | 20.00 |
| Kanda 1995 - d | 1 | 5 | 1 | 1 | 1 | 28 | 84 | 1 | 7 | 5 | 7 | 32.5 | 12.50 | 2.50 | 67.50 | 20.00 |
| Kashimura 2003 - a | 2 | 7 | 2 | 2 | 1 | 28 | 44 | 3 | 1 | 3 | 7 | 10.7 | 4.26 | 2.00 | 12.40 | 1.82 |
| Kashimura 2003 - b | 2 | 7 | 2 | 2 | 1 | 28 | 44 | 3 | 1 | 3 | 5 | 8.87 | 3.16 | 2.00 | 12.40 | 1.82 |
| Kashimura 2003 - c | 2 | 7 | 2 | 2 | 2 | 28 | 44 | 3 | 1 | 3 | 6 | 7.71 | 4.88 | 2.00 | 12.40 | 1.82 |
| Kashimura 2003 - d | 2 | 7 | 2 | 2 | 2 | 28 | 44 | 3 | 1 | 3 | 6 | 10.09 | 5.55 | 2.00 | 12.40 | 1.82 |
| Li 2013 - b | 2 | 3 | 1 | 3 | 3 | 1 | 14 | 1 | 7 | 7 | 8 | 133.25 | 115.97 | 8.00 | 14.40 | 11.09 |
| Li-Sha 2013 - b | 2 | 3 | 1 | 3 | 3 | 1 | 14 | 2 | 5 | 7 | 8 | 157.75 | 172.53 | 8.00 | 14.41 | 10.35 |
| Ma 2001 | 2 | 7 | 2 | 2 | 1 | 28 | 30 | 3 | 4 | 3 | 8 | 6 | 212.13 | 8.00 | 29.00 | 16.97 |
| Reyes 1998 - a | 2 | 6 | 1 | 3 | 1 | 7 | 174 | 1 | 7 | 5 | 50 | 3 | 8.84 | 50.00 | 8.75 | 15.91 |
| Reyes 1998 - b | 2 | 6 | 1 | 3 | 1 | 7 | 294 | 1 | 7 | 5 | 25 | 7 | 11.25 | 25.00 | 9.00 | 13.75 |
| Shirai 2004 - a | 2 | 7 | 2 | 2 | 2 | 28 | 30 | 3 | 7 | 3 | 11 | 625 | 223.87 | 3.67 | 32.00 | 7.08 |
| Shirai 2004 - b | 2 | 7 | 2 | 2 | 2 | 28 | 30 | 3 | 7 | 3 | 13 | 500 | 270.42 | 3.67 | 32.00 | 7.08 |
| Shirai 2004 - c | 2 | 7 | 2 | 2 | 2 | 28 | 30 | 3 | 7 | 3 | 14 | 300 | 130.96 | 3.67 | 32.00 | 7.08 |
| Sukumaran 2010(1) | 2 | 7 | 2 | 2 | 2 | 28 | 28 | 3 | 4 | 4 | 10 | 17.5 | 10.12 | 10.00 | 33.80 | 4.11 |
| Sukumaran 2010(2) | 2 | 7 | 2 | 2 | 2 | 28 | 28 | 3 | 6 | 5 | 9 | 15 | 6.00 | 6.00 | 33.80 | 3.18 |
| Sukumaran 2011(1) | 2 | 7 | 2 | 2 | 2 | 0 | 21 | 2 | 4 | 4 | 8 | 32.5 | 5.94 | 8.00 | 56.75 | 6.79 |
| Sukumaran 2011(2) | 2 | 7 | 2 | 2 | 2 | 0 | 21 | 3 | 5 | 5 | 8 | 27.5 | 4.53 | 8.00 | 56.75 | 6.79 |
| Sukumaran 2011(3) | 2 | 7 | 2 | 2 | 2 | 0 | 21 | 4 | 4 | 5 | 6 | 35.5 | 5.14 | 6.00 | 54.00 | 5.88 |
| Sukumaran 2012(1) | 2 | 7 | 2 | 2 | 2 | 0 | 21 | 3 | 4 | 5 | 6 | 27.2 | 6.86 | 6.00 | 60.50 | 7.35 |
| Sukumaran 2012(2) | 2 | 7 | 2 | 2 | 2 | 28 | 28 | 3 | 4 | 5 | 6 | 18.75 | 6.12 | 8.00 | 35.50 | 3.68 |
| Tachikawa 2003 - a | 2 | 7 | 2 | 2 | 2 | 28 | 42 | 3 | 4 | 4 | 8 | 31.9 | 18.67 | 4.00 | 34.20 | 13.40 |
| Tachikawa 2003 - b | 2 | 7 | 2 | 2 | 2 | 28 | 42 | 3 | 4 | 4 | 8 | 25.6 | 15.27 | 4.00 | 34.20 | 13.40 |
| Tachikawa 2004 | 2 | 7 | 2 | 2 | 1 | 28 | 32 | 3 | 7 | 6 | 8 | 19 | 19.80 | 8.00 | 29.00 | 14.14 |
| Takada 1997 - a | 2 | 1 | 1 | 3 | 1 | 10 | 20 | 1 | 8 | 5 | 20 | 20 | 12.50 | 15.00 | 30.00 | 20.00 |
| Takada 1997 - b | 2 | 1 | 1 | 3 | 1 | 30 | 30 | 1 | 8 | 5 | 44 | 30 | 12.50 | 41.00 | 35.00 | 15.00 |
| Takamura 2016 - a | 2 | 3 | 1 | 2 | 4 | 0 | 22 | 3 | 4 | 5 | 10 | 7.3 | 3.79 | 5.00 | 17.60 | 6.26 |
| Takamura 2016 - b | 2 | 3 | 1 | 2 | 4 | 0 | 22 | 3 | 4 | 5 | 6 | 6.8 | 3.18 | 5.00 | 17.60 | 6.26 |
| Tominga 1991 - c | 2 | 5 | 1 | 1 | 3 | 14 | 14 | 1 | 5 | 4 | 10 | 42.5 | 22.50 | 12.00 | 47.50 | 0.90 |
| Tominga 1991 - d | 2 | 5 | 1 | 1 | 3 | 14 | 14 | 1 | 5 | 4 | 14 | 55 | 15.00 | 12.00 | 47.50 | 0.90 |
| Tominga 1991 - e | 2 | 5 | 1 | 1 | 3 | 14 | 90 | 1 | 5 | 4 | 7 | 52.5 | 27.50 | 6.67 | 52.50 | 0.90 |
| Tominga 1991 - f | 2 | 5 | 1 | 1 | 3 | 14 | 90 | 1 | 5 | 4 | 10 | 35 | 12.50 | 6.67 | 52.50 | 0.90 |
| Tominga 1991 - g | 2 | 5 | 1 | 1 | 3 | 14 | 90 | 1 | 5 | 4 | 6 | 30 | 20.00 | 6.67 | 52.50 | 0.90 |
| Veeraveedu 2006 - a | 2 | 7 | 2 | 2 | 5 | 28 | 28 | 3 | 8 | 6 | 9 | 29 | 15.00 | 2.67 | 36.00 | 3.27 |
| Veeraveedu 2006 - b | 2 | 7 | 2 | 2 | 5 | 28 | 28 | 3 | 8 | 6 | 10 | 19 | 12.65 | 2.67 | 36.00 | 3.27 |
| Veeraveedu 2006 - c | 2 | 7 | 2 | 2 | 5 | 28 | 28 | 3 | 8 | 6 | 10 | 13 | 6.32 | 2.67 | 36.00 | 3.27 |
| Wahed 2004 - a | 2 | 7 | 2 | 2 | 5 | 28 | 28 | 3 | 4 | 5 | 8 | 24 | 5.66 | 5.00 | 36.00 | 4.47 |
| Wahed 2004 - b | 2 | 7 | 2 | 2 | 5 | 28 | 28 | 3 | 4 | 5 | 10 | 16 | 6.32 | 5.00 | 36.00 | 4.47 |
| Wahed 2005 - a | 2 | 7 | 2 | 2 | 6 | 28 | 28 | 3 | 6 | 5 | 12 | 27 | 8.66 | 5.00 | 35.00 | 6.71 |
| Wahed 2005 - b | 2 | 7 | 2 | 2 | 6 | 28 | 28 | 3 | 6 | 5 | 15 | 13 | 11.62 | 5.00 | 35.00 | 6.71 |
| Watanabe 2000 - a | 2 | 7 | 2 | 2 | 3 | 28 | 60 | 3 | 4 | 5 | 10 | 12 | 3.16 | 5.00 | 31.00 | 4.47 |
| Watanabe 2000 - b | 2 | 7 | 2 | 2 | 3 | 28 | 60 | 3 | 4 | 5 | 10 | 24 | 12.65 | 5.00 | 31.00 | 4.47 |
| Watanabe 2001 - a | 2 | 7 | 2 | 2 | 3 | 28 | 30 | 3 | 1 | 5 | 9 | 44 | 15.00 | 2.67 | 42.00 | 9.80 |
| Watanabe 2001 - b | 2 | 7 | 2 | 2 | 3 | 28 | 30 | 3 | 1 | 5 | 13 | 37 | 21.63 | 2.67 | 42.00 | 9.80 |
| Watanabe 2001 - c | 2 | 7 | 2 | 2 | 3 | 28 | 30 | 3 | 1 | 5 | 13 | 35 | 25.24 | 2.67 | 42.00 | 9.80 |
| Watanabe 2003(1) - a | 2 | 7 | 2 | 2 | 1 | 28 | 30 | 3 | 4 | 4 | 14 | 9 | 11.22 | 5.50 | 36.00 | 14.07 |
| Watanabe 2003(1) - b | 2 | 7 | 2 | 2 | 2 | 28 | 30 | 3 | 4 | 4 | 12 | 26 | 13.86 | 5.50 | 36.00 | 14.07 |
| Watanabe 2003(2) - a | 2 | 7 | 2 | 2 | 3 | 28 | 30 | 3 | 4 | 5 | 10 | 31 | 28.46 | 2.67 | 38.00 | 14.70 |
| Watanabe 2003(2) - b | 2 | 7 | 2 | 2 | 3 | 28 | 30 | 3 | 4 | 5 | 13 | 27 | 28.84 | 2.67 | 38.00 | 14.70 |
| Watanabe 2003(2) - c | 2 | 7 | 2 | 2 | 3 | 28 | 30 | 3 | 4 | 5 | 13 | 25 | 32.45 | 2.67 | 38.00 | 14.70 |
| Watanabe 2003(3) - a | 2 | 7 | 2 | 2 | 1 | 28 | 30 | 3 | 4 | 3 | 13 | 26.5 | 9.01 | 2.20 | 36.00 | 3.86 |
| Watanabe 2003(3) - b | 2 | 7 | 2 | 2 | 1 | 28 | 30 | 3 | 4 | 3 | 15 | 15 | 8.13 | 2.20 | 36.00 | 3.86 |
| Watanabe 2003(3) - c | 2 | 7 | 2 | 2 | 1 | 28 | 30 | 3 | 4 | 3 | 15 | 7.5 | 5.42 | 2.20 | 36.00 | 3.86 |
| Watanabe 2003(3) - d | 2 | 7 | 2 | 2 | 1 | 28 | 30 | 3 | 4 | 3 | 13 | 29.5 | 12.26 | 2.20 | 36.00 | 3.86 |
| Watanabe 2003(3) - e | 2 | 7 | 2 | 2 | 1 | 28 | 30 | 3 | 4 | 3 | 15 | 15.6 | 7.75 | 2.20 | 36.00 | 3.86 |
| Xiao 2009 - a | 2 | 5 | 1 | 1 | 6 | 0 | 28 | 3 | 4 | 6 | 14 | 19.8 | 9.73 | 3.00 | 33.40 | 9.35 |

Table S5. Main characteristics of included comparisons: Fibrosis

1. First author; (b) Sex (c) Strain; (d) Species; (e) Myocarditis induction; (f) Drug class; (g) Start of treatment; (h) Length of treatment; (i) Histological measurement method; (j) SYRCLE risk of bias score; (k) CAMARADES checklist score; (l) Treated group number; (m) Treated group fibrosis (%); (n) Treated group SD (%); (o) Control group number; (p) Control group fibrosis (%); (q) Control group SD (%).

Necrosis

| First author | Sex | Strain | Species | Myocarditis induction | Drug class | Start of treatment | Length of treatment | Histological measurement method | SYRCLE risk of bias score | CAMARADES checklist score | Treated group number | Treated group necrosis (%) | Treated group SD (%) | Control group number | Control group necrosis (%) | Control group SD (%) |
| --- | --- | --- | --- | --- | --- | --- | --- | --- | --- | --- | --- | --- | --- | --- | --- | --- |
| Araki 1995 - a | 1 | 1 | 1 | 1 | 1 | 6 | 14 | 1 | 6 | 4 | 6 | 32.5 | 12.50 | 2.00 | 62.50 | 15.00 |
| Araki 1995 - b | 1 | 1 | 1 | 1 | 1 | 6 | 14 | 1 | 6 | 4 | 6 | 32.5 | 12.50 | 2.00 | 62.50 | 15.00 |
| Araki 1995 - c | 1 | 1 | 1 | 1 | 2 | 6 | 14 | 1 | 6 | 4 | 7 | 62.5 | 15.00 | 2.00 | 62.50 | 15.00 |
| Araki 1995 - d | 1 | 1 | 1 | 1 | 2 | 6 | 14 | 1 | 6 | 4 | 7 | 50 | 15.00 | 2.00 | 62.50 | 15.00 |
| Araki 1995 - e | 1 | 1 | 1 | 1 | 2 | 6 | 14 | 1 | 6 | 4 | 5 | 50 | 17.50 | 2.00 | 62.50 | 15.00 |
| Baba 2000 - a | 1 | 1 | 1 | 1 | 2 | 6 | 14 | 4 | 7 | 3 | 13 | 45.25 | 17.75 | 4.60 | 80.25 | 15.00 |
| Baba 2000 - b | 1 | 1 | 1 | 1 | 2 | 6 | 14 | 4 | 7 | 3 | 14 | 41 | 17.25 | 4.60 | 80.25 | 15.00 |
| Baba 2000 - c | 1 | 1 | 1 | 1 | 2 | 6 | 14 | 4 | 7 | 3 | 12 | 36.25 | 9.25 | 4.60 | 80.25 | 15.00 |
| Baba 2000 - d | 1 | 1 | 1 | 1 | 1 | 6 | 14 | 4 | 7 | 3 | 6 | 65.75 | 17.00 | 4.60 | 80.25 | 15.00 |
| Baba 2000 - e | 1 | 1 | 1 | 1 | 1 | 6 | 14 | 4 | 7 | 3 | 16 | 30.25 | 7.25 | 4.60 | 80.25 | 15.00 |
| Chen 2006 | 2 | 3 | 1 | 3 | 1 | 0 | 7 | 4 | 3 | 4 | 18 | 47 | 31.25 | 14.00 | 77.50 | 21.50 |
| Godsel 2003 | 2 | 2 | 1 | 2 | 1 | 0 | 21 | 1 | 8 | 5 | 6 | 12.5 | 12.50 | 19.00 | 27.50 | 17.50 |
| Kanda 1993 - a | 1 | 1 | 1 | 1 | 3 | 28 | 84 | 3 | 8 | 4 | 10 | 28.07 | 208.25 | 2.50 | 33.00 | 9.72 |
| Kanda 1993 - b | 1 | 1 | 1 | 1 | 3 | 28 | 84 | 3 | 8 | 4 | 10 | 25.48 | 275.25 | 2.50 | 33.00 | 9.72 |
| Kanda 1993 - c | 1 | 1 | 1 | 1 | 1 | 28 | 84 | 3 | 8 | 4 | 8 | 23.2 | 233.00 | 2.50 | 33.00 | 9.72 |
| Kanda 1993 - d | 1 | 1 | 1 | 1 | 1 | 28 | 84 | 3 | 8 | 4 | 4 | 14.53 | 250.25 | 2.50 | 33.00 | 9.72 |
| Kanda 1995 - a | 1 | 5 | 1 | 1 | 2 | 28 | 84 | 1 | 7 | 5 | 8 | 40 | 12.50 | 2.50 | 45.00 | 15.00 |
| Kanda 1995 - b | 1 | 5 | 1 | 1 | 2 | 28 | 84 | 1 | 7 | 5 | 8 | 25 | 7.50 | 2.50 | 45.00 | 15.00 |
| Kanda 1995 - c | 1 | 5 | 1 | 1 | 2 | 28 | 84 | 1 | 7 | 5 | 7 | 25 | 10.00 | 2.50 | 45.00 | 15.00 |
| Kanda 1995 - d | 1 | 5 | 1 | 1 | 1 | 28 | 84 | 1 | 7 | 5 | 7 | 25 | 15.00 | 2.50 | 45.00 | 15.00 |
| Li 2010 - a | 2 | 3 | 1 | 3 | 3 | 1 | 14 | 4 | 7 | 7 | 24 | 27.5 | 5.50 | 7.00 | 57.25 | 3.50 |
| Li 2010 - b | 2 | 3 | 1 | 3 | 3 | 1 | 14 | 4 | 7 | 7 | 13 | 54.5 | 3.25 | 7.00 | 57.25 | 3.50 |
| Li 2013 - a | 2 | 3 | 1 | 3 | 3 | 1 | 7 | 1 | 7 | 7 | 8 | 26.75 | 48.08 | 8.00 | 45.75 | 45.25 |
| Li 2013 - b | 2 | 3 | 1 | 3 | 3 | 1 | 14 | 1 | 7 | 7 | 8 | 42 | 62.93 | 8.00 | 67.50 | 36.77 |
| Li-Sha 2013 - a | 2 | 3 | 1 | 3 | 3 | 1 | 7 | 2 | 5 | 7 | 8 | 36.25 | 46.67 | 8.00 | 43.75 | 41.72 |
| Li-Sha 2013 - b | 2 | 3 | 1 | 3 | 3 | 1 | 14 | 2 | 5 | 7 | 8 | 28.25 | 24.75 | 8.00 | 59.50 | 36.77 |
| Liu 2009 | 2 | 5 | 1 | 1 | 5 | 0 | 5 | 3 | 2 | 5 | 10 | 1.2 | 1.30 | 10.00 | 3.80 | 1.80 |
| Nishio 2003 - a | 2 | 5 | 1 | 1 | 3 | 0 | 7 | 1 | 4 | 4 | 5 | 40 | 11.18 | 2.50 | 60.00 | 11.86 |
| Nishio 2003 - b | 2 | 5 | 1 | 1 | 3 | 0 | 7 | 1 | 4 | 4 | 5 | 27.5 | 11.18 | 2.50 | 60.00 | 11.86 |
| Nishio 2003 - c | 2 | 5 | 1 | 1 | 3 | 0 | 7 | 1 | 4 | 4 | 5 | 27.5 | 5.59 | 2.00 | 55.00 | 3.23 |
| Nishio 2003 - d | 2 | 5 | 1 | 1 | 3 | 0 | 7 | 1 | 4 | 4 | 5 | 60 | 33.54 | 2.00 | 55.00 | 3.23 |
| Nishio 2003 - e | 2 | 5 | 1 | 1 | 3 | 0 | 7 | 1 | 4 | 4 | 5 | 32.5 | 5.59 | 2.00 | 55.00 | 3.23 |
| Rezkalla 1988 - a | 3 | 6 | 1 | 3 | 3 | 0 | 3 | 1 | 5 | 4 | 10 | 2.5 | 7.91 | 10.00 | 0.01 | 0.01 |
| Rezkalla 1988 - b | 3 | 6 | 1 | 3 | 3 | 0 | 6 | 1 | 5 | 4 | 10 | 62.5 | 31.62 | 10.00 | 55.00 | 31.62 |
| Rezkalla 1988 - c | 3 | 6 | 1 | 3 | 3 | 0 | 10 | 1 | 5 | 4 | 10 | 57.5 | 31.62 | 10.00 | 47.50 | 31.62 |
| Rezkalla 1988 - d | 3 | 6 | 1 | 3 | 3 | 0 | 10 | 1 | 5 | 4 | 20 | 52.5 | 44.72 | 20.00 | 10.00 | 33.54 |
| Rezkalla 1990(1) | 3 | 6 | 1 | 3 | 1 | 3 | 6 | 1 | 5 | 5 | 16 | 2.25 | 1.41 | 16.00 | 4.13 | 1.55 |
| Rezkalla 1990(2) - a | 3 | 6 | 1 | 3 | 1 | 1 | 5 | 1 | 4 | 4 | 15 | 37.5 | 17.50 | 15.00 | 67.50 | 17.50 |
| Rezkalla 1990(2) - b | 3 | 6 | 1 | 3 | 1 | 10 | 10 | 1 | 4 | 4 | 14 | 25 | 17.50 | 14.00 | 22.50 | 12.50 |
| Rezkalla 1990(2) - c | 3 | 6 | 1 | 3 | 1 | 10 | 20 | 1 | 4 | 4 | 15 | 25 | 27.50 | 15.00 | 30.00 | 27.50 |
| Saegusa 2007 | 3 | 1 | 1 | 1 | 2 | 0 | 7 | 1 | 7 | 4 | 7 | 37.5 | 17.50 | 7.00 | 60.00 | 25.00 |
| Suzuki 1993 - a | 2 | 3 | 1 | 1 | 1 | 4 | 11 | 1 | 10 | 6 | 12 | 44 | 23.25 | 4.00 | 63.00 | 16.00 |
| Suzuki 1993 - b | 2 | 3 | 1 | 1 | 1 | 4 | 11 | 1 | 10 | 6 | 13 | 33.25 | 24.50 | 4.00 | 63.00 | 16.00 |
| Suzuki 1993 - c | 2 | 3 | 1 | 1 | 1 | 4 | 11 | 1 | 10 | 6 | 12 | 27.25 | 21.50 | 4.00 | 63.00 | 16.00 |
| Takada 1997 - a | 2 | 1 | 1 | 3 | 1 | 10 | 20 | 1 | 8 | 5 | 20 | 35 | 17.50 | 15.00 | 52.50 | 25.00 |
| Takada 1997 - b | 2 | 1 | 1 | 3 | 1 | 30 | 30 | 1 | 8 | 5 | 44 | 32.5 | 15.00 | 41.00 | 37.50 | 20.00 |
| Tanaka 1994 - a | 2 | 5 | 1 | 1 | 2 | 2 | 14 | 1 | 5 | 5 | 10 | 47.5 | 17.50 | 5.00 | 57.50 | 30.00 |
| Tanaka 1994 - b | 2 | 5 | 1 | 1 | 2 | 2 | 14 | 1 | 5 | 5 | 12 | 27.5 | 7.50 | 5.00 | 57.50 | 30.00 |
| Tominga 1991 - a | 2 | 5 | 1 | 1 | 3 | 0 | 14 | 1 | 5 | 4 | 7 | 37.5 | 20.00 | 3.00 | 37.50 | 17.50 |
| Tominga 1991 - b | 2 | 5 | 1 | 1 | 3 | 0 | 14 | 1 | 5 | 4 | 7 | 30 | 25.00 | 3.00 | 37.50 | 17.50 |
| Wang 1997 - a | 2 | 5 | 1 | 1 | 5 | 0 | 7 | 1 | 9 | 8 | 10 | 45 | 40.00 | 5.00 | 55.00 | 35.00 |
| Wang 1997 - b | 2 | 5 | 1 | 1 | 5 | 0 | 7 | 1 | 9 | 8 | 10 | 28.25 | 20.00 | 5.00 | 55.00 | 35.00 |
| Wang 2005 - a | 2 | 3 | 1 | 1 | 3 | -7 | 14 | 1 | 2 | 6 | 5 | 22.5 | 5.59 | 2.00 | 47.50 | 6.46 |
| Wang 2005 - b | 2 | 3 | 1 | 1 | 3 | 0 | 7 | 1 | 2 | 6 | 5 | 20 | 5.59 | 2.00 | 47.50 | 6.46 |
| Wang 2005 - c | 2 | 3 | 1 | 1 | 3 | 4 | 3 | 1 | 2 | 6 | 5 | 22.5 | 16.77 | 2.00 | 47.50 | 6.46 |
| Wang 2005 - d | 2 | 3 | 1 | 1 | 3 | -7 | 21 | 1 | 2 | 6 | 5 | 25 | 16.77 | 2.00 | 57.50 | 9.69 |
| Wang 2005 - e | 2 | 3 | 1 | 1 | 3 | 0 | 14 | 1 | 2 | 6 | 5 | 22.5 | 11.18 | 2.00 | 57.50 | 9.69 |
| Wang 2005 - f | 2 | 3 | 1 | 1 | 3 | 4 | 10 | 1 | 2 | 6 | 5 | 32.5 | 11.18 | 2.00 | 57.50 | 9.69 |
| Wang 2005 - g | 2 | 3 | 1 | 1 | 3 | -7 | 37 | 1 | 2 | 6 | 5 | 20 | 5.59 | 2.00 | 52.50 | 9.69 |
| Wang 2005 - h | 2 | 3 | 1 | 1 | 3 | 0 | 30 | 1 | 2 | 6 | 5 | 27.5 | 11.18 | 2.00 | 52.50 | 9.69 |
| Wang 2005 - i | 2 | 3 | 1 | 1 | 3 | 4 | 26 | 1 | 2 | 6 | 5 | 27.5 | 16.77 | 2.00 | 52.50 | 9.69 |
| Xu 1992 - a | 2 | 3 | 1 | 3 | 5 | 0 | 3 | 1 | 5 | 3 | 10 | 10 | 17.50 | 9.00 | 5.00 | 15.00 |
| Xu 1992 - b | 2 | 3 | 1 | 3 | 5 | 0 | 6 | 1 | 5 | 3 | 13 | 30 | 15.00 | 14.00 | 20.00 | 25.00 |
| Xu 1992 - c | 2 | 3 | 1 | 3 | 5 | 8 | 10 | 1 | 5 | 3 | 10 | 12.5 | 17.50 | 9.00 | 5.00 | 10.00 |
| Yue-Chun 2008 - a | 2 | 3 | 1 | 3 | 3 | 1 | 7 | 1 | 8 | 4 | 6 | 23 | 4.00 | 3.00 | 47.75 | 4.75 |
| Yue-Chun 2008 - b | 2 | 3 | 1 | 3 | 3 | 1 | 7 | 1 | 8 | 4 | 6 | 45.25 | 3.50 | 3.00 | 47.75 | 4.75 |
| Yue-Chun 2008 - c | 2 | 3 | 1 | 3 | 3 | 1 | 14 | 1 | 8 | 4 | 6 | 30.75 | 3.50 | 3.00 | 60.75 | 3.00 |
| Yue-Chun 2008 - d | 2 | 3 | 1 | 3 | 3 | 1 | 14 | 1 | 8 | 4 | 6 | 56.75 | 5.25 | 3.00 | 60.75 | 3.00 |
| Yue-Chun 2012 - a | 2 | 3 | 1 | 3 | 3 | 1 | 4 | 1 | 8 | 5 | 8 | 18.75 | 11.31 | 8.00 | 25.00 | 13.44 |
| Yue-Chun 2012 - b | 2 | 3 | 1 | 3 | 3 | 1 | 7 | 1 | 8 | 5 | 8 | 27.5 | 9.19 | 8.00 | 47.00 | 9.19 |
| Yue-Chun 2012 - c | 2 | 3 | 1 | 3 | 3 | 1 | 14 | 1 | 8 | 5 | 8 | 31.25 | 11.31 | 8.00 | 59.50 | 12.73 |

Table S6. Main characteristics of included comparisons: Necrosis

Calcification

| First author | Sex | Strain | Species | Myocarditis induction | Drug class | Start of treatment | Length of treatment | Histological measurement method | SYRCLE risk of bias score | CAMARADES checklist score | Treated group number | Treated group calcification (%) | Treated group SD (%) | Control group number | Control group calcification (%) | Control group SD (%) |
| --- | --- | --- | --- | --- | --- | --- | --- | --- | --- | --- | --- | --- | --- | --- | --- | --- |
| Araki 1995 - a | 1 | 1 | 1 | 1 | 1 | 6 | 14 | 1 | 6 | 4 | 6 | 25 | 0.01 | 2.00 | 37.50 | 12.50 |
| Araki 1995 - b | 1 | 1 | 1 | 1 | 1 | 6 | 14 | 1 | 6 | 4 | 6 | 25 | 0.01 | 2.00 | 37.50 | 12.50 |
| Araki 1995 - c | 1 | 1 | 1 | 1 | 2 | 6 | 14 | 1 | 6 | 4 | 7 | 37.5 | 12.50 | 2.00 | 37.50 | 12.50 |
| Araki 1995 - d | 1 | 1 | 1 | 1 | 2 | 6 | 14 | 1 | 6 | 4 | 7 | 30 | 10.00 | 2.00 | 37.50 | 12.50 |
| Araki 1995 - e | 1 | 1 | 1 | 1 | 2 | 6 | 14 | 1 | 6 | 4 | 5 | 30 | 10.00 | 2.00 | 37.50 | 12.50 |
| Kanda 1993 - a | 1 | 1 | 1 | 1 | 3 | 28 | 84 | 3 | 8 | 4 | 10 | 9.61 | 2.97 | 2.50 | 13.34 | 3.96 |
| Kanda 1993 - b | 1 | 1 | 1 | 1 | 3 | 28 | 84 | 3 | 8 | 4 | 10 | 9.46 | 3.77 | 2.50 | 13.34 | 3.96 |
| Kanda 1993 - c | 1 | 1 | 1 | 1 | 1 | 28 | 84 | 3 | 8 | 4 | 8 | 8.5 | 3.17 | 2.50 | 13.34 | 3.96 |
| Kanda 1993 - d | 1 | 1 | 1 | 1 | 1 | 28 | 84 | 3 | 8 | 4 | 4 | 5.87 | 3.37 | 2.50 | 13.34 | 3.96 |
| Rezkalla 1988 - a | 3 | 6 | 1 | 3 | 3 | 0 | 3 | 1 | 5 | 4 | 10 | 2.5 | 7.91 | 10.00 | 0.01 | 0.01 |
| Rezkalla 1988 - b | 3 | 6 | 1 | 3 | 3 | 0 | 6 | 1 | 5 | 4 | 10 | 47.5 | 15.81 | 10.00 | 47.50 | 23.72 |
| Rezkalla 1988 - c | 3 | 6 | 1 | 3 | 3 | 0 | 10 | 1 | 5 | 4 | 10 | 60 | 39.53 | 10.00 | 47.50 | 31.62 |
| Rezkalla 1988 - d | 3 | 6 | 1 | 3 | 3 | 0 | 10 | 1 | 5 | 4 | 20 | 55 | 55.90 | 20.00 | 10.00 | 33.54 |
| Rezkalla 1990(1) | 3 | 6 | 1 | 3 | 1 | 3 | 6 | 1 | 5 | 5 | 16.00 | 5.65 | 5.65 | 16.00 | 9.38 | 7.77 |
| Rezkalla 1990(2) - a | 3 | 6 | 1 | 3 | 1 | 1 | 5 | 1 | 4 | 4 | 15 | 2.5 | 7.50 | 15.00 | 27.50 | 22.50 |
| Rezkalla 1990(2) - b | 3 | 6 | 1 | 3 | 1 | 10 | 10 | 1 | 4 | 4 | 14 | 35 | 17.50 | 14.00 | 30.00 | 17.50 |
| Rezkalla 1990(2) - c | 3 | 6 | 1 | 3 | 1 | 10 | 20 | 1 | 4 | 4 | 15 | 35 | 27.50 | 15.00 | 35.00 | 25.00 |
| Suzuki 1993 - a | 2 | 3 | 1 | 1 | 1 | 4 | 11 | 1 | 10 | 6 | 12 | 35.5 | 21.50 | 4.00 | 54.50 | 24.25 |
| Suzuki 1993 - b | 2 | 3 | 1 | 1 | 1 | 4 | 11 | 1 | 10 | 6 | 13 | 25 | 21.75 | 4.00 | 54.50 | 24.25 |
| Suzuki 1993 - c | 2 | 3 | 1 | 1 | 1 | 4 | 11 | 1 | 10 | 6 | 12 | 14.75 | 23.25 | 4.00 | 54.50 | 24.25 |
| Takada 1997 - a | 2 | 1 | 1 | 3 | 1 | 10 | 20 | 1 | 8 | 5 | 20 | 20 | 12.50 | 15.00 | 17.50 | 12.50 |
| Takada 1997 - b | 2 | 1 | 1 | 3 | 1 | 30 | 30 | 1 | 8 | 5 | 44 | 35 | 15.00 | 41.00 | 40.00 | 25.00 |
| Tanaka 1994 - a | 2 | 5 | 1 | 1 | 2 | 2 | 14 | 1 | 5 | 5 | 10 | 47.5 | 17.50 | 5.00 | 52.50 | 27.50 |
| Tanaka 1994 - b | 2 | 5 | 1 | 1 | 2 | 2 | 14 | 1 | 5 | 5 | 12 | 27.5 | 7.50 | 5.00 | 52.50 | 27.50 |
| Tominga 1991 - a | 2 | 5 | 1 | 1 | 3 | 0 | 14 | 1 | 5 | 4 | 7 | 7.5 | 12.50 | 3.00 | 5.00 | 10.00 |
| Tominga 1991 - b | 2 | 5 | 1 | 1 | 3 | 0 | 14 | 1 | 5 | 4 | 7 | 7.5 | 10.00 | 3.00 | 5.00 | 10.00 |
| Tominga 1991 - c | 2 | 5 | 1 | 1 | 3 | 14 | 14 | 1 | 5 | 4 | 10 | 45 | 25.00 | 12.00 | 50.00 | 25.00 |
| Tominga 1991 - d | 2 | 5 | 1 | 1 | 3 | 14 | 14 | 1 | 5 | 4 | 14 | 50 | 15.00 | 12.00 | 50.00 | 25.00 |

Table S7. Main characteristics of included comparisons: Calcification

Inflammation

| First author | Sex | Strain | Species | Myocarditis induction | Drug class | Start of treatment | Length of treatment | Histological measurement method | SYRCLE risk of bias score | CAMARADES checklist score | Treated group number | Treated group inflammation (%) | Treated group SD (%) | Control group number | Control group inflammation (%) | Control group SD (%) |
| --- | --- | --- | --- | --- | --- | --- | --- | --- | --- | --- | --- | --- | --- | --- | --- | --- |
| Araki 1995 - a | 1 | 1 | 1 | 1 | 1 | 6 | 14 | 1 | 6 | 4 | 6 | 15 | 7.50 | 2 | 32.5 | 10.00 |
| Araki 1995 - b | 1 | 1 | 1 | 1 | 1 | 6 | 14 | 1 | 6 | 4 | 6 | 22.5 | 10.00 | 2 | 32.5 | 10.00 |
| Araki 1995 - c | 1 | 1 | 1 | 1 | 2 | 6 | 14 | 1 | 6 | 4 | 7 | 35 | 7.50 | 2 | 32.5 | 10.00 |
| Araki 1995 - d | 1 | 1 | 1 | 1 | 2 | 6 | 14 | 1 | 6 | 4 | 7 | 25 | 10.00 | 2 | 32.5 | 10.00 |
| Araki 1995 - e | 1 | 1 | 1 | 1 | 2 | 6 | 14 | 1 | 6 | 4 | 5 | 45 | 10.00 | 2 | 32.5 | 10.00 |
| Baba 2000 - a | 1 | 1 | 1 | 1 | 2 | 6 | 14 | 4 | 7 | 3 | 13 | 48.25 | 17.75 | 4.6 | 75.75 | 14.25 |
| Baba 2000 - b | 1 | 1 | 1 | 1 | 2 | 6 | 14 | 4 | 7 | 3 | 14 | 50.75 | 17.75 | 4.6 | 75.75 | 14.25 |
| Baba 2000 - c | 1 | 1 | 1 | 1 | 2 | 6 | 14 | 4 | 7 | 3 | 12 | 33.5 | 10.25 | 4.6 | 75.75 | 14.25 |
| Baba 2000 - d | 1 | 1 | 1 | 1 | 1 | 6 | 14 | 4 | 7 | 3 | 6 | 68.5 | 17.00 | 4.6 | 75.75 | 14.25 |
| Baba 2000 - e | 1 | 1 | 1 | 1 | 1 | 6 | 14 | 4 | 7 | 3 | 16 | 75.75 | 14.25 | 4.6 | 75.75 | 14.25 |
| Bahk 2007 - a | 2 | 2 | 1 | 2 | 2 | 0 | 21 | 4 | 7 | 3 | 19 | 34.21 | 22.88 | 10 | 67.5 | 18.97 |
| Bahk 2007 - b | 2 | 2 | 1 | 2 | 1 | 0 | 21 | 4 | 7 | 3 | 18 | 15.25 | 12.73 | 10 | 67.5 | 18.97 |
| Chen 2006 | 2 | 3 | 1 | 3 | 1 | 0 | 7 | 4 | 3 | 4 | 18 | 50 | 23.25 | 14 | 84 | 18.50 |
| Gluck 2010 - a | 2 | 4 | 1 | 3 | 3 | 0 | 14 | 1 | 4 | 3 | 19 | 35.42 | 15.02 | 22 | 56.25 | 24.77 |
| Gluck 2010 - b | 2 | 4 | 1 | 3 | 3 | 3 | 11 | 1 | 4 | 3 | 8 | 54.17 | 27.92 | 7 | 70.83 | 34.45 |
| Godsel 2003 | 2 | 2 | 1 | 2 | 1 | 0 | 21 | 1 | 8 | 5 | 6 | 30 | 10.00 | 19 | 37.5 | 20.00 |
| Li 2010 - a | 2 | 3 | 1 | 3 | 3 | 1 | 14 | 4 | 7 | 7 | 24 | 25.5 | 2.25 | 7 | 43.75 | 2.75 |
| Li 2010 - b | 2 | 3 | 1 | 3 | 3 | 1 | 14 | 4 | 7 | 7 | 13 | 40.5 | 3.00 | 7 | 43.75 | 2.75 |
| Li 2013 - a | 2 | 3 | 1 | 3 | 3 | 1 | 7 | 1 | 7 | 7 | 8 | 47.5 | 24.04 | 8 | 73.5 | 30.41 |
| Li 2013 - b | 2 | 3 | 1 | 3 | 3 | 1 | 14 | 1 | 7 | 7 | 8 | 27 | 28.99 | 8 | 49 | 21.92 |
| Nishio 2003 - a | 2 | 5 | 1 | 1 | 3 | 0 | 7 | 1 | 4 | 4 | 5 | 37.5 | 11.18 | 2.5 | 55 | 3.95 |
| Nishio 2003 - b | 2 | 5 | 1 | 1 | 3 | 0 | 7 | 1 | 4 | 4 | 5 | 30 | 5.59 | 2.5 | 55 | 3.95 |
| Nishio 2003 - c | 2 | 5 | 1 | 1 | 3 | 0 | 7 | 1 | 4 | 4 | 5 | 27.5 | 5.59 | 2 | 50 | 6.46 |
| Nishio 2003 - d | 2 | 5 | 1 | 1 | 3 | 0 | 7 | 1 | 4 | 4 | 5 | 57.5 | 27.95 | 2 | 50 | 6.46 |
| Nishio 2003 - e | 2 | 5 | 1 | 1 | 3 | 0 | 7 | 1 | 4 | 4 | 5 | 40 | 11.18 | 2 | 50 | 6.46 |
| Rezkalla 1988 - a | 3 | 6 | 1 | 3 | 3 | 0 | 3 | 1 | 5 | 4 | 10 | 5 | 7.91 | 10 | 0.01 | 0.01 |
| Rezkalla 1988 - b | 3 | 6 | 1 | 3 | 3 | 0 | 6 | 1 | 5 | 4 | 10 | 67.5 | 15.81 | 10 | 70 | 15.81 |
| Rezkalla 1988 - c | 3 | 6 | 1 | 3 | 3 | 0 | 10 | 1 | 5 | 4 | 10 | 72.5 | 31.62 | 10 | 67.5 | 15.81 |
| Rezkalla 1988 - d | 3 | 6 | 1 | 3 | 3 | 0 | 10 | 1 | 5 | 4 | 20 | 27.5 | 33.54 | 20 | 7.5 | 11.18 |
| Rezkalla 1990(1) | 3 | 6 | 1 | 3 | 1 | 3 | 6 | 1 | 5 | 5 | 16 | 45.00 | 17.81 | 16.00 | 54.50 | 13.57 |
| Rezkalla 1990(2) - a | 3 | 6 | 1 | 3 | 1 | 1 | 5 | 1 | 4 | 4 | 15 | 27.5 | 15.00 | 15 | 65 | 20.00 |
| Rezkalla 1990(2) - b | 3 | 6 | 1 | 3 | 1 | 10 | 10 | 1 | 4 | 4 | 14 | 40 | 22.50 | 14 | 32.5 | 15.00 |
| Rezkalla 1990(2) - c | 3 | 6 | 1 | 3 | 1 | 10 | 20 | 1 | 4 | 4 | 15 | 42.5 | 32.50 | 15 | 45 | 32.50 |
| Saegusa 2007 |  | 1 | 1 | 1 | 2 | 0 | 7 | 1 | 7 | 4 | 7 | 27.5 | 10.00 | 7 | 52.5 | 22.50 |
| Sukumaran 2011(1) | 2 | 7 | 2 | 2 | 2 | 0 | 21 | 2 | 4 | 4 | 8 | 25 | 84.85 | 8 | 67.5 | 42.43 |
| Suzuki 1993 - a | 2 | 3 | 1 | 1 | 1 | 4 | 11 | 1 | 10 | 6 | 12 | 44 | 23.25 | 4 | 65 | 15.50 |
| Suzuki 1993 - b | 2 | 3 | 1 | 1 | 1 | 4 | 11 | 1 | 10 | 6 | 13 | 33.25 | 24.50 | 4 | 65 | 15.50 |
| Suzuki 1993 - c | 2 | 3 | 1 | 1 | 1 | 4 | 11 | 1 | 10 | 6 | 12 | 29.5 | 20.25 | 4 | 65 | 15.50 |
| Takada 1997 - a | 2 | 1 | 1 | 3 | 1 | 10 | 20 | 1 | 8 | 5 | 20 | 27.5 | 10.00 | 15 | 45 | 12.50 |
| Takada 1997 - b | 2 | 1 | 1 | 3 | 1 | 30 | 30 | 1 | 8 | 5 | 44 | 20 | 12.50 | 41 | 22.5 | 10.00 |
| Takamura 2016 - a | 2 | 3 | 1 | 2 | 4 | 0 | 22 | 3 | 4 | 5 | 10 | 3.6 | 2.21 | 5 | 13.9 | 5.59 |
| Takamura 2016 - b | 2 | 3 | 1 | 2 | 4 | 0 | 22 | 3 | 4 | 5 | 6 | 3.8 | 2.69 | 5 | 13.9 | 5.59 |
| Tanaka 1994 - a | 2 | 5 | 1 | 1 | 2 | 2 | 14 | 1 | 5 | 5 | 10 | 52.5 | 12.50 | 5 | 65 | 32.50 |
| Tanaka 1994 - b | 2 | 5 | 1 | 1 | 2 | 2 | 14 | 1 | 5 | 5 | 12 | 35 | 17.50 | 5 | 65 | 32.50 |
| Tominga 1991 - a | 2 | 5 | 1 | 1 | 3 | 0 | 14 | 1 | 5 | 4 | 7 | 35 | 12.50 | 3 | 35 | 15.00 |
| Tominga 1991 - b | 2 | 5 | 1 | 1 | 3 | 0 | 14 | 1 | 5 | 4 | 7 | 30 | 15.00 | 3 | 35 | 15.00 |
| Tominga 1991 - c | 2 | 5 | 1 | 1 | 3 | 14 | 14 | 1 | 5 | 4 | 10 | 25 | 12.50 | 12 | 32.5 | 12.50 |
| Tominga 1991 - d | 2 | 5 | 1 | 1 | 3 | 14 | 14 | 1 | 5 | 4 | 14 | 35 | 7.50 | 12 | 32.5 | 12.50 |
| Wang 1997 - a | 2 | 5 | 1 | 1 | 5 | 0 | 7 | 1 | 9 | 8 | 10 | 50 | 22.50 | 5 | 57.5 | 17.50 |
| Wang 1997 - b | 2 | 5 | 1 | 1 | 5 | 0 | 7 | 1 | 9 | 8 | 10 | 30 | 22.50 | 5 | 57.5 | 17.50 |
| Wang 2005 - a | 2 | 3 | 1 | 1 | 3 | -7 | 14 | 1 | 2 | 6 | 5 | 25 | 5.59 | 2 | 55 | 6.46 |
| Wang 2005 - b | 2 | 3 | 1 | 1 | 3 | 0 | 7 | 1 | 2 | 6 | 5 | 22.5 | 5.59 | 2 | 55 | 6.46 |
| Wang 2005 - c | 2 | 3 | 1 | 1 | 3 | 4 | 3 | 1 | 2 | 6 | 5 | 30 | 11.18 | 2 | 55 | 6.46 |
| Wang 2005 - d | 2 | 3 | 1 | 1 | 3 | -7 | 21 | 1 | 2 | 6 | 5 | 32.5 | 11.18 | 2 | 72.5 | 12.92 |
| Wang 2005 - e | 2 | 3 | 1 | 1 | 3 | 0 | 14 | 1 | 2 | 6 | 5 | 32.5 | 11.18 | 2 | 72.5 | 12.92 |
| Wang 2005 - f | 2 | 3 | 1 | 1 | 3 | 4 | 10 | 1 | 2 | 6 | 5 | 40 | 11.18 | 2 | 72.5 | 12.92 |
| Wang 2005 - g | 2 | 3 | 1 | 1 | 3 | -7 | 37 | 1 | 2 | 6 | 5 | 35 | 11.18 | 2 | 67.5 | 9.69 |
| Wang 2005 - h | 2 | 3 | 1 | 1 | 3 | 0 | 30 | 1 | 2 | 6 | 5 | 37.5 | 11.18 | 2 | 67.5 | 9.69 |
| Wang 2005 - i | 2 | 3 | 1 | 1 | 3 | 4 | 26 | 1 | 2 | 6 | 5 | 42.5 | 11.18 | 2 | 67.5 | 9.69 |
| Xu 1992 - a | 2 | 3 | 1 | 3 | 5 | 0 | 3 | 1 | 5 | 3 | 10 | 15 | 17.50 | 9 | 7.5 | 17.50 |
| Xu 1992 - b | 2 | 3 | 1 | 3 | 5 | 0 | 6 | 1 | 5 | 3 | 13 | 37.5 | 22.50 | 14 | 20 | 17.50 |
| Xu 1992 - c | 2 | 3 | 1 | 3 | 5 | 8 | 10 | 1 | 5 | 3 | 10 | 20 | 10.00 | 9 | 15 | 10.00 |
| Yue-Chun 2008 - a | 2 | 3 | 1 | 3 | 3 | 1 | 7 | 1 | 8 | 4 | 6 | 26.25 | 4.00 | 3 | 69.5 | 5.25 |
| Yue-Chun 2008 - b | 2 | 3 | 1 | 3 | 3 | 1 | 7 | 1 | 8 | 4 | 6 | 66.75 | 5.00 | 3 | 69.5 | 5.25 |
| Yue-Chun 2008 - c | 2 | 3 | 1 | 3 | 3 | 1 | 14 | 1 | 8 | 4 | 6 | 23 | 2.75 | 3 | 49.5 | 3.50 |
| Yue-Chun 2008 - d | 2 | 3 | 1 | 3 | 3 | 1 | 14 | 1 | 8 | 4 | 6 | 45.25 | 3.75 | 3 | 49.5 | 3.50 |
| Yue-Chun 2012 - a | 2 | 3 | 1 | 3 | 3 | 1 | 4 | 1 | 8 | 5 | 8 | 28.25 | 9.19 | 8 | 34.5 | 12.73 |
| Yue-Chun 2012 - b | 2 | 3 | 1 | 3 | 3 | 1 | 7 | 1 | 8 | 5 | 8 | 40.75 | 18.38 | 8 | 65.75 | 18.38 |
| Yue-Chun 2012 - c | 2 | 3 | 1 | 3 | 3 | 1 | 14 | 1 | 8 | 5 | 8 | 25 | 0.01 | 8 | 47 | 9.19 |

Table S8. Main characteristics of included comparisons: Inflammation

Survival

| First author | Sex | Strain | Species | Myocarditis induction | Drug class | Start of treatment | Length of treatment | Histological measurement method | SYRCLE risk of bias score | CAMARADES checklist score | Treated group number | Treated group survival (%) | Control group number | Control group survival (%) |
| --- | --- | --- | --- | --- | --- | --- | --- | --- | --- | --- | --- | --- | --- | --- |
| Araki 1995 - e | 1 | 1 | 1 | 1 | 2 | 6 | 14 | 1 | 6 | 4 | 7 | 71 | 1.2 | 100 |
| Araki 1995 - a | 1 | 1 | 1 | 1 | 1 | 6 | 14 | 1 | 6 | 4 | 6 | 100 | 1.2 | 100 |
| Araki 1995 - b | 1 | 1 | 1 | 1 | 1 | 6 | 14 | 1 | 6 | 4 | 6 | 100 | 1.2 | 100 |
| Araki 1995 - c | 1 | 1 | 1 | 1 | 2 | 6 | 14 | 1 | 6 | 4 | 7 | 100 | 1.2 | 100 |
| Araki 1995 - d | 1 | 1 | 1 | 1 | 2 | 6 | 14 | 1 | 6 | 4 | 7 | 100 | 1.2 | 100 |
| Kashimura 2003 - b | 2 | 7 | 2 | 2 | 1 | 28 | 44 | 3 | 1 | 3 | 5 | 100 | 1.75 | 100 |
| Kashimura 2003 - c | 2 | 7 | 2 | 2 | 2 | 28 | 44 | 3 | 1 | 3 | 6 | 100 | 1.75 | 100 |
| Kashimura 2003 - d | 2 | 7 | 2 | 2 | 2 | 28 | 44 | 3 | 1 | 3 | 6 | 100 | 1.75 | 100 |
| Kashimura 2003 - a | 2 | 7 | 2 | 2 | 1 | 28 | 44 | 3 | 1 | 3 | 7 | 100 | 1.75 | 100 |
| Kanda 1993 - d | 1 | 1 | 1 | 1 | 1 | 28 | 84 | 3 | 8 | 4 | 12 | 33 | 2.5 | 100 |
| Kanda 1993 - c | 1 | 1 | 1 | 1 | 1 | 28 | 84 | 3 | 8 | 4 | 10 | 80 | 2.5 | 100 |
| Kanda 1995 - c | 1 | 5 | 1 | 1 | 2 | 28 | 84 | 1 | 7 | 5 | 8 | 87.5 | 2.5 | 100 |
| Kanda 1995 - d | 1 | 5 | 1 | 1 | 1 | 28 | 84 | 1 | 7 | 5 | 8 | 87.5 | 2.5 | 100 |
| Kanda 1995 - a | 1 | 5 | 1 | 1 | 2 | 28 | 84 | 1 | 7 | 5 | 8 | 100 | 2.5 | 100 |
| Kanda 1995 - b | 1 | 5 | 1 | 1 | 2 | 28 | 84 | 1 | 7 | 5 | 8 | 100 | 2.5 | 100 |
| Kanda 1993 - a | 1 | 1 | 1 | 1 | 3 | 28 | 84 | 3 | 8 | 4 | 10 | 100 | 2.5 | 100 |
| Kanda 1993 - b | 1 | 1 | 1 | 1 | 3 | 28 | 84 | 3 | 8 | 4 | 10 | 100 | 2.5 | 100 |
| Watanabe 2003(3) - a | 2 | 7 | 2 | 2 | 1 | 28 | 30 | 3 | 4 | 3 | 15 | 87 | 3 | 73 |
| Watanabe 2003(3) - d | 2 | 7 | 2 | 2 | 1 | 28 | 30 | 3 | 4 | 3 | 15 | 87 | 3 | 73 |
| Watanabe 2003(3) - b | 2 | 7 | 2 | 2 | 1 | 28 | 30 | 3 | 4 | 3 | 15 | 100 | 3 | 73 |
| Watanabe 2003(3) - c | 2 | 7 | 2 | 2 | 1 | 28 | 30 | 3 | 4 | 3 | 15 | 100 | 3 | 73 |
| Watanabe 2003(3) - e | 2 | 7 | 2 | 2 | 1 | 28 | 30 | 3 | 4 | 3 | 15 | 100 | 3 | 73 |
| Tachikawa 2003 - a | 2 | 7 | 2 | 2 | 2 | 28 | 42 | 3 | 4 | 4 | 8 | 1 | 4 | 100 |
| Tachikawa 2003 - b | 2 | 7 | 2 | 2 | 2 | 28 | 42 | 3 | 4 | 4 | 8 | 1 | 4 | 100 |
| Veeraveedu 2006 - a | 2 | 7 | 2 | 2 | 5 | 28 | 28 | 3 | 8 | 6 | 12 | 75 | 4 | 66.7 |
| Tominga 1991 - a | 2 | 5 | 1 | 1 | 3 | 0 | 14 | 1 | 5 | 4 | 9 | 77.8 | 4 | 75 |
| Tominga 1991 - b | 2 | 5 | 1 | 1 | 3 | 0 | 14 | 1 | 5 | 4 | 9 | 77.8 | 4 | 75 |
| Veeraveedu 2006 - b | 2 | 7 | 2 | 2 | 5 | 28 | 28 | 3 | 8 | 6 | 10 | 100 | 4 | 66.7 |
| Veeraveedu 2006 - c | 2 | 7 | 2 | 2 | 5 | 28 | 28 | 3 | 8 | 6 | 10 | 100 | 4 | 66.7 |
| Watanabe 2001 - a | 2 | 7 | 2 | 2 | 3 | 28 | 30 | 3 | 1 | 5 | 13 | 69 | 4.33 | 62 |
| Watanabe 2003(2) - a | 2 | 7 | 2 | 2 | 3 | 28 | 30 | 3 | 4 | 5 | 13 | 77 | 4.33 | 62 |
| Watanabe 2001 - b | 2 | 7 | 2 | 2 | 3 | 28 | 30 | 3 | 1 | 5 | 13 | 100 | 4.33 | 62 |
| Watanabe 2001 - c | 2 | 7 | 2 | 2 | 3 | 28 | 30 | 3 | 1 | 5 | 13 | 100 | 4.33 | 62 |
| Watanabe 2003(2) - b | 2 | 7 | 2 | 2 | 3 | 28 | 30 | 3 | 4 | 5 | 13 | 100 | 4.33 | 62 |
| Watanabe 2003(2) - c | 2 | 7 | 2 | 2 | 3 | 28 | 30 | 3 | 4 | 5 | 13 | 100 | 4.33 | 62 |
| Shirai 2004 - a | 2 | 7 | 2 | 2 | 2 | 28 | 30 | 3 | 7 | 3 | 15 | 73 | 5 | 73 |
| Juan 2003 - a | 3 | 7 | 2 | 2 | 1 | 28 | 30 | 1 | 4 | 4 | 11 | 82 | 5 | 73 |
| Shirai 2004 - b | 2 | 7 | 2 | 2 | 2 | 28 | 30 | 3 | 7 | 3 | 15 | 87 | 5 | 73 |
| Shirai 2004 - c | 2 | 7 | 2 | 2 | 2 | 28 | 30 | 3 | 7 | 3 | 15 | 93 | 5 | 73 |
| Juan 2003 - b | 3 | 7 | 2 | 2 | 1 | 28 | 30 | 1 | 4 | 4 | 11 | 100 | 5 | 73 |
| Juan 2003 - c | 3 | 7 | 2 | 2 | 1 | 28 | 30 | 1 | 4 | 4 | 11 | 100 | 5 | 73 |
| Wang 1997 - a | 2 | 5 | 1 | 1 | 5 | 0 | 7 | 1 | 9 | 8 | 10 | 80 | 6 | 83.3 |
| Wang 1997 - b | 2 | 5 | 1 | 1 | 5 | 0 | 7 | 1 | 9 | 8 | 10 | 90 | 6 | 83.3 |
| Watanabe 2000 - a | 2 | 7 | 2 | 2 | 3 | 28 | 60 | 3 | 4 | 5 | 10 | 100 | 6 | 83.3 |
| Watanabe 2000 - b | 2 | 7 | 2 | 2 | 3 | 28 | 60 | 3 | 4 | 5 | 10 | 100 | 6 | 83.3 |
| Nishio 2003 - d | 2 | 5 | 1 | 1 | 3 | 0 | 14 | 1 | 4 | 4 | 20 | 35 | 6.67 | 40 |
| Nishio 2003 - e | 2 | 5 | 1 | 1 | 3 | 0 | 14 | 1 | 4 | 4 | 20 | 50 | 6.67 | 40 |
| Nishio 2003 - c | 2 | 5 | 1 | 1 | 3 | 0 | 14 | 1 | 4 | 4 | 20 | 85 | 6.67 | 40 |
| Wahed 2004 - a | 2 | 7 | 2 | 2 | 5 | 28 | 28 | 3 | 4 | 5 | 11 | 73 | 7.5 | 66.7 |
| Wahed 2005 - a | 2 | 7 | 2 | 2 | 6 | 28 | 28 | 3 | 6 | 5 | 15 | 80 | 7.5 | 66.7 |
| Watanabe 2003(1) - b | 2 | 7 | 2 | 2 | 2 | 28 | 30 | 3 | 4 | 4 | 14 | 86 | 7.5 | 73 |
| Wahed 2004 - b | 2 | 7 | 2 | 2 | 5 | 28 | 28 | 3 | 4 | 5 | 10 | 100 | 7.5 | 66.7 |
| Watanabe 2003(1) - a | 2 | 7 | 2 | 2 | 1 | 28 | 30 | 3 | 4 | 4 | 14 | 100 | 7.5 | 73 |
| Wahed 2005 - b | 2 | 7 | 2 | 2 | 6 | 28 | 28 | 3 | 6 | 5 | 15 | 100 | 7.5 | 66.7 |
| Xu 1992 - a | 2 | 3 | 1 | 3 | 5 | 0 | 3 | 1 | 5 | 3 | 10 | 100 | 9 | 100 |
| Nishio 2003 - a | 2 | 5 | 1 | 1 | 3 | 0 | 14 | 1 | 4 | 4 | 20 | 70 | 10 | 45 |
| Nishio 2003 - b | 2 | 5 | 1 | 1 | 3 | 0 | 14 | 1 | 4 | 4 | 20 | 85 | 10 | 45 |
| Sukumaran 2010(2) | 2 | 7 | 2 | 2 | 2 | 28 | 28 | 3 | 6 | 5 | 10 | 90 | 10 | 60 |
| Xu 1992 - c | 2 | 3 | 1 | 3 | 5 | 8 | 10 | 1 | 5 | 3 | 10 | 100 | 10 | 90 |
| Tominga 1991 - g | 2 | 5 | 1 | 1 | 3 | 14 | 90 | 1 | 5 | 4 | 9 | 66.7 | 10.33 | 64.5 |
| Tominga 1991 - f | 2 | 5 | 1 | 1 | 3 | 14 | 90 | 1 | 5 | 4 | 13 | 76.9 | 10.33 | 64.5 |
| Tominga 1991 - e | 2 | 5 | 1 | 1 | 3 | 14 | 90 | 1 | 5 | 4 | 9 | 77.8 | 10.33 | 64.5 |
| Tanaka 1994 - a | 2 | 5 | 1 | 1 | 2 | 2 | 14 | 1 | 5 | 5 | 21 | 48 | 10.5 | 48 |
| Tanaka 1994 - b | 2 | 5 | 1 | 1 | 2 | 2 | 14 | 1 | 5 | 5 | 21 | 57 | 10.5 | 48 |
| Suzuki 1993 - a | 2 | 3 | 1 | 1 | 1 | 4 | 11 | 1 | 10 | 6 | 24 | 50 | 12.33 | 46 |
| Suzuki 1993 - b | 2 | 3 | 1 | 1 | 1 | 4 | 11 | 1 | 10 | 6 | 21 | 62 | 12.33 | 46 |
| Suzuki 1993 - c | 2 | 3 | 1 | 1 | 1 | 4 | 11 | 1 | 10 | 6 | 26 | 73 | 12.33 | 46 |
| Wang 2005 - f | 2 | 3 | 1 | 1 | 3 | 4 | 10 | 1 | 2 | 6 | 40 | 85 | 13.33 | 70 |
| Wang 2005 - i | 2 | 3 | 1 | 1 | 3 | 4 | 26 | 1 | 2 | 6 | 40 | 85 | 13.33 | 70 |
| Wang 2005 - b | 2 | 3 | 1 | 1 | 3 | 0 | 7 | 1 | 2 | 6 | 40 | 87.5 | 13.33 | 85 |
| Wang 2005 - e | 2 | 3 | 1 | 1 | 3 | 0 | 14 | 1 | 2 | 6 | 40 | 87.5 | 13.33 | 70 |
| Wang 2005 - h | 2 | 3 | 1 | 1 | 3 | 0 | 30 | 1 | 2 | 6 | 40 | 87.5 | 13.33 | 70 |
| Wang 2005 - d | 2 | 3 | 1 | 1 | 3 | -7 | 21 | 1 | 2 | 6 | 40 | 90 | 13.33 | 70 |
| Wang 2005 - g | 2 | 3 | 1 | 1 | 3 | -7 | 37 | 1 | 2 | 6 | 40 | 90 | 13.33 | 70 |
| Wang 2005 - c | 2 | 3 | 1 | 1 | 3 | 4 | 3 | 1 | 2 | 6 | 40 | 92.5 | 13.33 | 85 |
| Wang 2005 - a | 2 | 3 | 1 | 1 | 3 | -7 | 14 | 1 | 2 | 6 | 40 | 97.5 | 13.33 | 85 |
| Tominga 1991 - c | 2 | 5 | 1 | 1 | 3 | 14 | 14 | 1 | 5 | 4 | 12 | 83.3 | 13.5 | 88.9 |
| Tominga 1991 - d | 2 | 5 | 1 | 1 | 3 | 14 | 14 | 1 | 5 | 4 | 16 | 87.5 | 13.5 | 88.9 |
| Xu 1992 - b | 2 | 3 | 1 | 3 | 5 | 0 | 6 | 1 | 5 | 3 | 14 | 93 | 14 | 100 |
| Li 2010 - b | 2 | 3 | 1 | 3 | 3 | 1 | 14 | 4 | 7 | 7 | 30 | 43 | 15 | 47 |
| Li 2010 - a | 2 | 3 | 1 | 3 | 3 | 1 | 14 | 4 | 7 | 7 | 30 | 80 | 15 | 47 |
| Rezkalla 1990(2) - b | 3 | 6 | 1 | 3 | 1 | 10 | 10 | 1 | 4 | 4 | 15 | 93 | 15 | 93 |
| Ma 2001 | 2 | 7 | 2 | 2 | 1 | 28 | 30 | 3 | 4 | 3 | 11 | 100 | 15 | 73 |
| Rezkalla 1990(2) - a | 3 | 6 | 1 | 3 | 1 | 1 | 5 | 1 | 4 | 4 | 15 | 100 | 15 | 100 |
| Rezkalla 1990(2) - c | 3 | 6 | 1 | 3 | 1 | 10 | 20 | 1 | 4 | 4 | 15 | 100 | 15 | 100 |
| Xiao 2009 - b | 2 | 5 | 1 | 1 | 6 | 6 | 21 | 3 | 4 | 6 | 40 | 20 | 20 | 15 |
| Xiao 2009 - a | 2 | 5 | 1 | 1 | 6 | 0 | 28 | 3 | 4 | 6 | 40 | 35 | 20 | 15 |
| Rezkalla 1988 - d | 3 | 6 | 1 | 3 | 3 | 0 | 10 | 1 | 5 | 4 | 20 | 40 | 20 | 100 |
| Takada 1997 - a | 2 | 1 | 1 | 3 | 1 | 10 | 20 | 1 | 8 | 5 | 20 | 100 | 20 | 75 |
| Saegusa 2007 | 3 | 1 | 1 | 1 | 2 | 0 | 7 | 1 | 7 | 4 | 30 | 60 | 30 | 18 |
| Li-Sha 2013 - b | 2 | 3 | 1 | 3 | 3 | 1 | 14 | 2 | 5 | 7 | 30 | 73 | 30 | 53.1 |
| Li-Sha 2013 - a | 2 | 3 | 1 | 3 | 3 | 1 | 7 | 2 | 5 | 7 | 30 | 80 | 30 | 63.3 |
| Chen 2006 | 3 | 3 | 1 | 3 | 1 | 0 | 7 | 4 | 3 | 4 | 33 | 87.9 | 36 | 66.7 |
| Yue-Chun 2012 - c | 2 | 3 | 1 | 3 | 3 | 1 | 14 | 1 | 8 | 5 | 40 | 75 | 40 | 50 |
| Yue-Chun 2012 - b | 2 | 3 | 1 | 3 | 3 | 1 | 7 | 1 | 8 | 5 | 40 | 95.9 | 40 | 83.4 |
| Yue-Chun 2012 - a | 2 | 3 | 1 | 3 | 3 | 1 | 4 | 1 | 8 | 5 | 40 | 100 | 40 | 95.9 |
| Takada 1997 - b | 2 | 1 | 1 | 3 | 1 | 30 | 30 | 1 | 8 | 5 | 46 | 96 | 46 | 89 |
| Reyes 1998 - b | 2 | 6 | 1 | 3 | 1 | 7 | 294 | 1 | 7 | 5 | 55 | 96.1 | 64 | 96.9 |
| Reyes 1998 - a | 2 | 6 | 1 | 3 | 1 | 7 | 174 | 1 | 7 | 5 | 115 | 92.3 | 115 | 99 |

Table S9. Main characteristics of included comparisons: Survival

HW/BW

| First author | Sex | Strain | Species | Myocarditis induction | Drug class | Start of treatment | Length of treatment | Histological measurement method | SYRCLE risk of bias score | CAMARADES checklist score | Treated group number | Treated group HW/BW (%) | Treated group SD (%) | Control group number | Control group HW/BW (%) | Control group SD (%) |
| --- | --- | --- | --- | --- | --- | --- | --- | --- | --- | --- | --- | --- | --- | --- | --- | --- |
| Nishio 2003 - c | 2 | 5 | 1 | 1 | 3 | 0 | 7 | 1 | 4 | 4 | 5 | 6.2 | 1.57 | 2.00 | 7.80 | 0.78 |
| Nishio 2003 - d | 2 | 5 | 1 | 1 | 3 | 0 | 7 | 1 | 4 | 4 | 5 | 8.3 | 1.34 | 2.00 | 7.80 | 0.78 |
| Nishio 2003 - e | 2 | 5 | 1 | 1 | 3 | 0 | 7 | 1 | 4 | 4 | 5 | 7.9 | 0.67 | 2.00 | 7.80 | 0.78 |
| Kashimura 2003 - a | 2 | 7 | 2 | 2 | 1 | 28 | 44 | 3 | 1 | 3 | 7 | 3.4 | 0.50 | 2.00 | 4.10 | 0.50 |
| Araki 1995 - a | 1 | 1 | 1 | 1 | 1 | 6 | 14 | 1 | 6 | 4 | 6 | 7 | 0.60 | 2.00 | 8.20 | 0.90 |
| Araki 1995 - b | 1 | 1 | 1 | 1 | 1 | 6 | 14 | 1 | 6 | 4 | 6 | 9.2 | 0.80 | 2.00 | 8.20 | 0.90 |
| Araki 1995 - c | 1 | 1 | 1 | 1 | 2 | 6 | 14 | 1 | 6 | 4 | 7 | 9.6 | 0.80 | 2.00 | 8.20 | 0.90 |
| Araki 1995 - d | 1 | 1 | 1 | 1 | 2 | 6 | 14 | 1 | 6 | 4 | 7 | 8.4 | 0.70 | 2.00 | 8.20 | 0.90 |
| Araki 1995 - e | 1 | 1 | 1 | 1 | 2 | 6 | 14 | 1 | 6 | 4 | 5 | 6.3 | 0.70 | 2.00 | 8.20 | 0.90 |
| Kashimura 2003 - b | 2 | 7 | 2 | 2 | 1 | 28 | 44 | 3 | 1 | 3 | 5 | 2.9 | 0.40 | 2.00 | 4.10 | 0.50 |
| Kashimura 2003 - c | 2 | 7 | 2 | 2 | 2 | 28 | 44 | 3 | 1 | 3 | 6 | 3.3 | 0.50 | 2.00 | 4.10 | 0.50 |
| Kashimura 2003 - d | 2 | 7 | 2 | 2 | 2 | 28 | 44 | 3 | 1 | 3 | 6 | 3.3 | 0.30 | 2.00 | 4.10 | 0.50 |
| Kanda 1993 - a | 1 | 1 | 1 | 1 | 3 | 28 | 84 | 3 | 8 | 4 | 10 | 6.2 | 0.90 | 2.50 | 7.20 | 1.00 |
| Kanda 1993 - b | 1 | 1 | 1 | 1 | 3 | 28 | 84 | 3 | 8 | 4 | 10 | 5.3 | 1.30 | 2.50 | 7.20 | 1.00 |
| Kanda 1993 - c | 1 | 1 | 1 | 1 | 1 | 28 | 84 | 3 | 8 | 4 | 8 | 5.9 | 1.60 | 2.50 | 7.20 | 1.00 |
| Kanda 1993 - d | 1 | 1 | 1 | 1 | 1 | 28 | 84 | 3 | 8 | 4 | 4 | 4.9 | 0.50 | 2.50 | 7.20 | 1.00 |
| Kanda 1995 - a | 1 | 5 | 1 | 1 | 2 | 28 | 84 | 1 | 7 | 5 | 8 | 5.5 | 0.40 | 2.50 | 5.30 | 0.50 |
| Kanda 1995 - b | 1 | 5 | 1 | 1 | 2 | 28 | 84 | 1 | 7 | 5 | 8 | 4.9 | 0.40 | 2.50 | 5.30 | 0.50 |
| Kanda 1995 - c | 1 | 5 | 1 | 1 | 2 | 28 | 84 | 1 | 7 | 5 | 7 | 5 | 0.50 | 2.50 | 5.30 | 0.50 |
| Kanda 1995 - d | 1 | 5 | 1 | 1 | 1 | 28 | 84 | 1 | 7 | 5 | 7 | 5.2 | 0.60 | 2.50 | 5.30 | 0.50 |
| Nishio 2003 - a | 2 | 5 | 1 | 1 | 3 | 0 | 7 | 1 | 4 | 4 | 5 | 6.3 | 0.67 | 2.50 | 7.80 | 0.95 |
| Nishio 2003 - b | 2 | 5 | 1 | 1 | 3 | 0 | 7 | 1 | 4 | 4 | 5 | 6 | 0.45 | 2.50 | 7.80 | 0.95 |
| Veeraveedu 2006 - a | 2 | 7 | 2 | 2 | 5 | 28 | 28 | 3 | 8 | 6 | 9 | 4.1 | 0.96 | 2.67 | 4.20 | 0.16 |
| Veeraveedu 2006 - b | 2 | 7 | 2 | 2 | 5 | 28 | 28 | 3 | 8 | 6 | 10 | 3.6 | 0.38 | 2.67 | 4.20 | 0.16 |
| Veeraveedu 2006 - c | 2 | 7 | 2 | 2 | 5 | 28 | 28 | 3 | 8 | 6 | 10 | 3.2 | 2.53 | 2.67 | 4.20 | 0.16 |
| Watanabe 2001 - a | 2 | 7 | 2 | 2 | 3 | 28 | 30 | 3 | 1 | 5 | 9 | 5.34 | 0.87 | 2.67 | 5.48 | 0.57 |
| Watanabe 2001 - b | 2 | 7 | 2 | 2 | 3 | 28 | 30 | 3 | 1 | 5 | 13 | 5.33 | 1.33 | 2.67 | 5.48 | 0.57 |
| Watanabe 2001 - c | 2 | 7 | 2 | 2 | 3 | 28 | 30 | 3 | 1 | 5 | 13 | 5.38 | 1.26 | 2.67 | 5.48 | 0.57 |
| Watanabe 2003(2) - a | 2 | 7 | 2 | 2 | 3 | 28 | 30 | 3 | 4 | 5 | 10 | 3.59 | 0.63 | 2.67 | 3.82 | 0.34 |
| Watanabe 2003(2) - b | 2 | 7 | 2 | 2 | 3 | 28 | 30 | 3 | 4 | 5 | 13 | 3.26 | 1.12 | 2.67 | 3.82 | 0.34 |
| Watanabe 2003(2) - c | 2 | 7 | 2 | 2 | 3 | 28 | 30 | 3 | 4 | 5 | 13 | 3.31 | 0.72 | 2.67 | 3.82 | 0.34 |
| Tominga 1991 - a | 2 | 5 | 1 | 1 | 3 | 0 | 14 | 1 | 5 | 4 | 7 | 6.8 | 1.00 | 3.00 | 7.00 | 1.20 |
| Tominga 1991 - b | 2 | 5 | 1 | 1 | 3 | 0 | 14 | 1 | 5 | 4 | 7 | 6.5 | 0.90 | 3.00 | 7.00 | 1.20 |
| Yue-Chun 2008 - a | 2 | 3 | 1 | 3 | 3 | 1 | 7 | 1 | 8 | 4 | 6 | 5.25 | 0.35 | 3.00 | 6.10 | 0.31 |
| Yue-Chun 2008 - b | 2 | 3 | 1 | 3 | 3 | 1 | 7 | 1 | 8 | 4 | 6 | 5.95 | 0.32 | 3.00 | 6.10 | 0.31 |
| Yue-Chun 2008 - c | 2 | 3 | 1 | 3 | 3 | 1 | 14 | 1 | 8 | 4 | 6 | 5.51 | 0.26 | 3.00 | 6.93 | 0.44 |
| Yue-Chun 2008 - d | 2 | 3 | 1 | 3 | 3 | 1 | 14 | 1 | 8 | 4 | 6 | 6.73 | 0.61 | 3.00 | 6.93 | 0.44 |
| Juan 2003 - a | 2 | 7 | 2 | 2 | 1 | 28 | 30 | 1 | 4 | 4 | 9 | 4.04 | 0.72 | 3.67 | 4.38 | 0.42 |
| Juan 2003 - b | 2 | 7 | 2 | 2 | 1 | 28 | 30 | 1 | 4 | 4 | 11 | 3.81 | 0.73 | 3.67 | 4.38 | 0.42 |
| Juan 2003 - c | 2 | 7 | 2 | 2 | 1 | 28 | 30 | 1 | 4 | 4 | 11 | 2.95 | 0.27 | 3.67 | 4.38 | 0.42 |
| Shirai 2004 - a | 2 | 7 | 2 | 2 | 2 | 28 | 30 | 3 | 7 | 3 | 11 | 3.7 | 0.66 | 3.67 | 3.80 | 0.38 |
| Shirai 2004 - b | 2 | 7 | 2 | 2 | 2 | 28 | 30 | 3 | 7 | 3 | 13 | 3.3 | 0.36 | 3.67 | 3.80 | 0.38 |
| Shirai 2004 - c | 2 | 7 | 2 | 2 | 2 | 28 | 30 | 3 | 7 | 3 | 14 | 3.1 | 0.37 | 3.67 | 3.80 | 0.38 |
| Suzuki 1993 - a | 2 | 3 | 1 | 1 | 1 | 4 | 11 | 1 | 10 | 6 | 12 | 5.2 | 0.80 | 4.00 | 5.65 | 0.84 |
| Suzuki 1993 - b | 2 | 3 | 1 | 1 | 1 | 4 | 11 | 1 | 10 | 6 | 13 | 4.88 | 0.63 | 4.00 | 5.65 | 0.84 |
| Suzuki 1993 - c | 2 | 3 | 1 | 1 | 1 | 4 | 11 | 1 | 10 | 6 | 12 | 4.78 | 0.52 | 4.00 | 5.65 | 0.84 |
| Baba 2000 - a | 1 | 1 | 1 | 1 | 2 | 6 | 14 | 4 | 7 | 3 | 13 | 5.1 | 1.40 | 4.60 | 6.60 | 1.80 |
| Baba 2000 - b | 1 | 1 | 1 | 1 | 2 | 6 | 14 | 4 | 7 | 3 | 14 | 5.2 | 1.50 | 4.60 | 6.60 | 1.80 |
| Baba 2000 - c | 1 | 1 | 1 | 1 | 2 | 6 | 14 | 4 | 7 | 3 | 12 | 5.1 | 1.00 | 4.60 | 6.60 | 1.80 |
| Baba 2000 - d | 1 | 1 | 1 | 1 | 1 | 6 | 14 | 4 | 7 | 3 | 6 | 4.2 | 0.80 | 4.60 | 6.60 | 1.80 |
| Baba 2000 - e | 1 | 1 | 1 | 1 | 1 | 6 | 14 | 4 | 7 | 3 | 16 | 5.2 | 1.00 | 4.60 | 6.60 | 1.80 |
| Takamura 2016 - a | 2 | 3 | 1 | 2 | 4 | 0 | 22 | 3 | 4 | 5 | 10 | 5.4 | 0.63 | 5.00 | 6.50 | 0.67 |
| Takamura 2016 - b | 2 | 3 | 1 | 2 | 4 | 0 | 22 | 3 | 4 | 5 | 6 | 4.9 | 0.73 | 5.00 | 6.50 | 0.67 |
| Wahed 2004 - a | 2 | 7 | 2 | 2 | 5 | 28 | 28 | 3 | 4 | 5 | 8 | 4.07 | 0.45 | 5.00 | 4.32 | 0.29 |
| Wahed 2004 - b | 2 | 7 | 2 | 2 | 5 | 28 | 28 | 3 | 4 | 5 | 10 | 3.36 | 0.38 | 5.00 | 4.32 | 0.29 |
| Wahed 2005 - a | 2 | 7 | 2 | 2 | 6 | 28 | 28 | 3 | 6 | 5 | 12 | 3.9 | 0.55 | 5.00 | 4.45 | 0.22 |
| Wahed 2005 - b | 2 | 7 | 2 | 2 | 6 | 28 | 28 | 3 | 6 | 5 | 15 | 3.3 | 0.77 | 5.00 | 4.45 | 0.22 |
| Wang 1997 - a | 2 | 5 | 1 | 1 | 5 | 0 | 7 | 1 | 9 | 8 | 10 | 5.8 | 0.60 | 5.00 | 6.90 | 0.70 |
| Wang 1997 - b | 2 | 5 | 1 | 1 | 5 | 0 | 7 | 1 | 9 | 8 | 10 | 4.7 | 0.20 | 5.00 | 6.90 | 0.70 |
| Watanabe 2000 - a | 2 | 7 | 2 | 2 | 3 | 28 | 60 | 3 | 4 | 5 | 10 | 2.87 | 0.16 | 5.00 | 3.35 | 0.16 |
| Watanabe 2000 - b | 2 | 7 | 2 | 2 | 3 | 28 | 60 | 3 | 4 | 5 | 10 | 3.03 | 0.25 | 5.00 | 3.35 | 0.16 |
| Sukumaran 2010(2) | 2 | 7 | 2 | 2 | 2 | 28 | 28 | 3 | 6 | 5 | 9 | 3.1 | 0.30 | 6.00 | 4.50 | 1.32 |
| Sukumaran 2011(3) | 2 | 7 | 2 | 2 | 2 | 0 | 21 | 4 | 4 | 5 | 6 | 4.9 | 0.59 | 6.00 | 6.20 | 1.22 |
| Sukumaran 2012(1) | 2 | 7 | 2 | 2 | 2 | 0 | 21 | 3 | 4 | 5 | 6 | 4.6 | 0.49 | 6.00 | 5.90 | 0.61 |
| Xiao 2009 - a | 2 | 5 | 1 | 1 | 6 | 0 | 28 | 3 | 4 | 6 | 14 | 7 | 2.24 | 6.00 | 9.70 | 3.43 |
| Tominga 1991 - e | 2 | 5 | 1 | 1 | 3 | 14 | 90 | 1 | 5 | 4 | 7 | 6.8 | 0.50 | 6.67 | 7.10 | 1.00 |
| Tominga 1991 - f | 2 | 5 | 1 | 1 | 3 | 14 | 90 | 1 | 5 | 4 | 10 | 5.3 | 0.40 | 6.67 | 7.10 | 1.00 |
| Tominga 1991 - g | 2 | 5 | 1 | 1 | 3 | 14 | 90 | 1 | 5 | 4 | 6 | 5.1 | 0.70 | 6.67 | 7.10 | 1.00 |
| Li 2010 - a | 2 | 3 | 1 | 3 | 3 | 1 | 14 | 4 | 7 | 7 | 24 | 5.4 | 0.39 | 7.00 | 6.81 | 0.50 |
| Li 2010 - b | 2 | 3 | 1 | 3 | 3 | 1 | 14 | 4 | 7 | 7 | 13 | 6.69 | 0.68 | 7.00 | 6.81 | 0.50 |
| Saegusa 2007 |  | 1 | 1 | 1 | 2 | 0 | 7 | 1 | 7 | 4 | 7 | 5.6 | 0.80 | 7.00 | 7.20 | 1.10 |
| Li 2013 - a | 2 | 3 | 1 | 3 | 3 | 1 | 7 | 1 | 7 | 7 | 8 | 5.2 | 1.90 | 8.00 | 6.00 | 1.98 |
| Li 2013 - b | 2 | 3 | 1 | 3 | 3 | 1 | 14 | 1 | 7 | 7 | 8 | 5.73 | 1.33 | 8.00 | 6.84 | 2.29 |
| Li-Sha 2013 - a | 2 | 3 | 1 | 3 | 3 | 1 | 7 | 2 | 5 | 7 | 8 | 5.18 | 1.92 | 8.00 | 5.85 | 1.24 |
| Li-Sha 2013 - b | 2 | 3 | 1 | 3 | 3 | 1 | 14 | 2 | 5 | 7 | 8 | 5.68 | 1.47 | 8.00 | 6.87 | 2.01 |
| Ma 2001 | 2 | 7 | 2 | 2 | 1 | 28 | 30 | 3 | 4 | 3 | 8 | 2.95 | 0.23 | 8.00 | 4.38 | 0.62 |
| Sukumaran 2011(1) | 2 | 7 | 2 | 2 | 2 | 0 | 21 | 2 | 4 | 4 | 8 | 4.3 | 0.57 | 8.00 | 5.70 | 1.24 |
| Sukumaran 2011(2) | 2 | 7 | 2 | 2 | 2 | 0 | 21 | 3 | 5 | 5 | 8 | 3.9 | 0.54 | 8.00 | 5.70 | 1.24 |
| Sukumaran 2012(2) | 2 | 7 | 2 | 2 | 2 | 28 | 28 | 3 | 4 | 5 | 6 | 5.1 | 0.59 | 8.00 | 4.25 | 1.13 |
| Yue-Chun 2012 - a | 2 | 3 | 1 | 3 | 3 | 1 | 4 | 1 | 8 | 5 | 8 | 4.72 | 0.28 | 8.00 | 4.82 | 0.34 |
| Yue-Chun 2012 - b | 2 | 3 | 1 | 3 | 3 | 1 | 7 | 1 | 8 | 5 | 8 | 5.07 | 0.25 | 8.00 | 5.84 | 0.28 |
| Yue-Chun 2012 - c | 2 | 3 | 1 | 3 | 3 | 1 | 14 | 1 | 8 | 5 | 8 | 5.43 | 0.20 | 8.00 | 6.59 | 0.59 |
| Bahk 2007 - a | 2 | 2 | 1 | 2 | 2 | 0 | 21 | 4 | 7 | 3 | 19 | 4.2 | 0.87 | 10.00 | 5.50 | 1.26 |
| Bahk 2007 - b | 2 | 2 | 1 | 2 | 1 | 0 | 21 | 4 | 7 | 3 | 18 | 4 | 0.42 | 10.00 | 5.50 | 1.26 |
| Sukumaran 2010(1) | 2 | 7 | 2 | 2 | 2 | 28 | 28 | 3 | 4 | 4 | 10 | 3.8 | 0.63 | 10.00 | 4.50 | 1.58 |
| Tominga 1991 - c | 2 | 5 | 1 | 1 | 3 | 14 | 14 | 1 | 5 | 4 | 10 | 8.5 | 2.90 | 12.00 | 9.00 | 2.70 |
| Tominga 1991 - d | 2 | 5 | 1 | 1 | 3 | 14 | 14 | 1 | 5 | 4 | 14 | 9.3 | 2.40 | 12.00 | 9.00 | 2.70 |
| Rezkalla 1990(2) - b | 3 | 6 | 1 | 3 | 1 | 10 | 10 | 1 | 4 | 4 | 14 | 4.4 | 0.60 | 14.00 | 6.10 | 1.40 |
| Rezkalla 1990(2) - a | 3 | 6 | 1 | 3 | 1 | 1 | 5 | 1 | 4 | 4 | 15 | 4.6 | 0.50 | 15.00 | 6.10 | 0.90 |
| Rezkalla 1990(2) - c | 3 | 6 | 1 | 3 | 1 | 10 | 20 | 1 | 4 | 4 | 15 | 4.9 | 1.30 | 15.00 | 6.30 | 2.00 |
| Takada 1997 - a | 2 | 1 | 1 | 3 | 1 | 10 | 20 | 1 | 8 | 5 | 20 | 6.9 | 1.50 | 15.00 | 9.40 | 2.30 |
| Godsel 2003 | 2 | 2 | 1 | 2 | 1 | 0 | 21 | 1 | 8 | 5 | 6 | 3.97 | 2.52 | 19.00 | 4.83 | 7.19 |
| Takada 1997 - b | 2 | 1 | 1 | 3 | 1 | 30 | 30 | 1 | 8 | 5 | 23 | 6.5 | 1.50 | 24.00 | 6.40 | 1.40 |
| Reyes 1998 - b | 2 | 6 | 1 | 3 | 1 | 7 | 294 | 1 | 7 | 5 | 25 | 4.7 | 0.70 | 25.00 | 5.40 | 0.20 |
| Reyes 1998 - a | 2 | 6 | 1 | 3 | 1 | 7 | 174 | 1 | 7 | 5 | 50 | 4.8 | 1.10 | 50.00 | 5.20 | 0.92 |

Table S10. Main characteristics of included comparisons: HW/BW

Results

Necrosis

| **Experimental factor** | **Analyses (n)** | **WMD** | **(95% CI)** | **% Weight** | **P-value** | **I^2^ % (P)** |
| --- | --- | --- | --- | --- | --- | --- |
| Drug Class |  |  |  |  |  | 91 (<0.01) |
| ACE Inhibitor | 18 | 19.40 | 12.10, 26.71 | 24.9 | 0.000 | 85 (<0.01) |
| ARB | 12 | 23.59 | 14.53, 32.65 | 14.5 | 0.000 | 45 (0.04) |
| Beta-blocker | 35 | 16.98 | 11.47, 22.49 | 51.8 | 0.000 | 90 (<0.01) |
| CCB | 6 | -0.93 | -9.83, 7.98 | 8.8 | 0.800 | 38 (0.15) |
| Induction Method |  |  |  |  | 0.000 | 91 (<0.01) |
| EMCV | 43 | 23.33 | 19.53, 27.13 | 52.8 | 0.000 | 85 (<0.01) |
| Cardiac myosin | 1 | 15.00 | 2.27, 27.73 | 1.8 | 0.021 | N/A |
| CVB3 | 27 | 8.95 | 2.41, 15.49 | 45.3 | 0.009 | 95 (<0.01) |
| Species |  |  |  |  | 0.000 | 91 (<0.01) |
| Mouse | 71 | 16.91 | 13.25, 20.71 | 100.0 | 0.000 | 91 (<0.01) |
| Sex |  |  |  |  | 0.000 | 91 (<0.01) |
| Female | 18 | 26.04 | 18.50, 33.58 | 17.7 | 0.000 | 44 (0.02) |
| Male | 44 | 17.88 | 13.88, 21.88 | 68.3 | 0.000 | 92 (<0.01) |
| Mixed | 9 | 1.88 | -11.72, 15.48 | 14.1 | 0.758 | 80 (<0.01) |
| Measurement method |  |  |  |  | 0.000 | 91 (<0.01) |
| Manual | 58 | 15.21 | 11.26, 19.17 | 83.3 | 0.000 | 88 (<0.01) |
| Automatic | 5 | 2.60 | 2.44, 2.76 | 2.6 | 0.000 | 0 (1) |
| Not stated | 8 | 30.34 | 17.08, 43.60 | 14.1 | 0.000 | 96 (<0.01) |

Table S11: Meta-analysis comparing the effect of categorical variable experimental factors on WMD in studies assessing necrosis.

| **Experimental factor** | **Analyses (n)** | **R^2^ (%)** | **P-value** | **Beta coefficient** | **95% CI of beta coefficient** |
| --- | --- | --- | --- | --- | --- |
| SYRCLE | 71 | 0.00 | 0.915 | -0.09 | -1.75, 1.57 |
| CAMARADES | 71 | 0.00 | 0.160 | -2.24 | -5.38, 0.90 |
| Start of Tx | 71 | 0.00 | 0.504 | 0.161 | -0.31, 0.64 |
| Tx duration | 71 | 11.1 | 0.541 | -0.07 | -0.28, 0.15 |

Table S12: Meta-regression analyses in studies assessing necrosis. Beta coefficient for natural logarithm of effect size for each variable of interest reflecting unit change. Assessment used residual maximum likelihood (REML) with random effects weighting and Knapp and Hartung t-distribution.

Fibrosis

| **Experimental factor** | **Analyses (n)** | **WMD** | **(95% CI)** | **% Weight** | **P-value** | **I^2^ % (P)** |
| --- | --- | --- | --- | --- | --- | --- |
| Drug Class |  |  |  |  | 0.000 | 90 (<0.01) |
| ACE Inhibitor | 25 | 14.61 | 9.64, 19.58 | 37.9 | 0.000 | 89 (<0.01) |
| ARB | 19 | 7.73 | -20.67, 36.12 | 23.2 | 0.575 | 94 (<0.01) |
| Beta-blocker | 19 | 8.05 | 0.60, 15.50 | 21.7 | 0.036 | 75 (<0.01) |
| Direct renin inhibitor | 2 | 10.55 | 7.37, 13.72 | 3.6 | 0.015 | 0 (0.91) |
| CCB | 5 | 16.54 | 8.96, 24.12 | 8.7 | 0.004 | 67 (0.02) |
| MRA | 3 | 14.50 | -3.73, 32.73 | 4.8 | 0.076 | 66 (0.05) |
| Induction Method |  |  |  |  | 0.000 | 90 (0.01) |
| EMCV | 14 | 7.86 | 2.37, 13.35 | 17.5 | 0.008 | 60 (<0.01) |
| Cardiac myosin | 48 | 13.58 | 5.75, 21.41 | 68.9 | 0.001 | 90 (<0.01) |
| CVB3 | 9 | 7.04 | -2.58, 16.67 | 11.2 | 0.130 | 72 (<0.01) |
| Clozapine | 2 | 39.93 | -76.68, 156.53 | 2.4 | 0.144 | 61 (0.11) |
| Species |  |  |  |  | 0.000 | 90 (<0.01) |
| Mouse | 28 | 9.92 | 5.20, 14.63 | 36.3 | 0.000 | 76 (<0.01) |
| Rat | 45 | 13.79 | 5.04, 22.55 | 63.7 | 0.003 | 91 (<0.01) |
| Sex |  |  |  |  | 0.000 | 90 (<0.01) |
| Female | 8 | 6.66 | 2.04, 11.28 | 9.2 | 0.001 | 0 (0.46) |
| Male | 65 | 13.24 | 7.42, 19.06 | 90.8 | 0.000 | 91 (<0.01) |
| Measurement method |  |  |  |  | 0.047 | 90 (<0.01) |
| Manual | 23 | 13.09 | 5.20, 20.97 | 26.8 | 0.002 | 81 (<0.01) |
| Automatic | 47 | 11.70 | 4.62, 18.78 | 68.5 | 0.002 | 92 (<0.01) |
| Not stated | 3 | 27.09 | 3.83, 50.36 | 4.7 | 0.038 | 77 (0.01) |

Table S13: Meta-analysis comparing the effect of categorical variable experimental factors on WMD in studies assessing fibrosis.

| **Experimental factor** | **Analyses (n)** | **R^2^ (%)** | **P-value** | **Beta coefficient** | **95% CI of beta coefficient** |
| --- | --- | --- | --- | --- | --- |
| SYRCLE | 73 | 0.00 | 0.897 | 0.17 | -2.52, 2.87 |
| CAMARADES | 73 | 7.20 | 0.417 | -2.10 | -7.24, 3.03 |
| Start of Tx | 73 | 0.00 | 0.116 | 0.34 | -0.09, 0.77 |
| Tx duration | 73 | 0.00 | 0.331 | 0.06 | -0.06, -.18 |

Table S14: Meta-regression analyses in studies assessing fibrosis. Beta coefficient for natural logarithm of effect size for each variable of interest reflecting unit change. Assessment used residual maximum likelihood (REML) with random effects weighting and Knapp and Hartung t-distribution

Calcification

| **Experimental factor** | **Analyses (n)** | **WMD** | **(95% CI)** | **% Weight** | **P-value** | **I^2^ % (P)** |
| --- | --- | --- | --- | --- | --- | --- |
| Drug Class |  |  |  |  | 0.039 |  |
| ACE Inhibitor | 13 | 7.37 | 1.11, 13.63 | 52.7 | 0.025 | 58 (<0.01) |
| ARB | 5 | 9.04 | -2.70, 20.78 | 7.3 | 0.099 | 0 (0.68) |
| Beta-blocker | 10 | 0.34 | -6.06, 5.37 | 40.0 | 0.895 | 41 (0.08) |
| Induction Method |  |  |  |  | 0.039 | 50 (<0.01) |
| EMCV | 18 | 5.40 | 2.52, 8.29 | 57.1 | 0.001 | 5 (0.4) |
| CVB3 | 10 | 0.6 | -8.83, 10.04 | 42.9 | 0.888 | 71 (<0.01) |
| Species |  |  |  |  | 0.039 | 50 (<0.01) |
| Mouse | 28 | 4.12 | 0.21, 8.02 | 100.0 | 0.039 | 50 (<0.01) |
| Sex |  |  |  |  | 0.039 |  |
| Female | 9 | 5.08 | 3.46, 6.70 | 39.1 | 0.000 | 0 (0.98) |
| Male | 11 | 6.33 | -2.21, 14.87 | 29.6 | 0.130 | 46 (0.05) |
| Mixed | 8 | -0.13 | -13.62, 13.36 | 31.2 | 0.982 | 77 (<0.01) |
| Measurement method |  |  |  |  | 0.039 | 50 (<0.01) |
| Manual | 24 | 4.15 | -1.34, 9.65 | 68.8 | 0.132 | 55 (<0.01) |
| Automatic | 4 | 4.86 | 2.23, 7.49 | 31.2 | 0.010 | 0 (0.79) |

Table S15: Meta-analysis comparing the effect of categorical variable experimental factors on WMD in studies assessing calcification.

| **Experimental factor** | **Analyses (n)** | **R^2^ (%)** | **P-value** | **Beta coefficient** | **95% CI of beta coefficient** |
| --- | --- | --- | --- | --- | --- |
| SYRCLE | 28 | 0.00 | 0.184 | -1.51 | -3.78, 0.76 |
| CAMARADES | 28 | 0.00 | 0.069 | -6.47 | -13.50, 0.55 |
| Start of Tx | 28 | 0.00 | 0.806 | -0.04 | -0.38, 0.29 |
| Tx duration | 28 | 0.00 | 0.816 | -0.01 | -0.13, 0.11 |

Table S16: Meta-regression analyses in studies assessing calcification. Beta coefficient for natural logarithm of effect size for each variable of interest reflecting unit change. Assessment used residual maximum likelihood (REML) with random effects weighting and Knapp and Hartung t-distribution

Survival

| **Experimental factor** | **Analyses (n)** | **WMD** | **(95% CI)** | **% Weight** | **P-value** | **I^2^ % (P)** |
| --- | --- | --- | --- | --- | --- | --- |
| Drug Class |  |  |  |  | 0.000 | 0 (0.82) |
| ACE Inhibitor | 28 | 0.61 | 0.46, 0.82 | 12.6 | 0.002 | 0 (0.78 |
| ARB | 18 | 0.64 | 0.48, 0.86 | 13.5 | 0.005 | 0 (0.63) |
| Beta-blocker | 39 | 0.58 | 0.49, 0.70 | 34.4 | 0.000 | 0 (0.69) |
| CCB | 10 | 0.62 | 0.36, 1.07 | 2.3 | 0.077 | 0 (0.93) |
| MRA | 4 | 0.83 | 0.54, 1.27 | 37.2 | 0.257 | 29 (0.24) |
| Induction Method |  |  |  |  | 0.000 | 0 (0.82) |
| EMCV | 44 | 0.73 | 0.65, 0.82, | 74.4 | 0.000 | 0 (0.86) |
| Cardiac myosin | 36 | 0.44 | 0.32, 0.61 | 8.4 | 0.000 | 0 (0.94) |
| CVB3 | 19 | 0.64 | 0.43, 0.96 | 17.2 | 0.034 | 28 (0.13) |
| Species |  |  |  |  | 0.000 | 0 (0.82) |
| Mouse | 63 | 0.71 | 0.64, 0.80 | 91.6 | 0.000 | 0 (0.69) |
| Rat | 36 | 0.44 | 0.32, 0.61 | 8.4 | 0.000 | 0 (0.94) |
| Sex |  |  |  |  | 0.000 | 0 (0.82) |
| Female | 13 | 0.49 | 0.28, 0.87 | 1.7 | 0.020 | 0 (0.97) |
| Male | 77 | 0.71 | 0.63, 0.79 | 90.3 | 0.000 | 0 (0.66) |
| Mixed | 9 | 0.60 | 0.25, 1.45 | 8.0 | 0.219 | 36 (0.13) |
| Measurement method |  |  |  |  | 0.000 | 0 (0.82) |
| Manual | 57 | 0.61 | 0.53, 0.71 | 48.3 | 0.000 | 0 (0.93) |
| Automatic | 39 | 0.77 | 0.66, 0.91 | 44.8 | 0.000 | 0 (0.50) |
| Not stated | 3 | 0.57 | 0.12, 2.74 | 6.8 | 0.260 | 67 (0.05) |

Table S17: Meta-analysis comparing the effect of categorical variable experimental factors on WMD in studies assessing survival.

| **Experimental factor** | **Analyses (n)** | **R^2^ (%)** | **P-value** | **Beta coefficient** | **95% CI of beta coefficient** |
| --- | --- | --- | --- | --- | --- |
| SYRCLE | 99 | 0.00 | 0.947 | 0.00 | -0.05, 0.05 |
| CAMARADES | 99 | 0.00 | 0.069 | 0.09 | -0.01, 0.19 |
| Start of Tx | 99 | 0.00 | 0.258 | -0.01 | -0.02, 0.00 |
| Tx duration | 99 | 0.00 | 0.108 | 0.00 | -0.00, 0.01 |

Table S18: Meta-regression analyses in studies assessing survival. Beta coefficient for natural logarithm of effect size for each variable of interest reflecting unit change. Assessment used residual maximum likelihood (REML) with random effects weighting and Knapp and Hartung t-distribution

HW/BW

| **Experimental factor** | **Analyses (n)** | **WMD** | **(95% CI)** | **% Weight** | **P-value** | **I^2^ % (P)** |
| --- | --- | --- | --- | --- | --- | --- |
| Drug Class |  |  |  |  | 0.000 | 67 (<0.01) |
| ACE Inhibitor | 25 | 0.97 | 0.69, 1.26 | 25.2 | 0.000 | 62 (<0.01) |
| ARB | 23 | 0.73 | 0.41, 1.06 | 22.1 | 0.000 | 60 (<0.01) |
| Beta-blocker | 35 | 0.67 | 0.46, 0.87 | 36.4 | 0.000 | 65 (<0.01) |
| Direct renin inhibitor | 2 | 1.30 | -1.83, 4.42 | 2.7 | 0.119 | 13 (0.28) |
| CCB | 7 | 0.85 | 0.2, 1.5 | 9.9 | 0.018 | 82 (<0.01) |
| MRA | 3 | 0.91 | -0.5, 2.32 | 3.7 | 0.109 | 66 (0.05) |
| Induction Method |  |  |  |  | 0.000 | 67 (<0.01) |
| EMCV | 37 | 0.92 | 0.61, 1.23 | 25.5 | 0.000 | 59 (<0.01) |
| Cardiac myosin | 38 | 0.73 | 0.56, 0.90 | 49.1 | 0.000 | 65 (<0.01) |
| CVB3 | 20 | 0.82 | 0.52, 1.11 | 25.4 | 0.000 | 77 (<0.01) |
| Species |  |  |  |  | 0.000 | 67 (<0.01) |
| Mouse | 62 | 0.92 | 0.72, 1.11 | 55.9 | 0.000 | 67 (<0.01) |
| Rat | 33 | 0.67 | 0.49, 0.84 | 44.1 | 0.000 | 64 (<0.01) |
| Sex |  |  |  |  | 0.000 | 67 (<0.01) |
| Female | 18 | 0.77 | 0.25, 1.28 | 11.3 | 0.006 | 55 (<0.01) |
| Male | 73 | 0.78 | 0.64, 0.92 | 84.4 | 0.000 | 68 (<0.01) |
| Mixed | 4 | 1.55 | 1.38, 1.72 | 4.3 | 0.000 | 0 (0.97) |
| Measurement method |  |  |  |  | 0.000 | 67 (<0.01) |
| Manual | 49 | 0.79 | 0.58, 1.00 | 47.8 | 0.000 | 68 (<0.01) |
| Automatic | 36 | 0.73 | 0.54, 0.91 | 43.9 | 0.000 | 64 (<0.01) |
| Not stated | 10 | 1.23 | 0.80, 1.66 | 8.3 | 0.000 | 54 (0.02) |

Table S19: Meta-analysis comparing the effect of categorical variable experimental factors on WMD in studies assessing HW/BW.

| **Experimental factor** | **Analyses (n)** | **R^2^ (%)** | **P-value** | **Beta coefficient** | **95% CI of beta coefficient** |
| --- | --- | --- | --- | --- | --- |
| SYRCLE | 95 | 0.00 | 0.770 | -0.01 | -0.07, 0.05 |
| CAMARADES | 95 | 0.00 | 0.653 | 0.03 | -0.09, 0.15 |
| Start of Tx | 95 | 10.69 | 0.002 | 0.02 | 0.01, 0.03 |
| Tx duration | 95 | 0.00 | 0.363 | 0.00 | -0.00, 0.00 |

Table S20: Meta-regression analyses in studies assessing HW/BW Beta coefficient for natural logarithm of effect size for each variable of interest reflecting unit change. Assessment used residual maximum likelihood (REML) with random effects weighting and Knapp and Hartung t-distribution

Inflammation

| **Experimental factor** | **Analyses (n)** | **WMD** | **(95% CI)** | **% Weight** | **P-value** | **I^2^ % (P)** |
| --- | --- | --- | --- | --- | --- | --- |
|  |  |  |  |  | 0.000 | 88 (<0.01) |
| ACE Inhibitor | 16 | 17.32 | 8.38, 26.25 | 23.3 | 0.000 | 85 (<0.01) |
| ARB | 11 | 19.94 | 8.19, 31.69 | 12.7 | 0.004 | 68 (<0.01) |
| Beta-blocker | 35 | 16.07 | 10.86, 21.28 | 53.1 | 0.000 | 91 (<0.01) |
| Direct renin inhibitor | 2 | 10.21 | 8.94, 11.47 | 3.9 | 0.006 | 0 (0.96) |
| CCB | 5 | 0.68 | -21.03, 19.68 | 7.0 | 0.931 | 70 (0.01) |
| Induction Method |  |  |  |  | 0.000 | 88 (<0.01) |
| EMCV | 36 | 18.92 | 14.1, 23.74 | 48.3 | 0.000 | 69 (<0.01) |
| Cardiac myosin | 6 | 22.09 | 2.21, 41.96 | 8.8 | 0.036 | 89 (<0.01) |
| CVB3 | 27 | 10.22 | 3.74, 16.69 | 42.9 | 0.003 | 93 (<0.01) |
| Species |  |  |  |  | 0.000 | 88 (<0.01) |
| Mouse | 68 | 15.38 | 11.51, 19.25 | 99.8 | 0.000 | 88 (<0.01) |
| Rat | 1 | 42.50 | -23.24, 108.24 | 0.2 | 0.2­05 | N/A |
| Sex |  |  |  |  | 0.000 | 88 (<0.01) |
| Female | 10 | 12.80 | 1.05, 24.54 | 13.3 | 0.036 | 74 (<0.01) |
| Male | 50 | 17.79 | 13.53, 22.06 | 73.4 | 0.000 | 88 (<0.01) |
| Mixed | 9 | 4.49 | -9.15, 18.12 | 13.2 | 0.470 | 86 (<0.01) |
| Measurement method |  |  |  |  | 0.025 | 88 (<0.01) |
| Manual | 56 | 14.05 | 9.82, 18.28 | 80.2 | 0.000 | 87 (<0.01) |
| Automatic | 3 | 10.31 | 4.80, 15.81 | 4.2 | 0.015 | 0 (0.63) |
| Not stated | 10 | 23.82 | 11.47, 36.17 | 15.7 | 0.002 | 94 (<0.01) |

Table S21: Meta-analysis comparing the effect of categorical variable experimental factors on WMD in studies assessing inflammation.

| **Experimental factor** | **Analyses (n)** | **R^2^ (%)** | **P-value** | **Beta coefficient** | **95% CI of beta coefficient** |
| --- | --- | --- | --- | --- | --- |
| SYRCLE | 69 | 0.00 | 0.712 | 0.34 | -1.47, 2.14 |
| CAMARADES | 69 | 0.00 | 0.058 | -2.94 | -5.98, 0.10 |
| Start of Tx | 69 | 2.18 | 0.023 | 0.78 | 0.11, 1.45 |
| Tx duration | 69 | 0.00 | 0.21 | -0.36 | -0.92, 0.21 |

Table S22: Meta-regression analyses in studies assessing inflammation Beta coefficient for natural logarithm of effect size for each variable of interest reflecting unit change. Assessment used residual maximum likelihood (REML) with random effects weighting and Knapp and Hartung t-distribution

Risk of bias

Adapted quality score

To assess study quality, we used a quality score adapted from the CAMARADES checklist in addition to the SYRCLE guidelines to ensure the measure of study quality was appropriate for studies investigating drug therapy in *in vivo* animal models of myocarditis. From the original 10-item CAMARADES score published by Macleod *et al.* in response to a perceived failure of translation of promising neuroprotective agents in stroke,[^22^](#_ENREF_22) we changed two parameters before application to the meta-analysis.

| Original CAMARADES checklist | Adapted CAMARADES checklist |
| --- | --- |
| Use of anaesthetic without significant intrinsic neuroprotective activity | Method of confirmation of myocarditis |
| Blinded application of ischaemia | Blinded application of treatment |

Table S23. Items removed and added from the CAMARADES checklist to make the adapted score used in the study

SYRCLE

| **First Author (year)** | Sequence generation | Baseline characteristics | Allocation concealment | Random housing | Blinding | Random outcome assessment | Blinding | Incomplete outcome data | Selective outcome reporting | Other sources of bias | **Total** |
| --- | --- | --- | --- | --- | --- | --- | --- | --- | --- | --- | --- |
| **Abdel-Wahab 2014** | 0 | 1 | 1 | 1 | 1 | 0 | 1 | 0 | 0 | 0 | 5 |
| **Araki 1995** | 0 | 0 | 1 | 1 | 1 | 0 | 1 | 1 | 1 | 0 | 6 |
| **Baba 2000** | 0 | 1 | 1 | 1 | 1 | 0 | 1 | 1 | 1 | 0 | 7 |
| **Bahk 2007** | 0 | 1 | 1 | 1 | 1 | 0 | 0 | 1 | 1 | 1 | 7 |
| **Chen 2006** | 0 | 0 | 0 | 1 | 0 | 0 | 0 | 1 | 1 | 0 | 3 |
| **Gluck 2010** | 0 | 1 | 0 | 1 | 0 | 0 | 0 | 1 | 1 | 0 | 4 |
| **Godsel 2003** | 0 | 1 | 1 | 1 | 1 | 0 | 1 | 1 | 1 | 1 | 8 |
| **Guo 2009** | 0 | 1 | 1 | 1 | 1 | 0 | 0 | 1 | 1 | 1 | 7 |
| **Juan 2003** | 0 | 1 | 0 | 1 | 0 | 0 | 0 | 1 | 1 | 0 | 4 |
| **Kanda 1995** | 1 | 1 | 0 | 0 | 0 | 1 | 1 | 1 | 1 | 1 | 7 |
| **Kanda 1993** | 1 | 1 | 1 | 1 | 1 | 1 | 0 | 1 | 1 | 1 | 9 |
| **Kashimura 2003** | 0 | 0 | 0 | 1 | 0 | 0 | 0 | 0 | 0 | 0 | 1 |
| **Li 2010** | 1 | 0 | 1 | 1 | 1 | 1 | 1 | 0 | 0 | 1 | 7 |
| **Li 2013** | 0 | 0 | 0 | 1 | 0 | 0 | 1 | 0 | 0 | 0 | 2 |
| **Li-Sha 2013** | 0 | 1 | 0 | 1 | 0 | 0 | 1 | 1 | 1 | 0 | 5 |
| **Liu 2009** | 0 | 0 | 1 | 0 | 1 | 0 | 0 | 0 | 0 | 0 | 2 |
| **Ma 2001** | 0 | 1 | 0 | 1 | 0 | 0 | 0 | 1 | 1 | 0 | 4 |
| **Nishio 2003** | 0 | 1 | 0 | 0 | 0 | 0 | 1 | 1 | 1 | 0 | 4 |
| **Reyes 1988** | 0 | 1 | 1 | 1 | 1 | 0 | 1 | 1 | 1 | 0 | 7 |
| **Rezkalla 1988** | 0 | 1 | 0 | 1 | 0 | 0 | 1 | 1 | 1 | 0 | 5 |
| **Rezkalla 1990(1)** | 0 | 1 | 1 | 1 | 1 | 0 | 1 | 0 | 0 | 0 | 5 |
| **Rezkalla 1990(2)** | 0 | 0 | 0 | 1 | 0 | 0 | 1 | 1 | 1 | 0 | 4 |
| **Saegusa 2007** | 0 | 1 | 1 | 1 | 1 | 0 | 0 | 1 | 1 | 1 | 7 |
| **Shirai 2004** | 0 | 1 | 1 | 1 | 1 | 0 | 0 | 1 | 1 | 1 | 7 |
| **Sukumaran 2010(1)** | 0 | 1 | 0 | 1 | 0 | 0 | 0 | 1 | 1 | 0 | 4 |
| **Sukumaran 2010(2)** | 0 | 1 | 0 | 1 | 0 | 0 | 1 | 1 | 1 | 0 | 5 |
| **Sukumaran 2011(1)** | 0 | 1 | 0 | 1 | 0 | 0 | 0 | 1 | 1 | 0 | 4 |
| **Sukumaran 2011(2)** | 0 | 1 | 0 | 1 | 0 | 0 | 0 | 1 | 1 | 0 | 4 |
| **Sukumaran 2011(3)** | 0 | 1 | 0 | 1 | 0 | 0 | 0 | 1 | 1 | 0 | 4 |
| **Sukumaran 2012(1)** | 0 | 1 | 0 | 1 | 0 | 0 | 0 | 1 | 1 | 0 | 4 |
| **Sukumaran 2012(2)** | 0 | 1 | 1 | 1 | 1 | 0 | 0 | 1 | 1 | 0 | 6 |
| **Suzuki 1993** | 1 | 1 | 1 | 1 | 1 | 1 | 1 | 1 | 1 | 1 | 10 |
| **Tachikawa 2004** | 0 | 1 | 1 | 1 | 1 | 0 | 0 | 1 | 1 | 1 | 7 |
| **Tachikawa 2003** | 0 | 1 | 0 | 1 | 0 | 0 | 0 | 1 | 1 | 0 | 4 |
| **Takada 1997** | 0 | 1 | 1 | 1 | 1 | 0 | 1 | 1 | 1 | 1 | 8 |
| **Takamura 2016** | 0 | 1 | 0 | 1 | 0 | 0 | 0 | 1 | 1 | 0 | 4 |
| **Tanaka 1994** | 0 | 1 | 0 | 1 | 0 | 0 | 1 | 1 | 1 | 0 | 5 |
| **Tominga 1991** | 0 | 1 | 0 | 1 | 0 | 0 | 1 | 1 | 1 | 0 | 5 |
| **Veeraveedu 2006** | 0 | 1 | 1 | 1 | 1 | 0 | 1 | 1 | 1 | 1 | 8 |
| **Wahed 2004** | 0 | 1 | 0 | 1 | 0 | 0 | 0 | 1 | 1 | 0 | 4 |
| **Wahed 2005** | 0 | 1 | 1 | 1 | 1 | 0 | 0 | 1 | 1 | 0 | 6 |
| **Wang 1997** | 1 | 1 | 1 | 0 | 1 | 1 | 1 | 1 | 1 | 1 | 9 |
| **Watanabe 2000** | 0 | 0 | 0 | 1 | 0 | 0 | 1 | 0 | 0 | 0 | 2 |
| **Watanabe 2001** | 0 | 1 | 0 | 1 | 0 | 0 | 0 | 1 | 1 | 0 | 4 |
| **Watanabe 2003 (1)** | 0 | 0 | 0 | 1 | 0 | 0 | 0 | 0 | 0 | 0 | 1 |
| **Watanabe 2003 (2)** | 0 | 1 | 0 | 1 | 0 | 0 | 0 | 1 | 1 | 0 | 4 |
| **Watanabe 2003 (3)** | 0 | 1 | 0 | 1 | 0 | 0 | 0 | 1 | 1 | 0 | 4 |
| **Xiao 2009** | 0 | 1 | 0 | 1 | 0 | 0 | 0 | 1 | 1 | 0 | 4 |
| **Xiao 2009** | 0 | 0 | 0 | 1 | 0 | 0 | 1 | 1 | 1 | 0 | 4 |
| **Xu 1992** | 0 | 1 | 0 | 1 | 0 | 0 | 1 | 1 | 1 | 0 | 5 |
| **Yue-Chun 2008** | 0 | 1 | 1 | 1 | 1 | 0 | 1 | 1 | 1 | 1 | 8 |
| **Yue-Chun 2012** | 0 | 1 | 1 | 1 | 1 | 0 | 1 | 1 | 1 | 1 | 8 |
| **Percentage of papers** | 9 | 79 | 43 | 92 | 43 | 9 | 49 | 83 | 83 | 28 |  |

Table S24. SYRCLE risk of bias tool

CAMARADES

| **First Author (year)** | **Peer reviewed journal** | **Randomisation** | **Blinded assessment of outcomes** | **Co-morbid animals** | **Statement of compliance with regulatory requirements** | **Method of confirmation of myocarditis** | **Statement of control of temperature** | **Sample size calculation** | **Blinded application of treatment** | **Statement of conflict of interest** | **Total** |
| --- | --- | --- | --- | --- | --- | --- | --- | --- | --- | --- | --- |
| **Abdel-Wahab 2014** | 1 | 1 | 1 | 1 | 1 | 1 | 0 | 0 | 0 | 1 | 7 |
| **Araki 1995** | 1 | 1 | 1 | 1 | 0 | 0 | 0 | 0 | 0 | 0 | 4 |
| **Baba 2000** | 1 | 0 | 1 | 1 | 0 | 0 | 0 | 0 | 0 | 0 | 3 |
| **Bahk 2007** | 1 | 0 | 0 | 1 | 0 | 1 | 0 | 0 | 0 | 0 | 3 |
| **Chen 2006** | 1 | 0 | 0 | 1 | 1 | 1 | 0 | 0 | 0 | 0 | 4 |
| **Gluck 2010** | 1 | 0 | 0 | 1 | 0 | 1 | 0 | 0 | 0 | 0 | 3 |
| **Godsel 2003** | 1 | 0 | 1 | 1 | 1 | 1 | 0 | 0 | 0 | 0 | 5 |
| **Guo 2009** | 1 | 0 | 0 | 1 | 1 | 1 | 0 | 0 | 0 | 0 | 4 |
| **Juan 2003** | 1 | 0 | 0 | 1 | 1 | 1 | 0 | 0 | 0 | 0 | 4 |
| **Kanda 1995** | 1 | 1 | 1 | 1 | 0 | 1 | 0 | 0 | 0 | 0 | 5 |
| **Kanda 1993** | 1 | 1 | 0 | 1 | 0 | 1 | 0 | 0 | 0 | 0 | 4 |
| **Kashimura 2003** | 1 | 0 | 0 | 1 | 0 | 1 | 0 | 0 | 0 | 0 | 3 |
| **Li 2010** | 1 | 1 | 1 | 1 | 1 | 1 | 0 | 0 | 1 | 0 | 7 |
| **Li 2013** | 1 | 0 | 1 | 1 | 1 | 1 | 0 | 0 | 1 | 0 | 6 |
| **Li-Sha 2013** | 1 | 0 | 1 | 1 | 1 | 1 | 0 | 0 | 1 | 1 | 7 |
| **Liu 2009** | 1 | 1 | 0 | 1 | 1 | 1 | 0 | 0 | 0 | 0 | 5 |
| **Ma 2001** | 1 | 0 | 0 | 1 | 0 | 1 | 0 | 0 | 0 | 0 | 3 |
| **Nishio 2003** | 1 | 0 | 1 | 1 | 0 | 1 | 0 | 0 | 0 | 0 | 4 |
| **Reyes 1988** | 1 | 1 | 1 | 1 | 0 | 1 | 0 | 0 | 0 | 0 | 5 |
| **Rezkalla 1988** | 1 | 0 | 1 | 1 | 0 | 1 | 0 | 0 | 0 | 0 | 4 |
| **Rezkalla 1990(1)** | 1 | 1 | 1 | 1 | 0 | 1 | 0 | 0 | 0 | 0 | 5 |
| **Rezkalla 1990(2)** | 1 | 0 | 1 | 1 | 0 | 1 | 0 | 0 | 0 | 0 | 4 |
| **Saegusa 2007** | 1 | 1 | 0 | 1 | 0 | 1 | 0 | 0 | 0 | 0 | 4 |
| **Shirai 2004** | 1 | 0 | 0 | 1 | 0 | 1 | 0 | 0 | 0 | 0 | 3 |
| **Sukumaran 2010(1)** | 1 | 0 | 0 | 1 | 0 | 1 | 0 | 0 | 0 | 1 | 4 |
| **Sukumaran 2010(2)** | 1 | 0 | 1 | 1 | 0 | 1 | 0 | 0 | 0 | 1 | 5 |
| **Sukumaran 2011(1)** | 1 | 0 | 0 | 1 | 0 | 1 | 0 | 0 | 0 | 1 | 4 |
| **Sukumaran 2011(2)** | 1 | 0 | 0 | 1 | 1 | 1 | 0 | 0 | 0 | 1 | 5 |
| **Sukumaran 2011(3)** | 1 | 0 | 0 | 1 | 1 | 1 | 0 | 0 | 0 | 1 | 5 |
| **Sukumaran 2012(1)** | 1 | 0 | 0 | 1 | 1 | 1 | 0 | 0 | 0 | 1 | 5 |
| **Sukumaran 2012(2)** | 1 | 0 | 0 | 1 | 1 | 1 | 0 | 0 | 0 | 1 | 5 |
| **Suzuki 1993** | 1 | 1 | 1 | 1 | 0 | 1 | 0 | 0 | 0 | 1 | 6 |
| **Tachikawa 2004** | 1 | 1 | 0 | 1 | 1 | 1 | 0 | 0 | 0 | 1 | 6 |
| **Tachikawa 2003** | 1 | 0 | 0 | 1 | 1 | 1 | 0 | 0 | 0 | 0 | 4 |
| **Takada 1997** | 1 | 1 | 1 | 1 | 0 | 1 | 0 | 0 | 0 | 0 | 5 |
| **Takamura 2016** | 1 | 0 | 0 | 1 | 1 | 1 | 0 | 0 | 0 | 1 | 5 |
| **Tanaka 1994** | 1 | 0 | 1 | 1 | 0 | 1 | 0 | 0 | 0 | 1 | 5 |
| **Tominga 1991** | 1 | 0 | 1 | 1 | 0 | 1 | 0 | 0 | 0 | 0 | 4 |
| **Veeraveedu 2006** | 1 | 0 | 1 | 1 | 0 | 1 | 0 | 0 | 1 | 1 | 6 |
| **Wahed 2004** | 1 | 0 | 0 | 1 | 1 | 1 | 0 | 0 | 0 | 1 | 5 |
| **Wahed 2005** | 1 | 1 | 0 | 1 | 0 | 1 | 0 | 0 | 0 | 1 | 5 |
| **Wang 1997** | 1 | 1 | 1 | 1 | 1 | 1 | 0 | 0 | 1 | 1 | 8 |
| **Watanabe 2000** | 1 | 0 | 1 | 1 | 1 | 1 | 0 | 0 | 0 | 1 | 6 |
| **Watanabe 2001** | 1 | 0 | 0 | 1 | 1 | 1 | 0 | 0 | 0 | 1 | 5 |
| **Watanabe 2003 (1)** | 1 | 0 | 0 | 1 | 1 | 1 | 0 | 0 | 0 | 1 | 5 |
| **Watanabe 2003 (2)** | 1 | 0 | 0 | 1 | 0 | 1 | 0 | 0 | 0 | 1 | 4 |
| **Watanabe 2003 (3)** | 1 | 0 | 0 | 1 | 1 | 1 | 0 | 0 | 0 | 1 | 5 |
| **Xiao 2009** | 1 | 0 | 0 | 1 | 0 | 1 | 0 | 0 | 0 | 0 | 3 |
| **Xiao 2009** | 1 | 0 | 1 | 1 | 1 | 1 | 0 | 0 | 0 | 1 | 6 |
| **Xu 1992** | 1 | 0 | 1 | 1 | 0 | 0 | 0 | 0 | 0 | 0 | 3 |
| **Yue-Chun 2008** | 1 | 0 | 1 | 1 | 0 | 1 | 0 | 0 | 0 | 0 | 4 |
| **Yue-Chun 2012** | 1 | 0 | 1 | 1 | 1 | 1 | 0 | 0 | 0 | 0 | 5 |
| **Percentage of papers** | 100 | 28 | 49 | 100 | 47 | 94 | 0 | 0 | 9 | 43 |  |

Table S25. CAMARADES Checklist

Supplementary figure legends

**Fig S1. Forest plots of meta-analysis of drug efficacy with respect to necrosis, grouped by drug class**

Forest plots of the effect of eligible treatments on necrosis (%), pooled using random-effects meta-analysis. 71 controlled comparisons were included. The diamond represents the pooled difference using a random-effects model. I^2^ is the percentage of total variation across studies due to heterogeneity. Egger’s P<0.001. CI, confidence interval; SD, standard deviation; WMD, weighted mean difference.

**Fig S2. Forest plots of meta-analysis of drug efficacy with respect to fibrosis, grouped by drug class**

Forest plots of the effect of eligible treatments on fibrosis (%), pooled using random-effects meta-analysis. 73 controlled comparisons were included. The diamond represents the pooled difference using a random-effects model. I2 is the percentage of total variation across studies due to heterogeneity. Egger’s P=0.039. CI, confidence interval; SD, standard deviation; WMD, weighted mean difference.

**Fig S3. Forest plots of meta-analysis of drug efficacy with respect to fibrosis, grouped by staining method**

Forest plots of the effect of eligible treatments on fibrosis (%), pooled using random-effects meta-analysis. 73 controlled comparisons were included. The diamond represents the pooled difference using a random-effects model. I2 is the percentage of total variation across studies due to heterogeneity. CI, confidence interval; SD, standard deviation; WMD, weighted mean difference.

**Fig S4. Forest plots of meta-analysis of drug efficacy with respect to calcification, grouped by drug class**

Forest plots of the effect of eligible treatments on calcification (%), pooled using random-effects meta-analysis. 28 controlled comparisons were included. The diamond represents the pooled difference using a random-effects model. I2 is the percentage of total variation across studies due to heterogeneity. Egger’s P=0.422. CI, confidence interval; SD, standard deviation; WMD, weighted mean difference.

**Fig S5. Forest plots of meta-analysis of drug efficacy with respect to survival**

Forest plots of the effect of eligible treatments on survival, pooled using random-effects meta-analysis. 99 controlled comparisons were included. The diamond represents the pooled difference using a random-effects model. I^2^ is the percentage of total variation across studies due to heterogeneity. Egger’s P<0.001. CI, confidence interval; RR, relative risk.

**Fig S6. Forest plots of meta-analysis of drug efficacy with respect to HW/BW**

Forest plots of the effect of eligible treatments on HW/BW, pooled using random-effects meta-analysis. 95 controlled comparisons were included. The diamond represents the pooled difference using a random-effects model. I^2^ is the percentage of total variation across studies due to heterogeneity. Egger’s P=0.012. CI, confidence interval; SD, standard deviation; WMD, weighted mean difference.

**Fig S7. Forest plots of meta-analysis of drug efficacy with respect to inflammation**

Forest plots of the effect of eligible treatments on inflammation (%), pooled using random-effects meta-analysis. 69 controlled comparisons were included. The diamond represents the pooled difference using a random-effects model. I^2^ is the percentage of total variation across studies due to heterogeneity. Egger’s P=0.228. CI, confidence interval; SD, standard deviation; WMD, weighted mean difference.

**Fig S8. Assessment of publication bias for necrosis outcome**

A funnel plot comparing treatment effect to a measure of study size (precision of the effect estimate). The vertical line represents the mean effect size. This plot was assessed visually, with further analysis of publication bias performed using Egger’s and Begg’s regression tests (P<0.001 and P=0.008, respectively).

**Fig S9. Assessment of publication bias for fibrosis outcome**

A funnel plot comparing treatment effect to a measure of study size (precision of the effect estimate). The vertical line represents the mean effect size. This plot was assessed visually, with further analysis of publication bias performed using Egger’s and Begg’s regression tests (P=0.039 and P=0.02, respectively).

**Fig S10. Assessment of publication bias for calcification outcome**

A funnel plot comparing treatment effect to a measure of study size (precision of the effect estimate). The vertical line represents the mean effect size. This plot was assessed visually, with further analysis of publication bias performed using Egger’s and Begg’s regression tests (P=0.422 and P=0.221, respectively).

**Fig S11. Assessment of publication bias for inflammation outcome**

A funnel plot comparing treatment effect to a measure of study size (precision of the effect estimate). The vertical line represents the mean effect size. This plot was assessed visually, with further analysis of publication bias performed using Egger’s and Begg’s regression tests (P=0.228 and P=0.641, respectively).

**Fig S12. Assessment of publication bias for HW/BW outcome**

A funnel plot comparing treatment effect to a measure of study size (precision of the effect estimate). The vertical line represents the mean effect size. This plot was assessed visually, with further analysis of publication bias performed using Egger’s and Begg’s regression tests (P=0.012 and P=0.27, respectively).

**Fig S13. Assessment of publication bias for survival outcome**

A funnel plot comparing treatment effect to a measure of study size (precision of the effect estimate). The vertical line represents the mean effect size. This plot was assessed visually, with further analysis of publication bias performed using Egger’s and Begg’s regression tests (P<0.001 and P=0.297, respectively).

**Fig S14. PRISMA checklist**

**Fig S15. PRISMA flowchart**

References of included studies [^3-5^](#_ENREF_3)^,^[^7-12^](#_ENREF_7)^,^[^15-17^](#_ENREF_15)^,^[^20^](#_ENREF_20)^,^[^23-61^](#_ENREF_23)

Reference List

1. Consumers and Communication Group resources for authors. Cochrane Consumers and Communications Review Group, 2013. (Accessed 12 August, 2015, at <http://cccrg.cochrane.org/author-resources>.)

2. Liberati A, Altman DG, Tetzlaff J, Mulrow C, Gotzsche PC, Ioannidis JP, Clarke M, Devereaux PJ, Kleijnen J, Moher D. The PRISMA statement for reporting systematic reviews and meta-analyses of studies that evaluate healthcare interventions: explanation and elaboration. BMJ 2009;339:b2700.

3. Araki M, Kanda T, Imai S, Suzuki T, Murata K, Kobayashi I. Comparative effects of losartan, captopril, and enalapril on murine acute myocarditis due to encephalomyocarditis virus. J Cardiovasc Pharmacol 1995;26:61-5.

4. Bahk TJ, Daniels MD, Leon JS, Wang K, Engman DM. Comparison of angiotensin converting enzyme inhibition and angiotensin II receptor blockade for the prevention of experimental autoimmune myocarditis. Int J Cardiol 2008;125:85-93.

5. Reyes MP, Khatib R, Khatib G, Ho KL, Smith F, Kloner RA. Prolonged Captopril Therapy in Murine Viral Myocarditis. J Cardiovasc Pharmacol Ther 1998;3:43-50.

6. Dong R, Liu P, Wee L, Butany J, Sole MJ. Verapamil ameliorates the clinical and pathological course of murine myocarditis. J Clin Invest 1992;90:2022-30.

7. Abdel-Wahab BA, Metwally ME, El-khawanki MM, Hashim AM. Protective effect of captopril against clozapine-induced myocarditis in rats: role of oxidative stress, proinflammatory cytokines and DNA damage. Chem Biol Interact 2014;216:43-52.

8. Sukumaran V, Veeraveedu PT, Gurusamy N, Yamaguchi K, Lakshmanan AP, Ma M, Suzuki K, Kodama M, Watanabe K. Cardioprotective effects of telmisartan against heart failure in rats induced by experimental autoimmune myocarditis through the modulation of angiotensin-converting enzyme-2/angiotensin 1-7/mas receptor axis. Int J Biol Sci 2011;7:1077-92.

9. Sukumaran V, Watanabe K, Veeraveedu PT, Gurusamy N, Ma M, Thandavarayan RA, Lakshmanan AP, Yamaguchi K, Suzuki K, Kodama M. Olmesartan, an AT1 antagonist, attenuates oxidative stress, endoplasmic reticulum stress and cardiac inflammatory mediators in rats with heart failure induced by experimental autoimmune myocarditis. Int J Biol Sci 2011;7:154-67.

10. Rezkalla S, Kloner RA, Khatib G, Smith FE, Khatib R. Effect of metoprolol in acute coxsackievirus B3 murine myocarditis. J Am Coll Cardiol 1988;12:412-4.

11. Kashimura T, Hayashi M, Kodama M, Nakazawa M, Abe S, Yoshida T, Tachikawa H, Hanawa H, Kato K, Watanabe K, Aizawa Y. Effects of imidapril and TA-606 on rat dilated cardiomyopathy after myocarditis. Jpn Heart J 2003;44:735-44.

12. Tachikawa H, Kodama M, Hui L, Yoshida T, Hayashi M, Abe S, Kashimura T, Kato K, Hanawa H, Watanabe K, Nakazawa M, Aizawa Y. Angiotensin II type 1 receptor blocker, valsartan, prevented cardiac fibrosis in rat cardiomyopathy after autoimmune myocarditis. J Cardiovasc Pharmacol 2003;41 Suppl 1:S105-10.

13. Kendall MG, Stuart A, Ord JK, Arnold SF, O'Hagan A. Kendall's advanced theory of statistics. 6th ed. London

New York: Edward Arnold ;

Halsted Press; 1994.

14. Elandt-Johnson RC, Johnson NL. Survival models and data analysis. Wiley classics library ed. New York: Wiley; 1999.

15. Xu Y, Yang YZ, Chen HZ, Jin PY, Guo Q, Zhao WZ, Yang JH, Cai QX, Zhou YC. Effect of verapamil on acute coxsackievirus B3 murine myocarditis. Chin Med J (Engl) 1992;105:818-21.

16. Gluck B, Dahlke K, Zell R, Krumbholz A, Decker M, Lehmann J, Wutzler P. Cardioprotective effect of NO-metoprolol in murine coxsackievirus B3-induced myocarditis. J Med Virol 2010;82:2043-52.

17. Rezkalla S, Kloner RA, Khatib G, Khatib R. Effect of delayed captopril therapy on left ventricular mass and myonecrosis during acute coxsackievirus murine myocarditis. Am Heart J 1990;120:1377-81.

18. Wan X, Wang W, Liu J, Tong T. Estimating the sample mean and standard deviation from the sample size, median, range and/or interquartile range. BMC Med Res Methodol 2014;14:135.

19. Sweeting MJ, Sutton AJ, Lambert PC. What to add to nothing? Use and avoidance of continuity corrections in meta-analysis of sparse data. Stat Med 2004;23:1351-75.

20. Yue-Chun L, Teng Z, Na-Dan Z, Li-Sha G, Qin L, Xue-Qiang G, Jia-Feng L. Comparison of effects of ivabradine versus carvedilol in murine model with the Coxsackievirus B3-induced viral myocarditis. PLoS One 2012;7:e39394.

21. Arumugam S, Sreedhar R, Karuppagounder V, Harima M, Nakamura M, Suzuki H, Sone H, Watanabe K. Comparative evaluation of torasemide and spironolactone on adverse cardiac remodeling in a rat model of dilated cardiomyopathy. Cardiovasc Ther 2017;35.

22. Macleod MR, O'Collins T, Howells DW, Donnan GA. Pooling of animal experimental data reveals influence of study design and publication bias. Stroke; a journal of cerebral circulation 2004;35:1203-8.

23. Baba T, Kanda T, Kobayashi I. Reduction of cardiac endothelin-1 by angiotensin II type 1 receptor antagonist in viral myocarditis of mice. Life Sci 2000;67:587-97.

24. Chen XJ, Bian ZP, Lu S, Xu JD, Gu CR, Yang D, Zhang JN. Cardiac protective effect of Astragalus on viral myocarditis mice: comparison with Perindopril. Am J Chin Med 2006;34:493-502.

25. Godsel LM, Leon JS, Wang K, Fornek JL, Molteni A, Engman DM. Captopril prevents experimental autoimmune myocarditis. J Immunol 2003;171:346-52.

26. Guo C, Wang Y, Liang H, Zhang J. ADAMTS-1 contributes to the antifibrotic effect of captopril by accelerating the degradation of type I collagen in chronic viral myocarditis. Eur J Pharmacol 2010;629:104-10.

27. Juan W, Nakazawa M, Watanabe K, Ma M, Wahed MI, Hasegawa G, Naito M, Yamamoto T, Fuse K, Kato K, Kodama M, Aizawa Y. Quinapril inhibits progression of heart failure and fibrosis in rats with dilated cardiomyopathy after myocarditis. Mol Cell Biochem 2003;251:77-82.

28. Kanda T, Inoue M, Suzuki T, Murata K. Low-dose combination therapy with metoprolol and captopril for congestive heart failure in mice. Cardiovasc Drugs Ther 1993;7:795-800.

29. Kanda T, Araki M, Nakano M, Imai S, Suzuki T, Murata K, Kobayashi I. Chronic effect of losartan in a murine model of dilated cardiomyopathy: comparison with captopril. The Journal of pharmacology and experimental therapeutics 1995;273:955-8.

30. Li YC, Ge LS, Yang PL, Tang JF, Lin JF, Chen P, Guan XQ. Carvedilol treatment ameliorates acute coxsackievirus B3-induced myocarditis associated with oxidative stress reduction. Eur J Pharmacol 2010;640:112-6.

31. Li YC, Luo Q, Ge LS, Chen YH, Zhou ND, Zhang T, Guan XQ, Lin JF. Ivabradine inhibits the production of proinflammatory cytokines and inducible nitric oxide synthase in acute coxsackievirus B3-induced myocarditis. Biochem Biophys Res Commun 2013;431:450-5.

32. Li-Sha G, Yi-He C, Na-Dan Z, Teng Z, Yue-Chun L. Effects of carvedilol treatment on cardiac cAMP response element binding protein expression and phosphorylation in acute coxsackievirus B3-induced myocarditis. BMC Cardiovasc Disord 2013;13:100.

33. Liu W, Shimada M, Xiao J, Hu D, Matsumori A. Nifedipine inhibits the activation of inflammatory and immune reactions in viral myocarditis. Life Sci 2009;85:235-40.

34. Ma M, Watanabe K, Wahed MI, Inoue M, Sekiguchi T, Kouda T, Ohta Y, Nakazawa M, Yoshida Y, Yamamoto T, Hanawa H, Kodama M, Fuse K, Aizawa Y. Inhibition of progression of heart failure and expression of TGF-beta 1 mRNA in rats with heart failure by the ACE inhibitor quinapril. J Cardiovasc Pharmacol 2001;38 Suppl 1:S51-4.

35. Nishio R, Shioi T, Sasayama S, Matsumori A. Carvedilol increases the production of interleukin-12 and interferon-gamma and improves the survival of mice infected with the encephalomyocarditis virus. J Am Coll Cardiol 2003;41:340-5.

36. Rezkalla S, Kloner RA, Khatib G, Khatib R. Beneficial effects of captopril in acute coxsackievirus B3 murine myocarditis. Circulation 1990;81:1039-46.

37. Saegusa S, Fei Y, Takahashi T, Sumino H, Moriya J, Kawaura K, Yamakawa J, Itoh T, Morimoto S, Nakahashi T, Iwai K, Matsumoto M, Kanda T. Oral administration of candesartan improves the survival of mice with viral myocarditis through modification of cardiac adiponectin expression. Cardiovasc Drugs Ther 2007;21:155-60.

38. Shirai K, Watanabe K, Ma M, Wahed MI, Inoue M, Saito Y, Suresh PS, Kashimura T, Tachikawa H, Kodama M, Aizawa Y. Effects of angiotensin-II receptor blocker candesartan cilexetil in rats with dilated cardiomyopathy. Mol Cell Biochem 2005;269:137-42.

39. Sukumaran V, Watanabe K, Veeraveedu PT, Ma M, Gurusamy N, Rajavel V, Suzuki K, Yamaguchi K, Kodama M, Aizawa Y. Telmisartan ameliorates experimental autoimmune myocarditis associated with inhibition of inflammation and oxidative stress. Eur J Pharmacol 2011;652:126-35.

40. Sukumaran V, Watanabe K, Veeraveedu PT, Thandavarayan RA, Gurusamy N, Ma M, Yamaguchi K, Suzuki K, Kodama M, Aizawa Y. Beneficial effects of olmesartan, an angiotensin II receptor type 1 antagonist, in rats with dilated cardiomyopathy. Exp Biol Med (Maywood) 2010;235:1338-46.

41. Sukumaran V, Veeraveedu PT, Gurusamy N, Lakshmanan AP, Yamaguchi K, Ma M, Suzuki K, Kodama M, Watanabe K. Telmisartan acts through the modulation of ACE-2/ANG 1-7/mas receptor in rats with dilated cardiomyopathy induced by experimental autoimmune myocarditis. Life Sci 2012;90:289-300.

42. Sukumaran V, Watanabe K, Veeraveedu PT, Thandavarayan RA, Gurusamy N, Ma M, Yamaguchi K, Suzuki K, Kodama M, Aizawa Y. Telmisartan, an angiotensin-II receptor blocker ameliorates cardiac remodeling in rats with dilated cardiomyopathy. Hypertens Res 2010;33:695-702.

43. Sukumaran V, Veeraveedu PT, Gurusamy N, Lakshmanan AP, Yamaguchi K, Ma M, Suzuki K, Nagata M, Takagi R, Kodama M, Watanabe K. Olmesartan attenuates the development of heart failure after experimental autoimmune myocarditis in rats through the modulation of ANG 1-7 mas receptor. Mol Cell Endocrinol 2012;351:208-19.

44. Suzuki H, Matsumori A, Matoba Y, Kyu BS, Tanaka A, Fujita J, Sasayama S. Enhanced expression of superoxide dismutase messenger RNA in viral myocarditis. An SH-dependent reduction of its expression and myocardial injury. J Clin Invest 1993;91:2727-33.

45. Tachikawa H. Fatty Acid Oxidation Is Preserved Regardless of Impaired Uptake in the Chronically Failing Rat Heart. Acta Medica et Biologica 2004;52:1-9.

46. Takada H, Kishimoto C, Hiraoka Y, Kurokawa M, Shiraki K, Sasayama S. Captopril suppresses interstitial fibrin deposition in coxsackievirus B3 myocarditis. Am J Physiol 1997;272:H211-9.

47. Takamura C, Suzuki J, Ogawa M, Watanabe R, Tada Y, Maejima Y, Akazawa H, Komuro I, Isobe M. Suppression of murine autoimmune myocarditis achieved with direct renin inhibition. J Cardiol 2016;68:253-60.

48. Tanaka A, Matsumori A, Wang W, Sasayama S. An angiotensin II receptor antagonist reduces myocardial damage in an animal model of myocarditis. Circulation 1994;90:2051-5.

49. Tominaga M, Matsumori A, Okada I, Yamada T, Kawai C. Beta-blocker treatment of dilated cardiomyopathy. Beneficial effect of carteolol in mice. Circulation 1991;83:2021-8.

50. Veeraveedu PT, Watanabe K, Ma M, Gurusamy N, Palaniyandi SS, Wen J, Prakash P, Wahed MI, Kamal FA, Mito S, Kunisaki M, Kodama M, Aizawa Y. Comparative effects of pranidipine with amlodipine in rats with heart failure. Pharmacology 2006;77:1-10.

51. Wahed MI, Watanabe K, Ma M, Nakazawa M, Takahashi T, Hasegawa G, Naito M, Yamamoto T, Kodama M, Aizawa Y. Effects of pranidipine, a novel calcium channel antagonist, on the progression of left ventricular dysfunction and remodeling in rats with heart failure. Pharmacology 2004;72:26-32.

52. Wahed MI, Watanabe K, Ma M, Yamaguchi K, Takahashi T, Tachikawa H, Kodama M, Aizawa Y. Effects of eplerenone, a selective aldosterone blocker, on the progression of left ventricular dysfunction and remodeling in rats with dilated cardiomyopathy. Pharmacology 2005;73:81-8.

53. Wang WZ, Matsumori A, Yamada T, Shioi T, Okada I, Matsui S, Sato Y, Suzuki H, Shiota K, Sasayama S. Beneficial effects of amlodipine in a murine model of congestive heart failure induced by viral myocarditis. A possible mechanism through inhibition of nitric oxide production. Circulation 1997;95:245-51.

54. Wang JF, Meissner A, Malek S, Chen Y, Ke Q, Zhang J, Chu V, Hampton TG, Crumpacker CS, Abelmann WH, Amende I, Morgan JP. Propranolol ameliorates and epinephrine exacerbates progression of acute and chronic viral myocarditis. Am J Physiol Heart Circ Physiol 2005;289:H1577-83.

55. Watanabe K, Ohta Y, Nakazawa M, Higuchi H, Hasegawa G, Naito M, Fuse K, Ito M, Hirono S, Tanabe N, Hanawa H, Kato K, Kodama M, Aizawa Y. Low dose carvedilol inhibits progression of heart failure in rats with dilated cardiomyopathy. Br J Pharmacol 2000;130:1489-95.

56. Watanabe K, Ohta Y, Inoue M, Ma M, Wahed MI, Nakazawa M, Hasegawa G, Naito M, Fuse K, Ito M, Kato K, Hanawa H, Kodama M, Aizawa Y. Bisoprolol improves survival in rats with heart failure. J Cardiovasc Pharmacol 2001;38 Suppl 1:S55-8.

57. Watanabe K, Juan W, Narasimman G, Ma M, Inoue M, Saito Y, Wahed MI, Nakazawa M, Hasegawa G, Naito M, Tachikawa H, Tanabe N, Kodama M, Aizawa Y, Yamamoto T, Yamaguchi K, Takahashi T. Comparative effects of angiotensin II receptor blockade (candesartan) with angiotensin-converting enzyme inhibitor (quinapril) in rats with dilated cardiomyopathy. J Cardiovasc Pharmacol 2003;41 Suppl 1:S93-7.

58. Watanabe K, Juan W, Narasimman G, Ma M, Inoue M, Saito Y, Wahed MI, Nakazawa M, Hasegawa G, Naito M, Tachikawa H, Tanabe N, Kodama M, Aizawa Y, Yamamoto T, Yamaguchi K, Takahashi T. Betaxolol improves the survival rate and changes natriuretic peptide expression in rats with heart failure. J Cardiovasc Pharmacol 2003;41 Suppl 1:S99-103.

59. Watanabe K, Saito Y, Ma M, Wahed M, Abe Y, Hirabayashi K, Narasimman G, Wen J, Suresh P, Ali F, Shirai K, Soga M, Nagai Y, Nakazawa M, Hasegawa G, Naito M, Tachikawa H, Kodama M, Aizawa Y, Yamaguchi K, Takahashi T. Comparative effects of perindopril with enalapril in rats with dilated cardiomyopathy. J Cardiovasc Pharmacol 2003;42 Suppl 1:S105-9.

60. Xiao J, Shimada M, Liu W, Hu D, Matsumori A. Anti-inflammatory effects of eplerenone on viral myocarditis. Eur J Heart Fail 2009;11:349-53.

61. Yue-Chun L, Li-Sha G, Jiang-Hua R, Peng-Lin Y, Jia-Feng L, Ji-Fei T, Peng C, Zhan-Qiu Y. Protective effects of carvedilol in murine model with the coxsackievirus B3-induced viral myocarditis. J Cardiovasc Pharmacol 2008;51:92-8.
