# Supplementary material for: Therapies to limit myocardial injury in animal models of myocarditis: a systematic review and meta-analysis
Source: Basic Res Cardiol. 2019 Oct 31;114(6):48. doi: 10.1007/s00395-019-0754-x (PMC6823299; doi:10.1007/s00395-019-0754-x)

Necrosis meta-analysis

Study	Intervention Total	Intervention Mean	Intervention SD	Control Total	Control Mean	Control SD	WMD	95% CI	Weight
**Drug Class = ACE inhibitor**									
Araki 1995 – a	6	32.50	12.5	1.20	62.50	15.0	−30.00	[−58.64; −1.36]	0.9%
Araki 1995 – b	6	32.50	12.5	1.20	62.50	15.0	−30.00	[−58.64; −1.36]	0.9%
Baba 2000 – d	6	65.75	17.0	4.60	80.25	15.0	−14.50	[−33.81; 4.81]	1.4%
Baba 2000 – e	16	30.25	7.2	4.60	80.25	15.0	−50.00	[−64.16; −35.84]	1.7%
Chen 2006	18	47.00	31.2	14.00	77.50	21.5	−30.50	[−48.81; −12.19]	1.4%
Godsel 2003	6	12.50	12.5	19.00	27.50	17.5	−15.00	[−27.73; −2.27]	1.8%
Kanda 1993 – c	8	23.20	233.0	2.50	33.00	9.7	−9.80	[−171.71; 152.11]	0.0%
Kanda 1993 – d	4	14.53	250.2	2.50	33.00	9.7	−18.47	[−264.01; 227.07]	0.0%
Kanda 1995 – d	7	25.00	15.0	2.50	45.00	15.0	−20.00	[−41.66; 1.66]	1.2%
Rezkalla 1990(1)	16	2.25	1.4	16.00	4.12	1.6	−1.87	[−2.90; −0.84]	2.4%
Rezkalla 1990(2) – a	15	37.50	17.5	15.00	67.50	17.5	−30.00	[−42.52; −17.48]	1.8%
Rezkalla 1990(2) – b	14	25.00	17.5	14.00	22.50	12.5	2.50	[−8.77; 13.77]	1.9%
Rezkalla 1990(2) – c	15	25.00	27.5	15.00	30.00	27.5	−5.00	[−24.68; 14.68]	1.4%
Suzuki 1993 – a	12	44.00	23.2	4.00	63.00	16.0	−19.00	[−39.47; 1.47]	1.3%
Suzuki 1993 – b	13	33.25	24.5	4.00	63.00	16.0	−29.75	[−50.32; −9.18]	1.3%
Suzuki 1993 – c	12	27.25	21.5	4.00	63.00	16.0	−35.75	[−55.60; −15.90]	1.3%
Takada 1997 – a	20	35.00	17.5	15.00	52.50	25.0	−17.50	[−32.29; −2.71]	1.7%
Takada 1997 – b	44	32.50	15.0	41.00	37.50	20.0	−5.00	[−12.56; 2.56]	2.2%
Overall effect Heterogeneity: $I^2 = 85\%$							−19.40	[−26.71; −12.10]	24.9%
**Drug Class = ARB**									
Araki 1995 – c	7	62.50	15.0	1.20	62.50	15.0	0.00	[−29.05; 29.05]	0.9%
Araki 1995 – d	7	50.00	15.0	1.20	62.50	15.0	−12.50	[−41.55; 16.55]	0.9%
Araki 1995 – e	5	50.00	17.5	1.20	62.50	15.0	−12.50	[−43.41; 18.41]	0.8%
Baba 2000 – a	13	45.25	17.8	4.60	80.25	15.0	−35.00	[−51.76; −18.24]	1.5%
Baba 2000 – b	14	41.00	17.2	4.60	80.25	15.0	−39.25	[−55.67; −22.83]	1.6%
Baba 2000 – c	12	36.25	9.2	4.60	80.25	15.0	−44.00	[−58.67; −29.33]	1.7%
Kanda 1995 – a	8	40.00	12.5	2.50	45.00	15.0	−5.00	[−25.51; 15.51]	1.3%
Kanda 1995 – b	8	25.00	7.5	2.50	45.00	15.0	−20.00	[−39.31; −0.69]	1.4%
Kanda 1995 – c	7	25.00	10.0	2.50	45.00	15.0	−20.00	[−40.02; 0.02]	1.3%
Saegusa 2007	7	37.50	17.5	7.00	60.00	25.0	−22.50	[−45.11; 0.11]	1.2%
Tanaka 1994 – a	10	47.50	17.5	5.00	57.50	30.0	−10.00	[−38.44; 18.44]	0.9%
Tanaka 1994 – b	12	27.50	7.5	5.00	57.50	30.0	−30.00	[−56.64; −3.36]	1.0%
Overall effect Heterogeneity: $I^2 = 45\%$							−23.59	[−32.65; −14.53]	14.5%
**Drug Class = Beta–blocker**									
Kanda 1993 – a	10	28.07	208.2	2.50	33.00	9.7	−4.93	[−134.56; 124.70]	0.1%
Kanda 1993 – b	10	25.48	275.2	2.50	33.00	9.7	−7.52	[−178.54; 163.50]	0.0%
Li 2010 – a	24	27.50	5.5	7.00	57.25	3.5	−29.75	[−33.15; −26.35]	2.4%
Li 2010 – b	13	54.50	3.2	7.00	57.25	3.5	−2.75	[−5.89; 0.39]	2.4%
Li 2013 – a	8	26.75	48.1	8.00	45.75	45.2	−19.00	[−64.75; 26.75]	0.5%
Li 2013 – b	8	42.00	62.9	8.00	67.50	36.8	−25.50	[−76.01; 25.01]	0.4%
Li–Sha 2013 – a	8	36.25	46.7	8.00	43.75	41.7	−7.50	[−50.88; 35.88]	0.5%
Li–Sha 2013 – b	8	28.25	24.8	8.00	59.50	36.8	−31.25	[−61.96; −0.54]	0.8%
Nishio 2003 – a	5	40.00	11.2	2.50	60.00	11.9	−20.00	[−37.67; −2.33]	1.5%
Nishio 2003 – b	5	27.50	11.2	2.50	60.00	11.9	−32.50	[−50.17; −14.83]	1.5%
Nishio 2003 – c	5	27.50	5.6	1.67	55.00	3.2	−27.50	[−34.43; −20.57]	2.2%
Nishio 2003 – d	5	60.00	33.5	1.67	55.00	3.2	5.00	[−24.80; 34.80]	0.9%
Nishio 2003 – e	5	32.50	5.6	1.67	55.00	3.2	−22.50	[−29.43; −15.57]	2.2%
Rezkalla 1988 – a	10	2.50	7.9	10.00	0.00	0.0	2.50	[−2.40; 7.40]	2.3%
Rezkalla 1988 – b	10	62.50	31.6	10.00	55.00	31.6	7.50	[−20.22; 35.22]	0.9%
Rezkalla 1988 – c	10	57.50	31.6	10.00	47.50	31.6	10.00	[−17.72; 37.72]	0.9%
Rezkalla 1988 – d	20	52.50	44.7	20.00	10.00	33.5	42.50	[18.00; 67.00]	1.1%
Tominga 1991 – a	7	37.50	20.0	3.00	37.50	17.5	0.00	[−24.73; 24.73]	1.1%
Tominga 1991 – b	7	30.00	25.0	3.00	37.50	17.5	−7.50	[−34.61; 19.61]	1.0%
Wang 2005 – a	5	22.50	5.6	1.67	47.50	6.5	−25.00	[−35.95; −14.05]	2.0%
Wang 2005 – b	5	20.00	5.6	1.67	47.50	6.5	−27.50	[−38.45; −16.55]	2.0%
Wang 2005 – c	5	22.50	16.8	1.67	47.50	6.5	−25.00	[−42.67; −7.33]	1.5%
Wang 2005 – d	5	25.00	16.8	1.67	57.50	9.7	−32.50	[−53.29; −11.71]	1.3%
Wang 2005 – e	5	22.50	11.2	1.67	57.50	9.7	−35.00	[−52.66; −17.34]	1.5%
Wang 2005 – f	5	32.50	11.2	1.67	57.50	9.7	−25.00	[−42.66; −7.34]	1.5%
Wang 2005 – g	5	20.00	5.6	1.67	52.50	9.7	−32.50	[−47.99; −17.01]	1.6%
Wang 2005 – h	5	27.50	11.2	1.67	52.50	9.7	−25.00	[−42.66; −7.34]	1.5%
Wang 2005 – i	5	27.50	16.8	1.67	52.50	9.7	−25.00	[−45.79; −4.21]	1.3%
Yue–Chun 2008 – a	6	23.00	4.0	3.00	47.75	4.8	−24.75	[−31.01; −18.49]	2.3%
Yue–Chun 2008 – b	6	45.25	3.5	3.00	47.75	4.8	−2.50	[−8.56; 3.56]	2.3%
Yue–Chun 2008 – c	6	30.75	3.5	3.00	60.75	3.0	−30.00	[−34.40; −25.60]	2.4%
Yue–Chun 2008 – d	6	56.75	5.2	3.00	60.75	3.0	−4.00	[−9.40; 1.40]	2.3%
Yue–Chun 2012 – a	8	18.75	11.3	8.00	25.00	13.4	−6.25	[−18.42; 5.92]	1.9%
Yue–Chun 2012 – b	8	27.50	9.2	8.00	47.00	9.2	−19.50	[−28.51; −10.49]	2.1%
Yue–Chun 2012 – c	8	31.25	11.3	8.00	59.50	12.7	−28.25	[−40.05; −16.45]	1.9%
Overall effect Heterogeneity: $I^2 = 90\%$							−16.98	[−22.49; −11.47]	51.8%
**Drug Class = CCB**									
Liu 2009	10	1.20	1.3	10.00	3.80	1.8	−2.60	[−3.98; −1.22]	2.4%
Wang 1997 – a	10	45.00	40.0	5.00	55.00	35.0	−10.00	[−49.44; 29.44]	0.6%
Wang 1997 – b	10	28.25	20.0	5.00	55.00	35.0	−26.75	[−59.84; 6.34]	0.7%
Xu 1992 – a	10	10.00	17.5	9.00	5.00	15.0	5.00	[−9.62; 19.62]	1.7%
Xu 1992 – b	13	30.00	15.0	14.00	20.00	25.0	10.00	[−5.43; 25.43]	1.6%
Xu 1992 – c	10	12.50	17.5	9.00	5.00	10.0	7.50	[−5.16; 20.16]	1.8%
Overall effect Heterogeneity: $I^2 = 38\%$							0.93	[−7.98; 9.83]	8.9%
**Overall effect** Heterogeneity: $I^2 = 91\%$							**−16.91**	**[−20.65; −13.17]**	**100.0%**

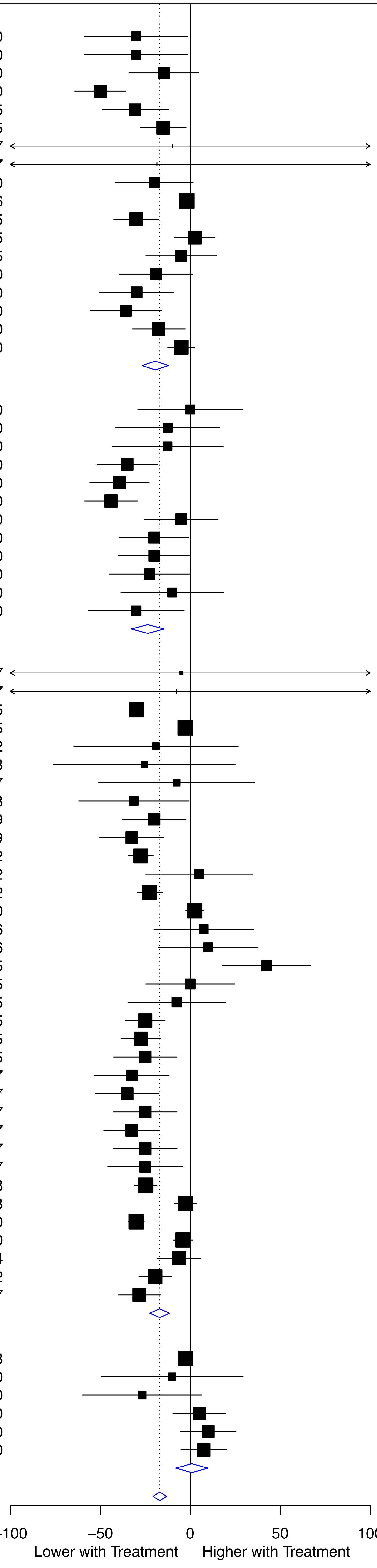

Supplement: Supplementary file 2 — Supplementary material 2 (PDF 343 kb) [file 395_2019_754_MOESM2_ESM.pdf]
