# Supplementary material for: Therapies to limit myocardial injury in animal models of myocarditis: a systematic review and meta-analysis
Source: Basic Res Cardiol. 2019 Oct 31;114(6):48. doi: 10.1007/s00395-019-0754-x (PMC6823299; doi:10.1007/s00395-019-0754-x)

Fibrosis meta-analysis

Study	Intervention Total	Intervention Mean	Intervention SD	Control Total	Control Mean	Control SD	WMD	95% CI	Weight
**Drug Class = ACE inhibitor**									
Abdel–Wahab 2014 – a	10	19.00	13.4	5.00	49.25	16.8	−30.25	[−47.15; −13.35]	1.1%
Abdel–Wahab 2014 – b	10	0.62	1.4	5.00	49.25	16.8	−48.63	[−63.36; −33.90]	1.3%
Bahk 2007 – b	18	7.00	11.7	10.00	43.75	15.0	−36.75	[−47.51; −25.99]	1.5%
Godsel 2003	6	20.00	20.0	19.00	32.50	15.0	−12.50	[−29.87; 4.87]	1.1%
Guo 2009	18	9.43	1.2	18.00	14.88	1.1	−5.45	[−6.20; −4.70]	2.0%
Juan 2003 – a	9	22.00	12.0	3.67	32.00	7.7	−10.00	[−21.09; 1.09]	1.5%
Juan 2003 – b	11	13.00	9.9	3.67	32.00	7.7	−19.00	[−28.80; −9.20]	1.6%
Juan 2003 – c	11	6.00	3.3	3.67	32.00	7.7	−26.00	[−34.08; −17.92]	1.7%
Kanda 1993 – c	8	12.48	5.8	2.50	18.61	5.7	−6.13	[−14.28; 2.02]	1.7%
Kanda 1993 – d	4	8.37	3.2	2.50	18.61	5.7	−10.24	[−17.96; −2.52]	1.7%
Kanda 1995 – d	7	32.50	12.5	2.50	67.50	20.0	−35.00	[−61.46; −8.54]	0.7%
Kashimura 2003 – a	7	10.70	4.3	2.00	12.40	1.8	−1.70	[−5.74; 2.34]	1.9%
Kashimura 2003 – b	5	8.87	3.2	2.00	12.40	1.8	−3.53	[−7.28; 0.22]	1.9%
Ma 2001	8	6.00	212.1	8.00	29.00	17.0	−23.00	[−170.47; 124.47]	0.0%
Reyes 1998 – a	50	3.00	8.8	50.00	8.75	15.9	−5.75	[−10.79; −0.71]	1.9%
Reyes 1998 – b	25	7.00	11.2	25.00	9.00	13.8	−2.00	[−8.96; 4.96]	1.8%
Tachikawa 2004	8	19.00	19.8	8.00	29.00	14.1	−10.00	[−26.86; 6.86]	1.1%
Takada 1997 – a	20	20.00	12.5	15.00	30.00	20.0	−10.00	[−21.51; 1.51]	1.5%
Takada 1997 – b	44	30.00	12.5	41.00	35.00	15.0	−5.00	[−10.89; 0.89]	1.8%
Watanabe 2003(1) – a	14	9.00	11.2	5.50	36.00	14.1	−27.00	[−40.15; −13.85]	1.4%
Watanabe 2003(3) – a	13	26.50	9.0	2.20	36.00	3.9	−9.50	[−16.57; −2.43]	1.8%
Watanabe 2003(3) – b	15	15.00	8.1	2.20	36.00	3.9	−21.00	[−27.55; −14.45]	1.8%
Watanabe 2003(3) – c	15	7.50	5.4	2.20	36.00	3.9	−28.50	[−34.29; −22.71]	1.8%
Watanabe 2003(3) – d	13	29.50	12.3	2.20	36.00	3.9	−6.50	[−14.89; 1.89]	1.7%
Watanabe 2003(3) – e	15	15.60	7.8	2.20	36.00	3.9	−20.40	[−26.83; −13.97]	1.8%
Overall effect	Heterogeneity: $I^2 = 89\%$						−14.61	[−19.58; −9.64]	37.9%
**Drug Class = ARB**									
Bahk 2007 – a	19	15.75	17.4	10.00	43.75	15.0	−28.00	[−40.17; −15.83]	1.4%
Kanda 1995 – a	8	67.50	20.0	2.50	67.50	20.0	0.00	[−28.40; 28.40]	0.6%
Kanda 1995 – b	8	55.00	20.0	2.50	67.50	20.0	−12.50	[−40.90; 15.90]	0.6%
Kanda 1995 – c	7	65.00	27.5	2.50	67.50	20.0	−2.50	[−34.59; 29.59]	0.5%
Kashimura 2003 – c	6	7.71	4.9	2.00	12.40	1.8	−4.69	[−9.34; −0.04]	1.9%
Kashimura 2003 – d	6	10.09	5.5	2.00	12.40	1.8	−2.31	[−7.42; 2.80]	1.9%
Shirai 2004 – a	11	625.00	223.9	3.67	32.00	7.1	593.00	[460.51; 725.49]	0.0%
Shirai 2004 – b	13	500.00	270.4	3.67	32.00	7.1	468.00	[320.82; 615.18]	0.0%
Shirai 2004 – c	14	300.00	131.0	3.67	32.00	7.1	268.00	[199.02; 336.98]	0.1%
Sukumaran 2010(1)	10	17.50	10.1	10.00	33.80	4.1	−16.30	[−23.07; −9.53]	1.8%
Sukumaran 2010(2)	9	15.00	6.0	6.00	33.80	3.2	−18.80	[−23.47; −14.13]	1.9%
Sukumaran 2011(1)	8	32.50	5.9	8.00	56.75	6.8	−24.25	[−30.50; −18.00]	1.8%
Sukumaran 2011(2)	8	27.50	4.5	8.00	56.75	6.8	−29.25	[−34.91; −23.59]	1.8%
Sukumaran 2011(3)	6	35.50	5.1	6.00	54.00	5.9	−18.50	[−24.75; −12.25]	1.8%
Sukumaran 2012(1)	6	27.20	6.9	6.00	60.50	7.3	−33.30	[−41.34; −25.26]	1.7%
Sukumaran 2012(2)	6	18.75	6.1	8.00	35.50	3.7	−16.75	[−22.27; −11.23]	1.8%
Tachikawa 2003 – a	8	31.90	18.7	4.00	34.20	13.4	−2.30	[−20.73; 16.13]	1.0%
Tachikawa 2003 – b	8	25.60	15.3	4.00	34.20	13.4	−8.60	[−25.46; 8.26]	1.1%
Watanabe 2003(1) – b	12	26.00	13.9	5.50	36.00	14.1	−10.00	[−24.13; 4.13]	1.3%
Overall effect	Heterogeneity: $I^2 = 94\%$						−7.73	[−36.12; 20.67]	23.2%
**Drug Class = Beta–blocker**									
Gluck 2010 – a	19	25.00	20.0	22.00	50.00	19.8	−25.00	[−37.23; −12.77]	1.4%
Gluck 2010 – b	8	39.58	22.3	7.00	62.50	28.7	−22.92	[−49.22; 3.38]	0.7%
Kanda 1993 – a	10	15.07	6.6	2.50	18.61	5.7	−3.54	[−11.72; 4.64]	1.7%
Kanda 1993 – b	10	14.92	7.7	2.50	18.61	5.7	−3.69	[−12.23; 4.85]	1.7%
Li 2013 – b	8	133.25	116.0	8.00	14.40	11.1	118.85	[38.12; 199.58]	0.1%
Li–Sha 2013 – b	8	157.75	172.5	8.00	14.41	10.3	143.34	[23.57; 263.11]	0.1%
Tominga 1991 – c	10	42.50	22.5	12.00	47.50	0.9	−5.00	[−18.95; 8.95]	1.3%
Tominga 1991 – d	14	55.00	15.0	12.00	47.50	0.9	7.50	[−0.37; 15.37]	1.7%
Tominga 1991 – e	7	52.50	27.5	6.67	52.50	0.9	0.00	[−20.38; 20.38]	0.9%
Tominga 1991 – f	10	35.00	12.5	6.67	52.50	0.9	−17.50	[−25.28; −9.72]	1.7%
Tominga 1991 – g	6	30.00	20.0	6.67	52.50	0.9	−22.50	[−38.52; −6.48]	1.2%
Watanabe 2000 – a	10	12.00	3.2	5.00	31.00	4.5	−19.00	[−23.38; −14.62]	1.9%
Watanabe 2000 – b	10	24.00	12.7	5.00	31.00	4.5	−7.00	[−15.76; 1.76]	1.6%
Watanabe 2001 – a	9	44.00	15.0	2.67	42.00	9.8	2.00	[−13.30; 17.30]	1.2%
Watanabe 2001 – b	13	37.00	21.6	2.67	42.00	9.8	−5.00	[−21.63; 11.63]	1.1%
Watanabe 2001 – c	13	35.00	25.2	2.67	42.00	9.8	−7.00	[−25.07; 11.07]	1.1%
Watanabe 2003(2) – a	10	31.00	28.5	2.67	38.00	14.7	−7.00	[−31.94; 17.94]	0.7%
Watanabe 2003(2) – b	13	27.00	28.8	2.67	38.00	14.7	−11.00	[−34.59; 12.59]	0.8%
Watanabe 2003(2) – c	13	25.00	32.5	2.67	38.00	14.7	−13.00	[−37.94; 11.94]	0.7%
Overall effect	Heterogeneity: $I^2 = 75\%$						−8.05	[−15.50; −0.60]	21.7%
**Drug Class = Direct renin inhibitor**									
Takamura 2016 – a	10	7.30	3.8	5.00	17.60	6.3	−10.30	[−16.27; −4.33]	1.8%
Takamura 2016 – b	6	6.80	3.2	5.00	17.60	6.3	−10.80	[−16.85; −4.75]	1.8%
Overall effect	Heterogeneity: $I^2 = 0\%$						−10.55	[−13.72; −7.37]	3.6%
**Drug Class = CCB**									
Veeraveedu 2006 – a	9	29.00	15.0	2.67	36.00	3.3	−7.00	[−17.56; 3.56]	1.5%
Veeraveedu 2006 – b	10	19.00	12.7	2.67	36.00	3.3	−17.00	[−25.77; −8.23]	1.6%
Veeraveedu 2006 – c	10	13.00	6.3	2.67	36.00	3.3	−23.00	[−28.54; −17.46]	1.8%
Wahed 2004 – a	8	24.00	5.7	5.00	36.00	4.5	−12.00	[−17.54; −6.46]	1.8%
Wahed 2004 – b	10	16.00	6.3	5.00	36.00	4.5	−20.00	[−25.54; −14.46]	1.8%
Overall effect	Heterogeneity: $I^2 = 67\%$						−16.54	[−24.12; −8.96]	8.7%
**Drug Class = MRA**									
Wahed 2005 – a	12	27.00	8.7	5.00	35.00	6.7	−8.00	[−15.66; −0.34]	1.7%
Wahed 2005 – b	15	13.00	11.6	5.00	35.00	6.7	−22.00	[−30.32; −13.68]	1.7%
Xiao 2009 – a	14	19.80	9.7	3.00	33.40	9.3	−13.60	[−25.34; −1.86]	1.5%
Overall effect	Heterogeneity: $I^2 = 66\%$						−14.50	[−32.73; 3.73]	4.8%
**Overall effect**	Heterogeneity: $I^2 = 90\%$						**−12.80**	**[−17.98; −7.62]**	**100.0%**

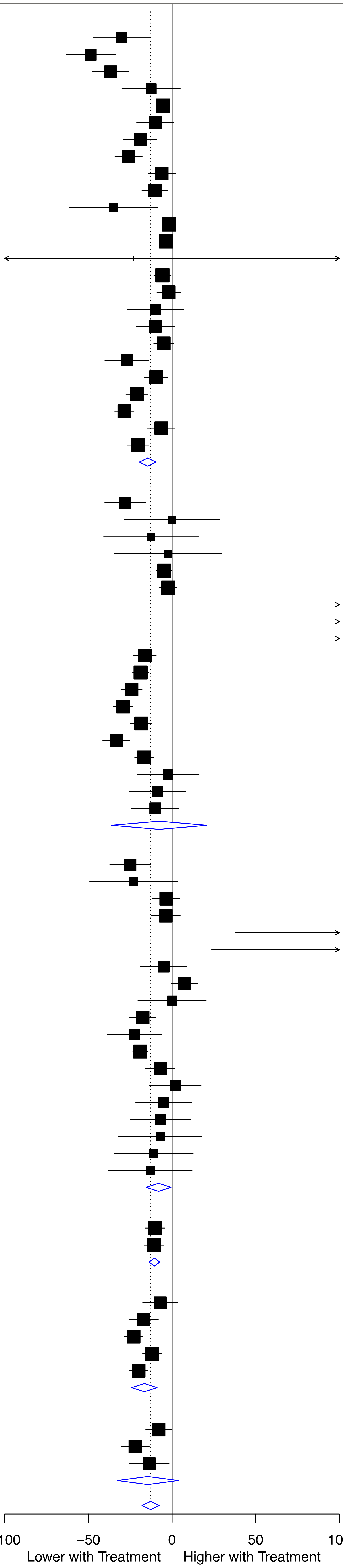

Supplement: Supplementary file 3 — Supplementary material 3 (PDF 355 kb) [file 395_2019_754_MOESM3_ESM.pdf]
