# Supplementary figures and images for: Therapies to limit myocardial injury in animal models of myocarditis: a systematic review and meta-analysis
Source: Basic Res Cardiol. 2019 Oct 31;114(6):48. doi: 10.1007/s00395-019-0754-x (PMC6823299; doi:10.1007/s00395-019-0754-x)

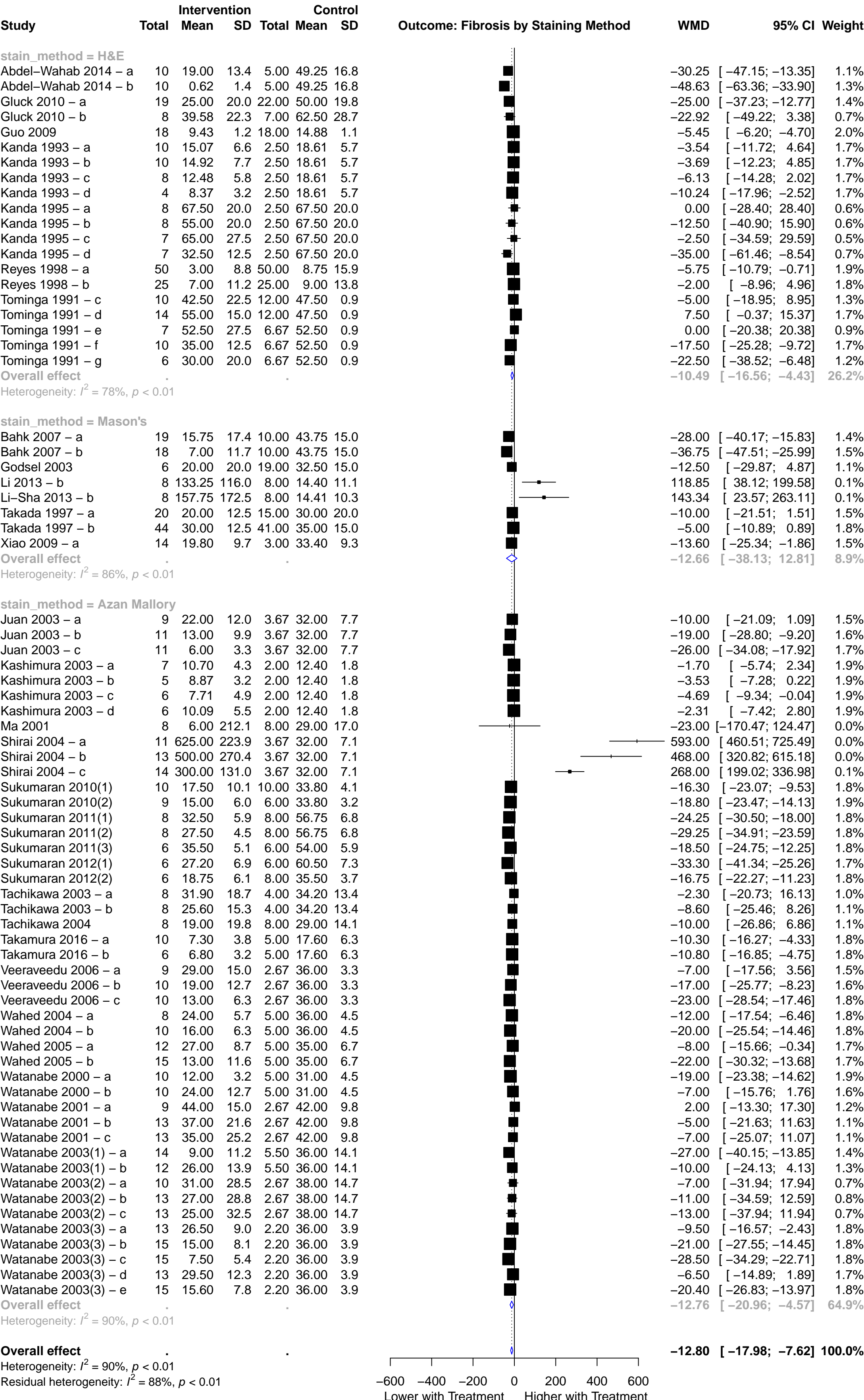

Supplement: Supplementary file 4 — Supplementary material 4 (PDF 13 kb) [file 395_2019_754_MOESM4_ESM.pdf]

Calcification meta-analysis

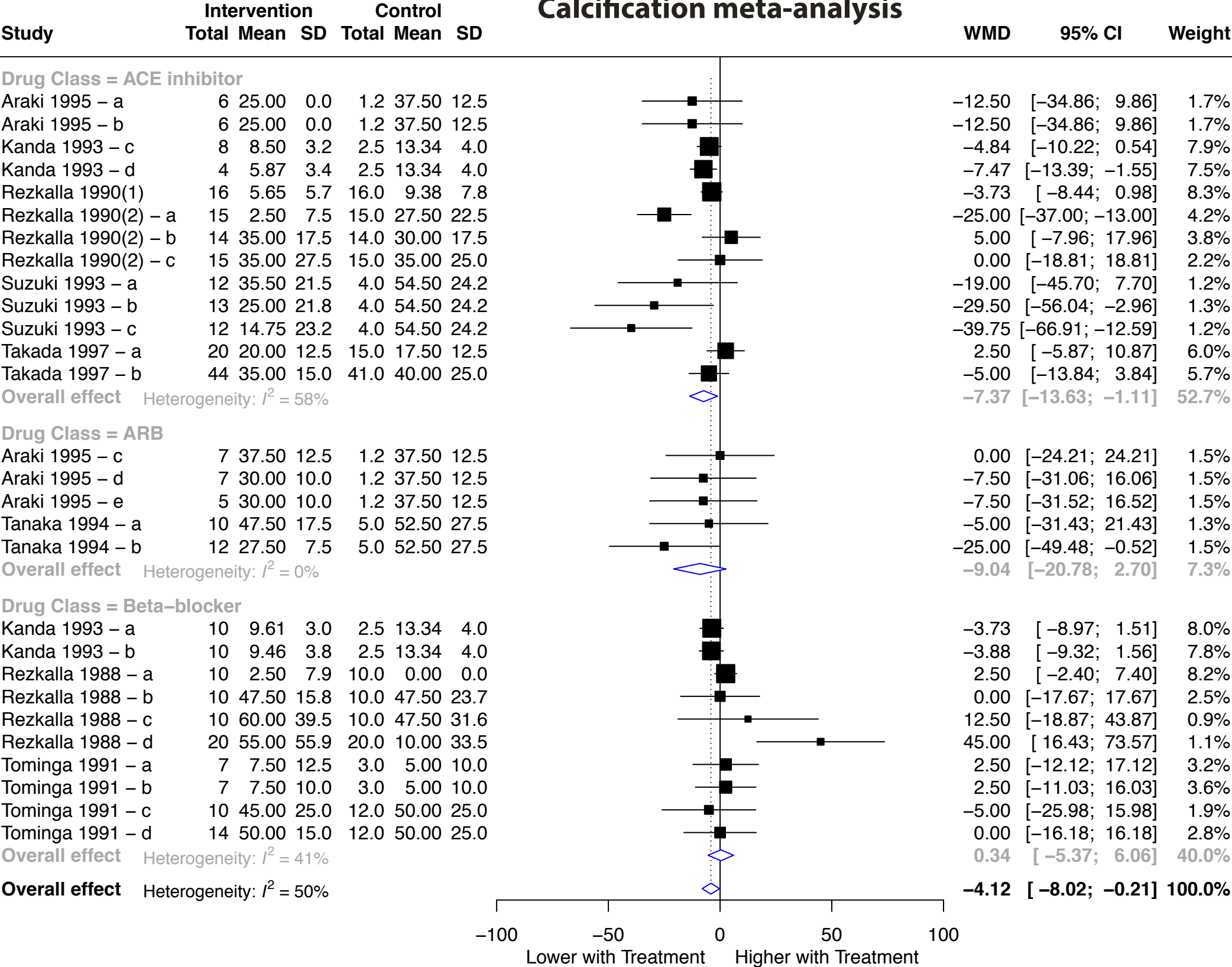

Supplement: Supplementary file 5 — Supplementary material 5 (PDF 262 kb) [file 395_2019_754_MOESM5_ESM.pdf]

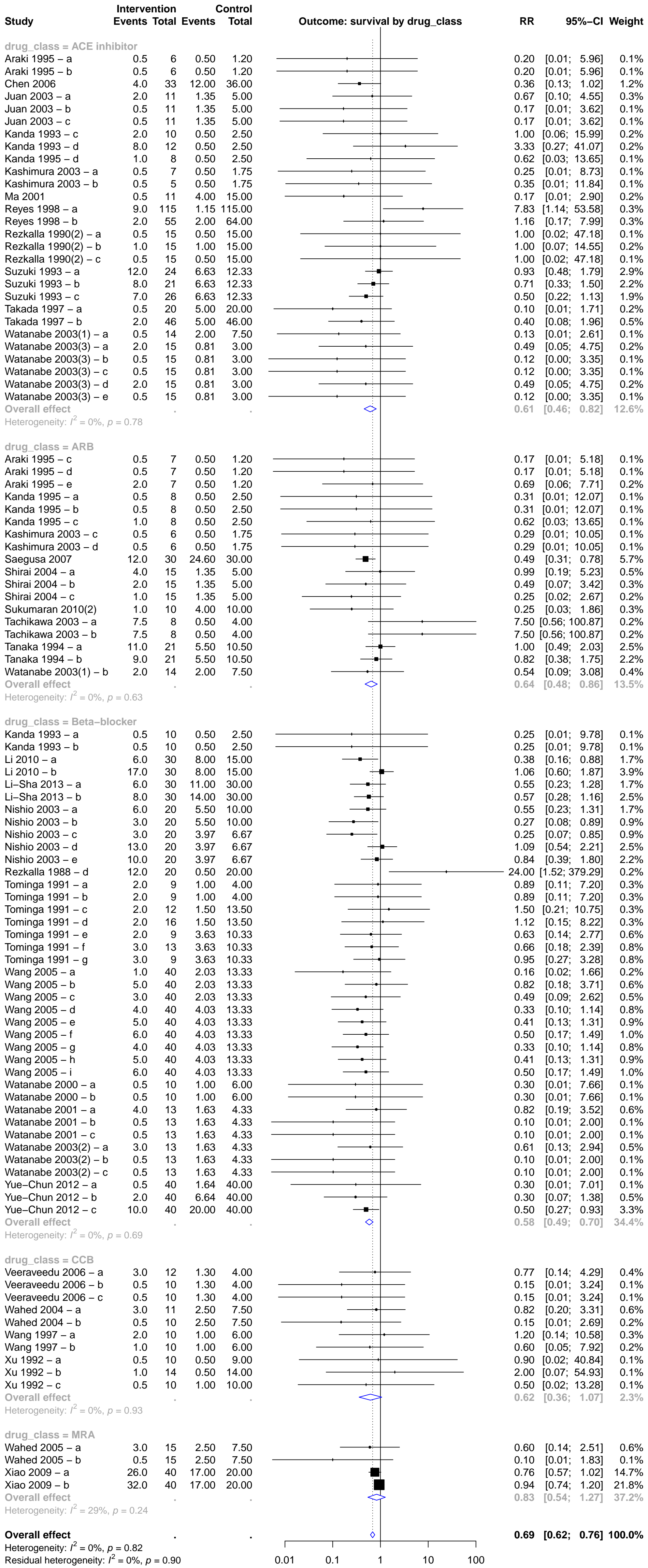

Supplement: Supplementary file 6 — Supplementary material 6 (PDF 14 kb) [file 395_2019_754_MOESM6_ESM.pdf]

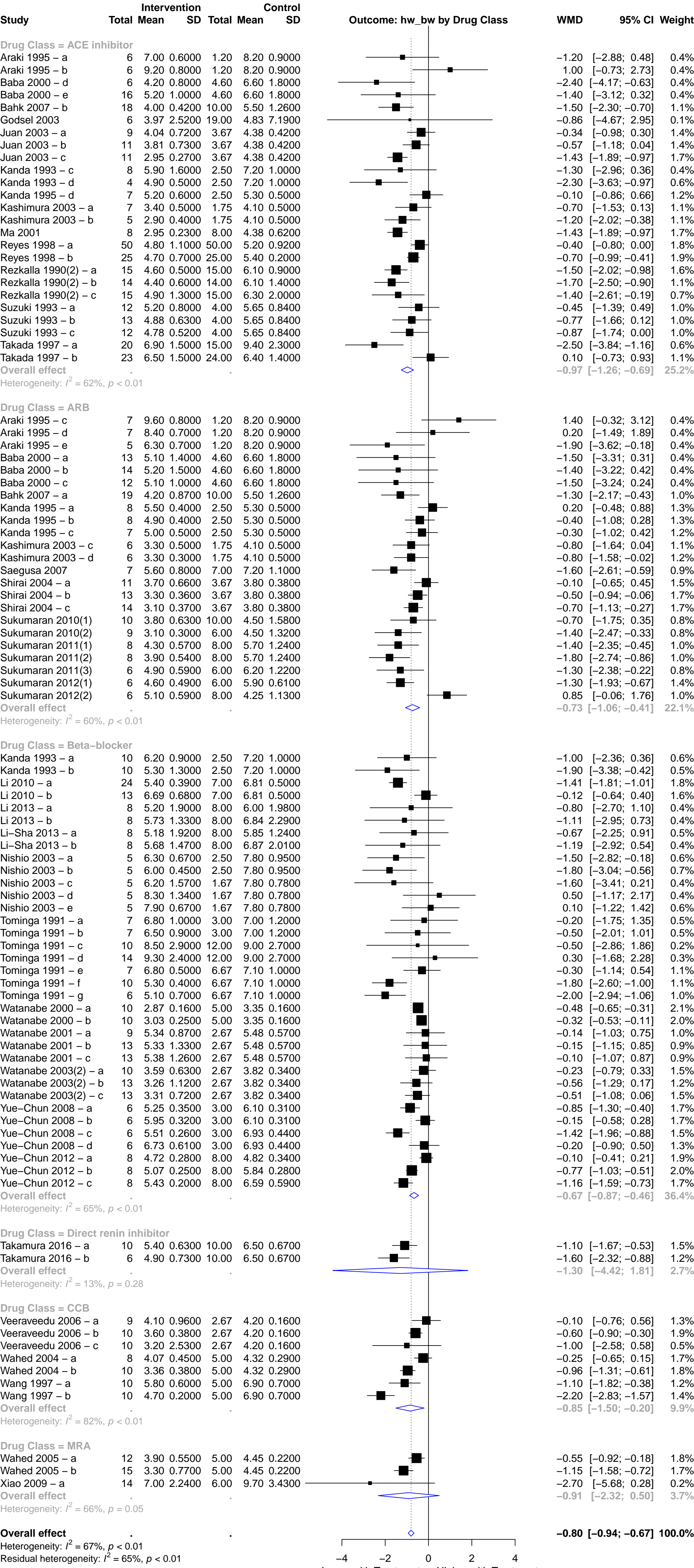

Supplement: Supplementary file 7 — Supplementary material 7 (PDF 15 kb) [file 395_2019_754_MOESM7_ESM.pdf]

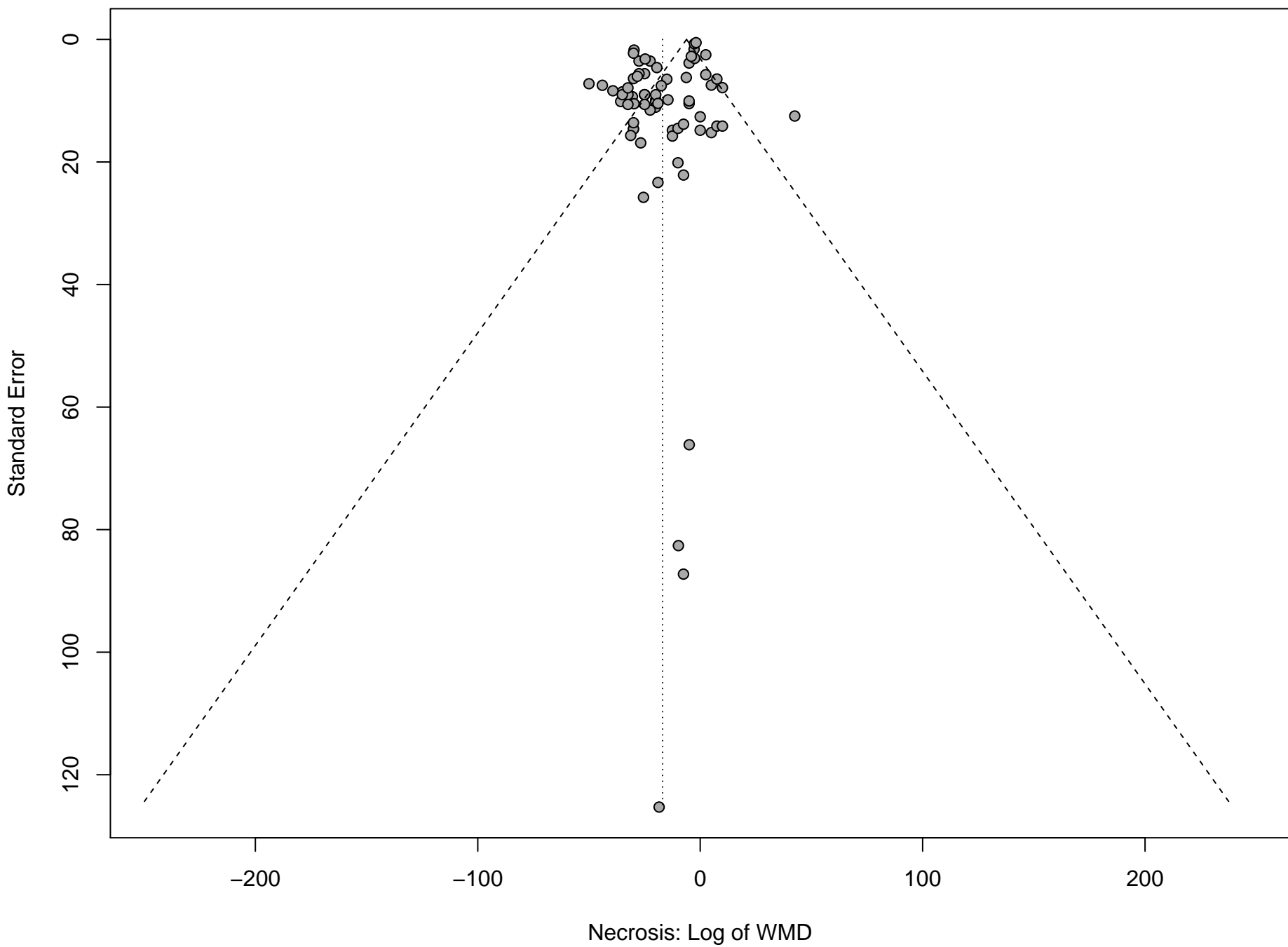

Supplement: Supplementary file 9 — Supplementary material 9 (PDF 10 kb) [file 395_2019_754_MOESM9_ESM.pdf]

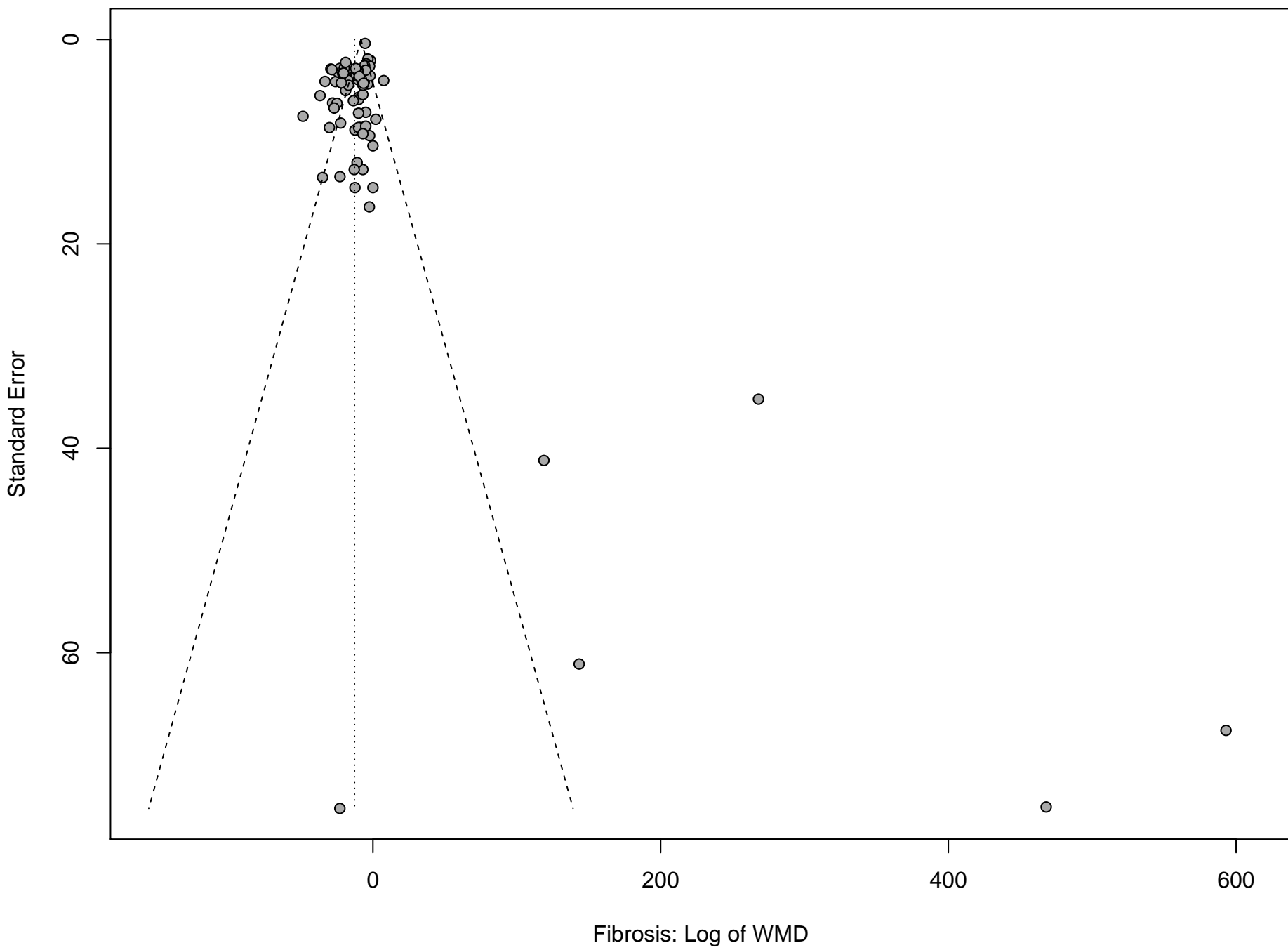

Supplement: Supplementary file 10 — Supplementary material 10 (PDF 10 kb) [file 395_2019_754_MOESM10_ESM.pdf]

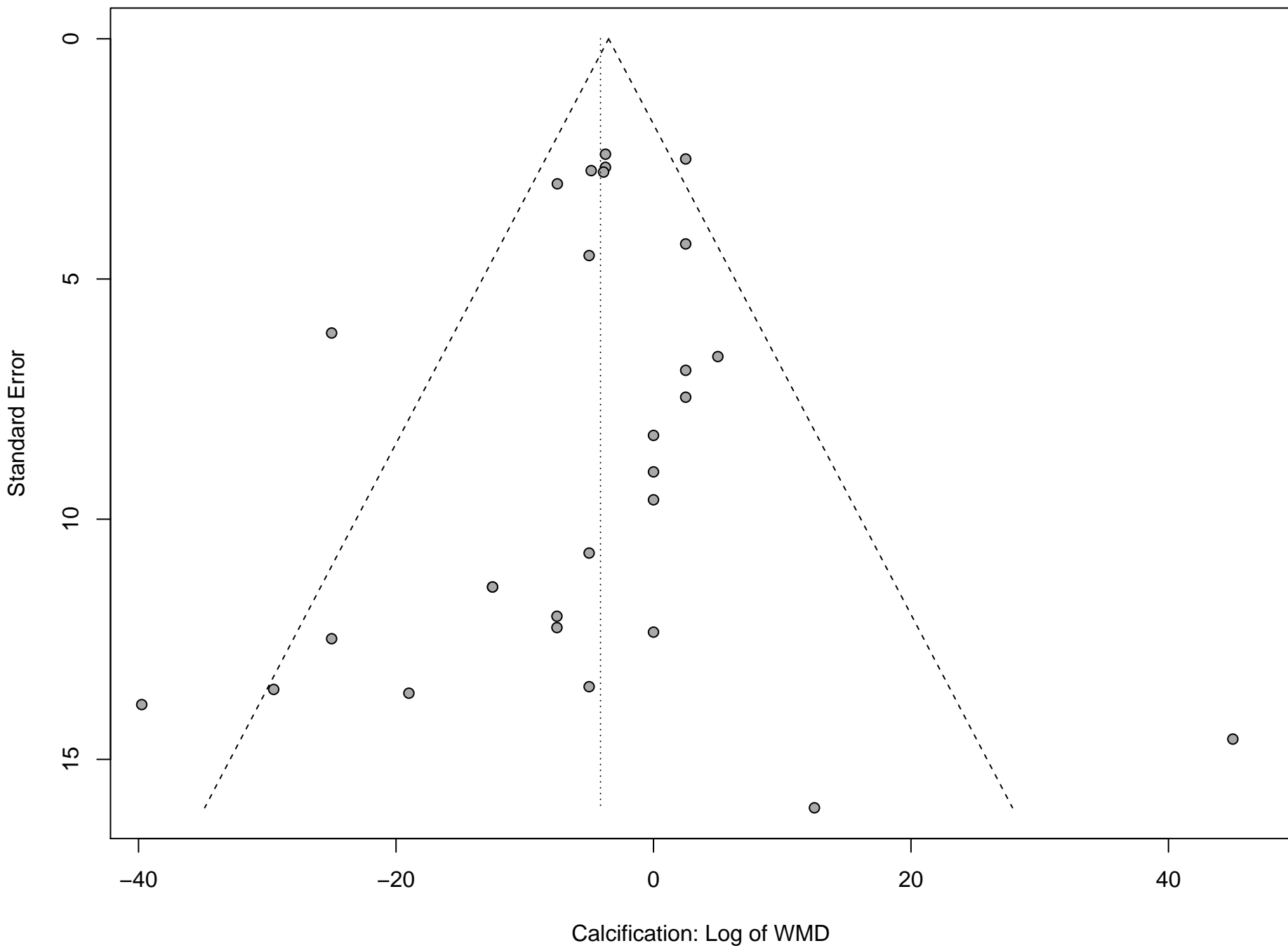

Supplement: Supplementary file 11 — Supplementary material 11 (PDF 10 kb) [file 395_2019_754_MOESM11_ESM.pdf]

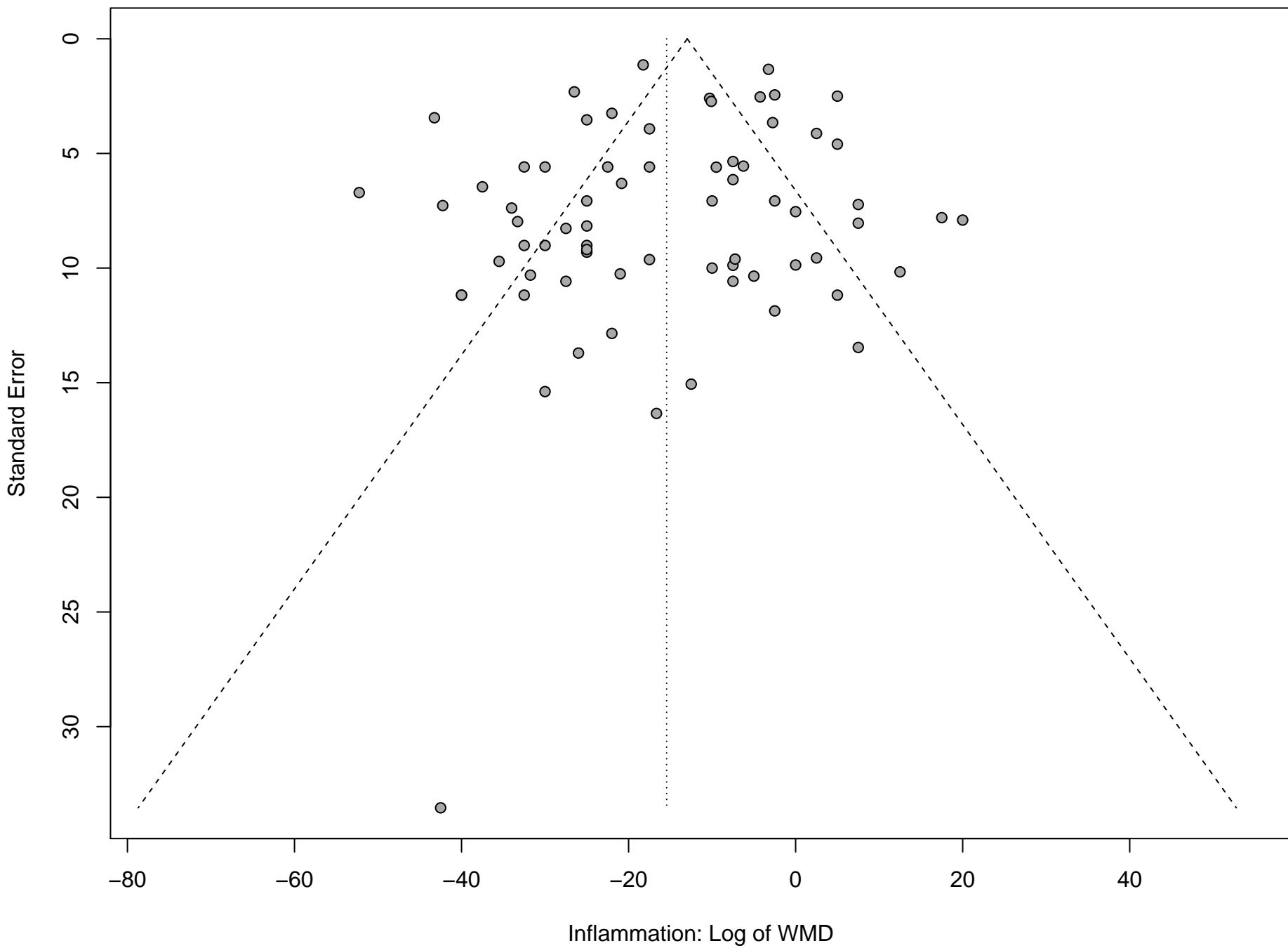

Supplement: Supplementary file 12 — Supplementary material 12 (PDF 10 kb) [file 395_2019_754_MOESM12_ESM.pdf]

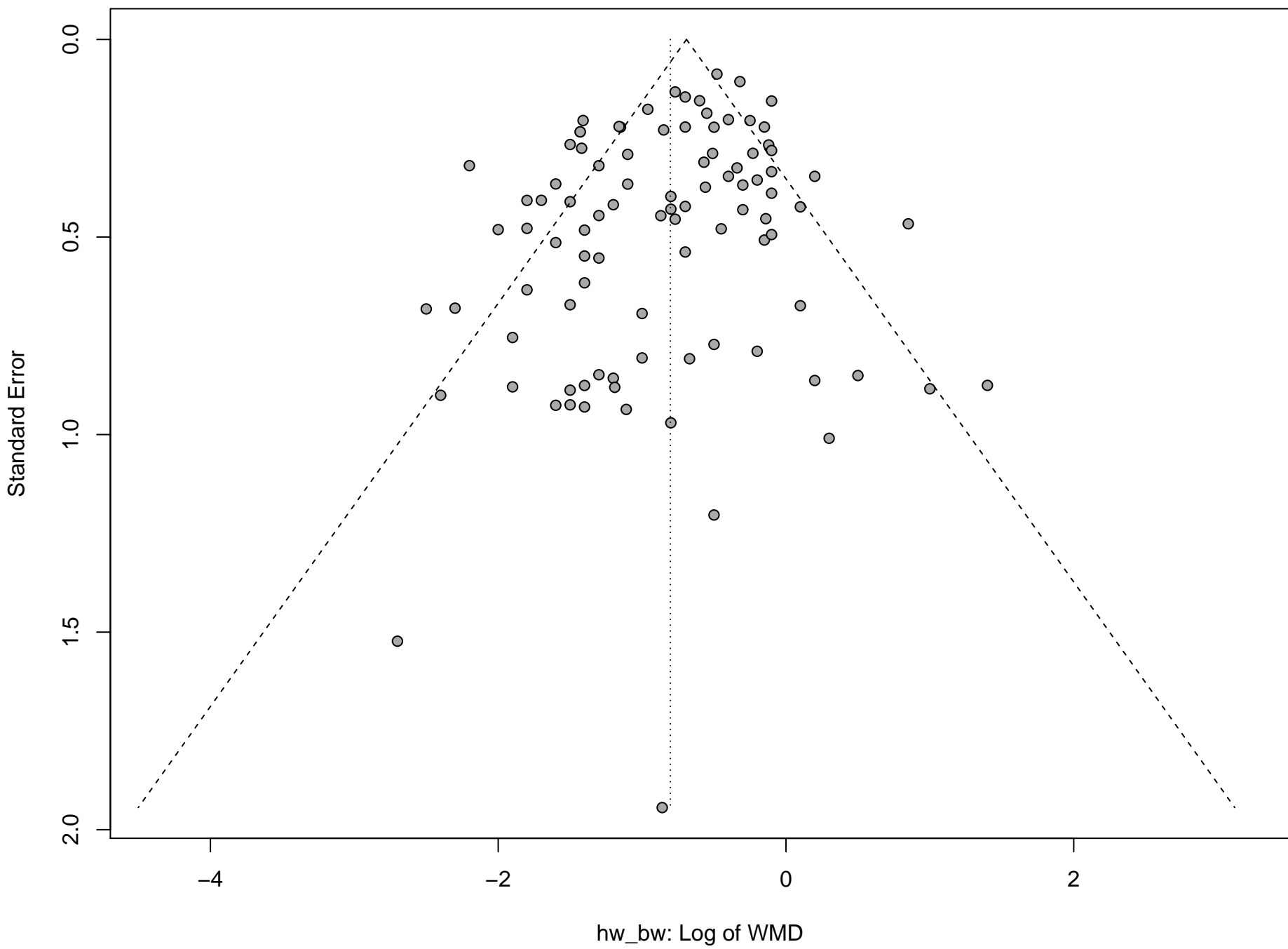

Supplement: Supplementary file 13 — Supplementary material 13 (PDF 11 kb) [file 395_2019_754_MOESM13_ESM.pdf]

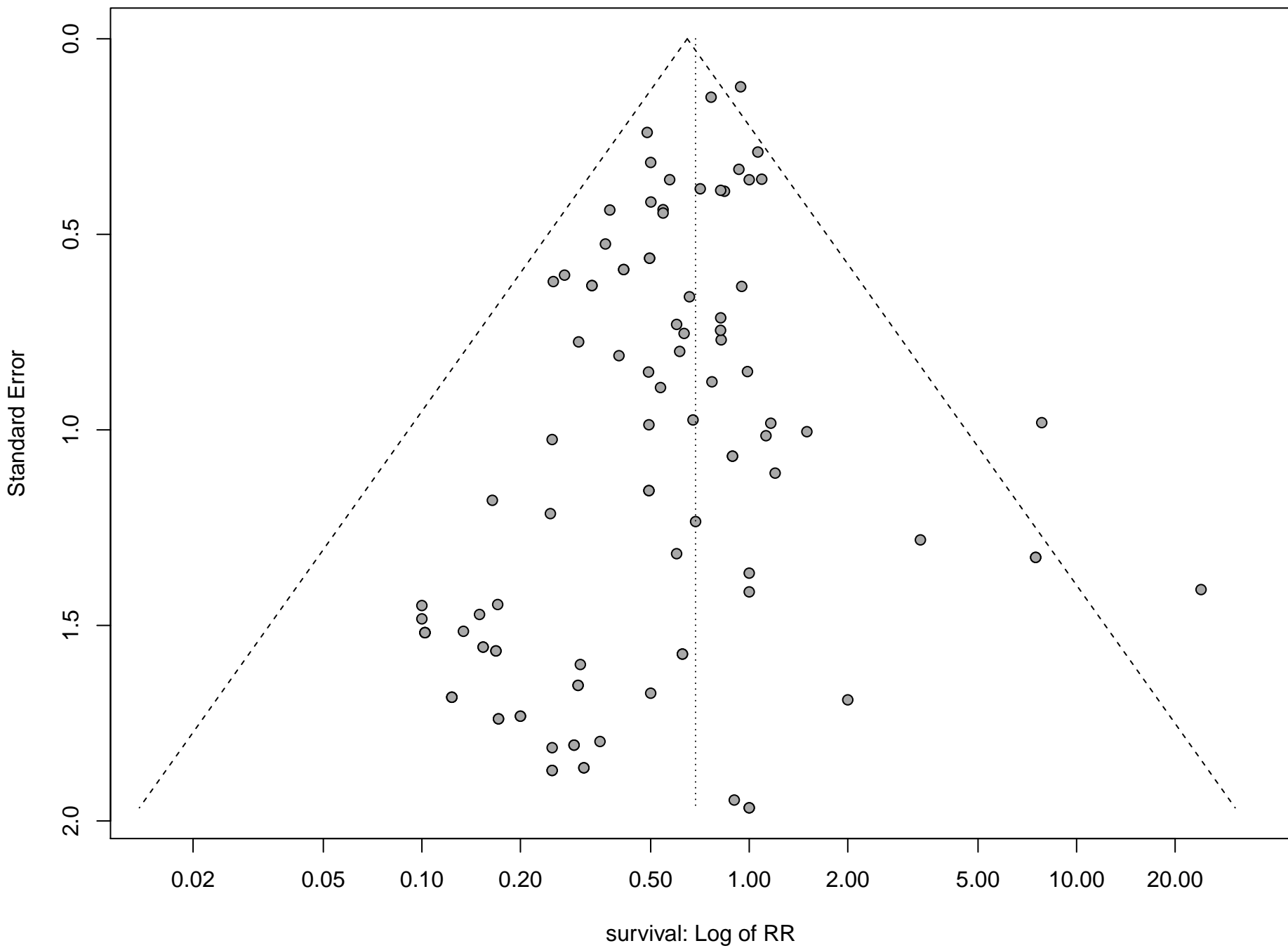

Supplement: Supplementary file 14 — Supplementary material 14 (PDF 11 kb) [file 395_2019_754_MOESM14_ESM.pdf]
