# Supplementary material for: Therapies to limit myocardial injury in animal models of myocarditis: a systematic review and meta-analysis
Source: Basic Res Cardiol. 2019 Oct 31;114(6):48. doi: 10.1007/s00395-019-0754-x (PMC6823299; doi:10.1007/s00395-019-0754-x)

Study	Intervention Total	Intervention Mean	Intervention SD	Control Total	Control Mean	Control SD	WMD	95% CI	Weight
**Drug Class = ACE inhibitor**									
Araki 1995 − a	6	15.00	7.5	1.20	32.50	10.0	−17.50	[−36.37; 1.37]	1.3%
Araki 1995 − b	6	22.50	10.0	1.20	32.50	10.0	−10.00	[−29.60; 9.60]	1.2%
Baba 2000 − d	6	68.50	17.0	4.60	75.75	14.2	−7.25	[−26.08; 11.58]	1.3%
Baba 2000 − e	16	75.75	14.2	4.60	75.75	14.2	0.00	[−14.78; 14.78]	1.5%
Bahk 2007 − b	18	15.25	12.7	10.00	67.50	19.0	−52.25	[−65.40; −39.10]	1.6%
Chen 2006	18	50.00	23.2	14.00	84.00	18.5	−34.00	[−48.47; −19.53]	1.5%
Godsel 2003	6	30.00	10.0	19.00	37.50	20.0	−7.50	[−19.54; 4.54]	1.6%
Rezkalla 1990(1)	16	45.00	17.8	16.00	54.50	13.6	−9.50	[−20.47; 1.47]	1.7%
Rezkalla 1990(2) − a	15	27.50	15.0	15.00	65.00	20.0	−37.50	[−50.15; −24.85]	1.6%
Rezkalla 1990(2) − b	14	40.00	22.5	14.00	32.50	15.0	7.50	[−6.67; 21.67]	1.5%
Rezkalla 1990(2) − c	15	42.50	32.5	15.00	45.00	32.5	−2.50	[−25.76; 20.76]	1.1%
Suzuki 1993 − a	12	44.00	23.2	4.00	65.00	15.5	−21.00	[−41.09; −0.91]	1.2%
Suzuki 1993 − b	13	33.25	24.5	4.00	65.00	15.5	−31.75	[−51.95; −11.55]	1.2%
Suzuki 1993 − c	12	29.50	20.2	4.00	65.00	15.5	−35.50	[−54.53; −16.47]	1.3%
Takada 1997 − a	20	27.50	10.0	15.00	45.00	12.5	−17.50	[−25.20; −9.80]	1.9%
Takada 1997 − b	44	20.00	12.5	41.00	22.50	10.0	−2.50	[−7.30; 2.30]	2.0%
Overall effect	.			.			−17.32	[−26.25; −8.38]	23.3%
Heterogeneity: $I^2 = 85\%$, $p < 0.01$									
**Drug Class = ARB**									
Araki 1995 − c	7	35.00	7.5	1.20	32.50	10.0	2.50	[−16.23; 21.23]	1.3%
Araki 1995 − d	7	25.00	10.0	1.20	32.50	10.0	−7.50	[−26.86; 11.86]	1.2%
Araki 1995 − e	5	45.00	10.0	1.20	32.50	10.0	12.50	[−7.42; 32.42]	1.2%
Baba 2000 − a	13	48.25	17.8	4.60	75.75	14.2	−27.50	[−43.71; −11.29]	1.4%
Baba 2000 − b	14	50.75	17.8	4.60	75.75	14.2	−25.00	[−41.00; −9.00]	1.4%
Baba 2000 − c	12	33.50	10.2	4.60	75.75	14.2	−42.25	[−56.51; −27.99]	1.5%
Bahk 2007 − a	19	34.21	22.9	10.00	67.50	19.0	−33.29	[−48.91; −17.67]	1.4%
Saegusa 2007	7	27.50	10.0	7.00	52.50	22.5	−25.00	[−43.24; −6.76]	1.3%
Sukumaran 2011(1)	8	25.00	84.8	8.00	67.50	42.4	−42.50	[−108.24; 23.24]	0.2%
Tanaka 1994 − a	10	52.50	12.5	5.00	65.00	32.5	−12.50	[−42.02; 17.02]	0.8%
Tanaka 1994 − b	12	35.00	17.5	5.00	65.00	32.5	−30.00	[−60.16; 0.16]	0.8%
Overall effect	.			.			−19.94	[−31.69; −8.19]	12.7%
Heterogeneity: $I^2 = 68\%$, $p < 0.01$									
**Drug Class = Beta−blocker**									
Gluck 2010 − a	19	35.42	15.0	22.00	56.25	24.8	−20.83	[−33.19; −8.47]	1.6%
Gluck 2010 − b	8	54.17	27.9	7.00	70.83	34.5	−16.66	[−48.69; 15.37]	0.7%
Li 2010 − a	24	25.50	2.2	7.00	43.75	2.8	−18.25	[−20.48; −16.02]	2.0%
Li 2010 − b	13	40.50	3.0	7.00	43.75	2.8	−3.25	[−5.86; −0.64]	2.0%
Li 2013 − a	8	47.50	24.0	8.00	73.50	30.4	−26.00	[−52.86; 0.86]	0.9%
Li 2013 − b	8	27.00	29.0	8.00	49.00	21.9	−22.00	[−47.18; 3.18]	1.0%
Nishio 2003 − a	5	37.50	11.2	2.50	55.00	4.0	−17.50	[−28.45; −6.55]	1.7%
Nishio 2003 − b	5	30.00	5.6	2.50	55.00	4.0	−25.00	[−31.93; −18.07]	1.9%
Nishio 2003 − c	5	27.50	5.6	1.67	50.00	6.5	−22.50	[−33.45; −11.55]	1.7%
Nishio 2003 − d	5	57.50	27.9	1.67	50.00	6.5	7.50	[−18.89; 33.89]	0.9%
Nishio 2003 − e	5	40.00	11.2	1.67	50.00	6.5	−10.00	[−23.86; 3.86]	1.5%
Rezkalla 1988 − a	10	5.00	7.9	10.00	0.00	0.0	5.00	[0.10; 9.90]	2.0%
Rezkalla 1988 − b	10	67.50	15.8	10.00	70.00	15.8	−2.50	[−16.36; 11.36]	1.5%
Rezkalla 1988 − c	10	72.50	31.6	10.00	67.50	15.8	5.00	[−16.91; 26.91]	1.1%
Rezkalla 1988 − d	20	27.50	33.5	20.00	7.50	11.2	20.00	[4.51; 35.49]	1.4%
Tominga 1991 − a	7	35.00	12.5	3.00	35.00	15.0	0.00	[−19.34; 19.34]	1.2%
Tominga 1991 − b	7	30.00	15.0	3.00	35.00	15.0	−5.00	[−25.29; 15.29]	1.2%
Tominga 1991 − c	10	25.00	12.5	12.00	32.50	12.5	−7.50	[−17.99; 2.99]	1.7%
Tominga 1991 − d	14	35.00	7.5	12.00	32.50	12.5	2.50	[−5.59; 10.59]	1.8%
Wang 2005 − a	5	25.00	5.6	1.67	55.00	6.5	−30.00	[−40.95; −19.05]	1.7%
Wang 2005 − b	5	22.50	5.6	1.67	55.00	6.5	−32.50	[−43.45; −21.55]	1.7%
Wang 2005 − c	5	30.00	11.2	1.67	55.00	6.5	−25.00	[−38.86; −11.14]	1.5%
Wang 2005 − d	5	32.50	11.2	1.67	72.50	12.9	−40.00	[−61.91; −18.09]	1.1%
Wang 2005 − e	5	32.50	11.2	1.67	72.50	12.9	−40.00	[−61.91; −18.09]	1.1%
Wang 2005 − f	5	40.00	11.2	1.67	72.50	12.9	−32.50	[−54.41; −10.59]	1.1%
Wang 2005 − g	5	35.00	11.2	1.67	67.50	9.7	−32.50	[−50.16; −14.84]	1.3%
Wang 2005 − h	5	37.50	11.2	1.67	67.50	9.7	−30.00	[−47.66; −12.34]	1.3%
Wang 2005 − i	5	42.50	11.2	1.67	67.50	9.7	−25.00	[−42.66; −7.34]	1.3%
Yue−Chun 2008 − a	6	26.25	4.0	3.00	69.50	5.2	−43.25	[−50.00; −36.50]	1.9%
Yue−Chun 2008 − b	6	66.75	5.0	3.00	69.50	5.2	−2.75	[−9.91; 4.41]	1.9%
Yue−Chun 2008 − c	6	23.00	2.8	3.00	49.50	3.5	−26.50	[−31.03; −21.97]	2.0%
Yue−Chun 2008 − d	6	45.25	3.8	3.00	49.50	3.5	−4.25	[−9.22; 0.72]	2.0%
Yue−Chun 2012 − a	8	28.25	9.2	8.00	34.50	12.7	−6.25	[−17.13; 4.63]	1.7%
Yue−Chun 2012 − b	8	40.75	18.4	8.00	65.75	18.4	−25.00	[−43.01; −6.99]	1.3%
Yue−Chun 2012 − c	8	25.00	0.0	8.00	47.00	9.2	−22.00	[−28.37; −15.63]	1.9%
Overall effect	.			.			−16.07	[−21.28; −10.86]	53.1%
Heterogeneity: $I^2 = 91\%$, $p < 0.01$									
**Drug Class = Direct renin inhibitor**									
Takamura 2016 − a	10	3.60	2.2	5.00	13.90	5.6	−10.30	[−15.39; −5.21]	2.0%
Takamura 2016 − b	6	3.80	2.7	5.00	13.90	5.6	−10.10	[−15.45; −4.75]	2.0%
Overall effect	.			.			−10.21	[−11.47; −8.94]	3.9%
Heterogeneity: $I^2 = 0\%$, $p = 0.96$									
**Drug Class = CCB**									
Wang 1997 − a	10	50.00	22.5	5.00	57.50	17.5	−7.50	[−28.23; 13.23]	1.2%
Wang 1997 − b	10	30.00	22.5	5.00	57.50	17.5	−27.50	[−48.23; −6.77]	1.2%
Xu 1992 − a	10	15.00	17.5	9.00	7.50	17.5	7.50	[−8.26; 23.26]	1.4%
Xu 1992 − b	13	37.50	22.5	14.00	20.00	17.5	17.50	[2.22; 32.78]	1.5%
Xu 1992 − c	10	20.00	10.0	9.00	15.00	10.0	5.00	[−4.01; 14.01]	1.8%
Overall effect	.			.			0.68	[−19.68; 21.03]	7.0%
Heterogeneity: $I^2 = 70\%$, $p = 0.01$									
**Overall effect**	**.**			**.**			**−15.44**	**[−19.30; −11.59]**	**100.0%**
Heterogeneity: $I^2 = 88\%$, $p < 0.01$									
Residual heterogeneity: $I^2 = 88\%$, $p < 0.01$									

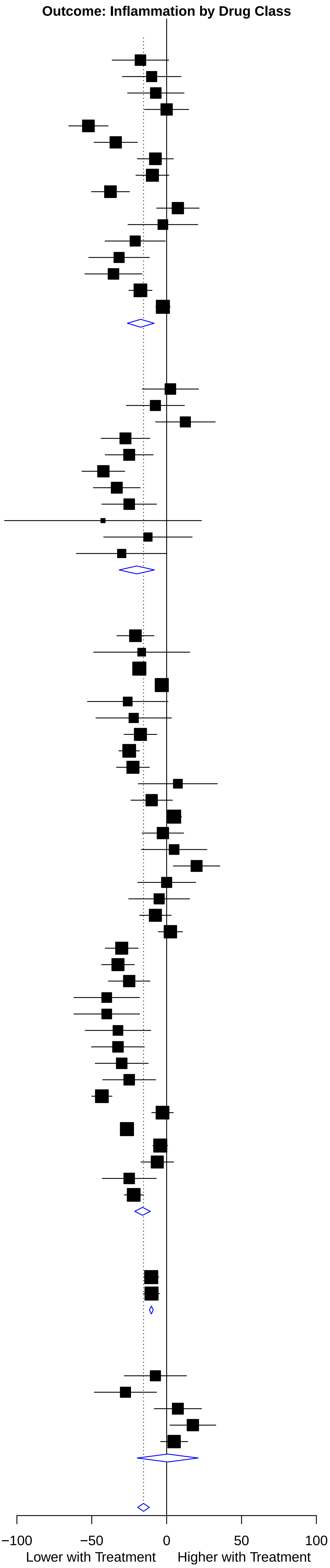

Supplement: Supplementary file 8 — Supplementary material 8 (PDF 12 kb) [file 395_2019_754_MOESM8_ESM.pdf]
