# Supplementary material for: Therapies to limit myocardial injury in animal models of myocarditis: a systematic review and meta-analysis
Source: Basic Res Cardiol. 2019 Oct 31;114(6):48. doi: 10.1007/s00395-019-0754-x (PMC6823299; doi:10.1007/s00395-019-0754-x)
Supplement: Supplementary file 16 — Supplementary material 16 (DOC 57 kb) [file 395_2019_754_MOESM16_ESM.doc]

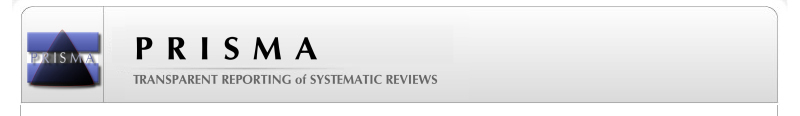
**PRISMA 2009 Flow Diagram**

**Screening**

**Included**

**Eligibility**

**Identification**

Records identified through database searching
(n = 347)

Additional records identified through other sources
(n = 0)

Records after duplicates removed
(n = 223)

Records screened
(n = 223)

Records excluded
(n = 132)

Full-text articles assessed for eligibility
(n = 91)

Full-text articles excluded, with reasons
(n = 39)

Not met inclusion criteria n=29

Not intervention vs. control n=8

Repeated data n=1

Missing data n=1

Studies included in qualitative synthesis
(n = 52)

Studies included in quantitative synthesis (meta-analysis)
(n = 52)
